# Molecular diagnosis in recessive pediatric neurogenetic disease can help reduce disease recurrence in families

**DOI:** 10.1186/s12920-020-0714-1

**Published:** 2020-05-13

**Authors:** Mahmoud Y. Issa, Zinayida Chechlacz, Valentina Stanley, Renee D. George, Jennifer McEvoy-Venneri, Denice Belandres, Hasnaa M. Elbendary, Khaled R. Gaber, Ahmed Nabil, Mohamed S. Abdel-Hamid, Maha S. Zaki, Joseph G. Gleeson

**Affiliations:** 1 Clinical Genetics Department, Human Genetics and Genome Research Division, National Research Centre, Cairo, 12311 Egypt; 2 Departments of Neurosciences and Pediatrics, Rady Children’s Institute for Genomic Medicine, Howard Hughes Medical Institute, University of California San Diego, La Jolla, CA 92093 USA; 3 Prenatal Diagnosis and Fetal Medicine Department, Human Genetics and Genome Research Division, National Research Centre, Cairo, 12311 Egypt; 4 Medical Molecular Genetics Department, Human Genetics and Genome Research Division, National Research Centre, Cairo, Egypt

## Abstract

**Background:**

The causes for thousands of individually rare recessive diseases have been discovered since the adoption of next generation sequencing (NGS). Following the molecular diagnosis in older children in a family, parents could use this information to opt for fetal genotyping in subsequent pregnancies, which could inform decisions about elective termination of pregnancy. The use of NGS diagnostic sequencing in families has not been demonstrated to yield benefit in subsequent pregnancies to reduce recurrence. Here we evaluated whether genetic diagnosis in older children in families supports reduction in recurrence of recessive neurogenetic disease.

**Methods:**

Retrospective study involving families with a child with a recessive pediatric brain disease (rPBD) that underwent NGS-based molecular diagnosis. Prenatal molecular testing was offered to couples in which a molecular diagnosis was made, to help couples seeking to prevent recurrence. With this information, families made decisions about elective termination. Pregnancies that were carried to term were assessed for the health of child and mother, and compared with historic recurrence risk of recessive disease.

**Results:**

Between 2010 and 2016, 1172 families presented with a child a likely rPBD, 526 families received a molecular diagnosis, 91 families returned to the clinic with 101 subsequent pregnancies, and 84 opted for fetal genotyping. Sixty tested negative for recurrence for the biallelic mutation in the fetus, and all, except for one spontaneous abortion, carried to term, and were unaffected at follow-up. Of 24 that genotyped positive for the biallelic mutation, 16 were electively terminated, and 8 were carried to term and showed features of disease similar to that of the older affected sibling(s). Among the 101 pregnancies, disease recurrence in living offspring deviated from the expected 25% to the observed 12% ([95% CI 0·04 to 0·20], *p* = 0·011).

**Conclusions:**

Molecular diagnosis in an older child, coupled with prenatal fetal genotyping in subsequent pregnancies and genetic counselling, allows families to make informed decisions to reduce recessive neurogenetic disease recurrence.

## Background

Congenital malformations account for ~ 20% of infant mortality and ~ 18% of pediatric hospitalizations [1–3]. Many are recessive disease syndromes that are severe, untreatable, and adversely affect quality or length of life, prompting parents to seek prenatal counseling for future pregnancies. Over 3000 individual genes are currently linked with Mendelian disease, and this list continues to grow rapidly [4]. Furthermore, there is growing consensus around standardized interpretation of genetic variants within a clinical context [5] and diagnostic decision support software (DDSS) can provide an additional level of certainty as to pathogenicity [6, 7].

Autosomal recessive diseases account for ~ 26% of severe pediatric conditions undergoing diagnostic sequencing [8], and impart a standard 25% recurrence risk. Most of these diseases are not evident with fetal ultrasound prior to 20 gestational weeks (GW), especially neurological conditions, since much of human brain development occurs after mid-gestation [9, 10]. Next generation sequencing (NGS) diagnostic approaches are becoming more common for children with likely recessive diseases [11], and families receiving a molecular diagnosis for their child could benefit from this information for prenatal testing for their subsequent pregnancies. However, there are few reports documenting population-level effectiveness of fetal genotyping supporting the prevention of disease recurrence through elective termination of pregnancy (eTOP), and despite this approach gaining acceptance, each prenatal center follows its own best-practices.

Here, we leverage family-specific NGS sequencing, combined with fetal targeted genotyping to measure the impact of this approach on the eventual disease recurrence risk in families, by providing predictive affectation status information to couples carrying a pregnancy that were at 25% risk for disease recurrence. We show that accurate molecular diagnosis in an older affected child in a family can support prenatal diagnosis (PND) and genetic counseling for families returning with a subsequent pregnancy, which can influence informed decisions about pregnancy termination, ultimately leading to reduced disease recurrence.

## Methods

### Study design

We studied the utility of molecular diagnosis in children, using exome or genome sequencing, on the potential to support the reduction of disease recurrence in families. Most recessive pediatric brain disease (rPDB) seen in our center do not have obvious structural fetal anomalies, rendering fetal imaging sub-optimal for determining affectation status. We reasoned that, as cases received a molecular diagnosis, families would be interested in using this information to distinguish affectation status of future pregnancies, that could ultimately impact their decisions about eTOP of fetuses predicted to display disease. Our framework was based on assessing the potential impact of NGS diagnosis of an older child on the recurrence risk in the same family, compared with historic 25% risk. We first validated the use of diagnostic sequencing, benchmarked diagnostic decision support software for mutation validation using a signs/symptoms approach, and utilized this information in counseling families.

### Study cohort

Information on 1172 families that were seen from 2007 to 2016 at a single Egyptian national referral center was reviewed. These families had 92% reported parental consanguinity and one or more children suffering from a severe undiagnosed neurogenetic disorder. Inclusion criteria for the study were (a) presence of a likely rPBD in a child, (b) condition presenting before the age of 2 years and (c) in families with more than one affected child, both children had a nearly-identical clinical presentation. All living affected children from families meeting study criteria were examined by a clinical geneticist and a child neurologist with a review of developmental trajectory, and history of medications, epilepsy, intellectual disability (ID), growth parameters, radiological, electrophysiological (EEG, NCV, EMG), metabolic testing, and detailed family history. DNA sampling in the form of blood or saliva was obtained for the entire nuclear family including all affected and unaffected children and their parents, and in some cases genetically informative extended relatives. OMIM features with standard diagnostic criteria were used to determine affectation status of newborn children, as assessed by the neurologist and geneticist.

### Family molecular genetic testing

All 1172 families were subject to exome sequencing between 2010 and 2016 with a goal of identifying the molecular cause of the disease in each case. Exome sequencing was performed on samples from at least one affected member per family, but usually included two affected children or one affected child and the parents for singleton cases as described [12].

### Exome and genome sequencing

Exome on DNA samples was performed using IDT Exome capture kit. Hybrid selection libraries covered > 80% of targets at 20x with a mean target coverage of >80x. The exome data were demultiplexed and each sample’s sequence was aggregated into a single BAM file. Genome on DNA samples (500 ng to 1.5 μg) was performed using a PCR-free protocol. These libraries were sequenced on the Illumina HiSeq 4000 with 150-bp paired end reads and target mean coverage of >30x.

A strict set of criteria was used to determine the causal variant in each family (Section S1). The results of the research sequencing were provided to the medical team and subsequently to the families only if (a) the variant was reported as an OMIM gene implicated in a severe rPBD, (b) met ACMG guidelines as a pathogenic or likely pathogenic variant [13] and (c) was confirmed in a clinical laboratory by targeted sequencing. These families received genetic counseling and were informed about prenatal or preimplantation genetic diagnosis, gamete donor, adoption, and treatment/therapy options for subsequent affected children. Families were invited to return to clinic if they become pregnant in the future.

### Genome-phenome validation of molecular diagnosis

All families that received molecular diagnosis and returned to the genetics clinic while in the first trimester of pregnancy were followed up in this study. Families interested in prenatal genetic testing were screened by the team and subsequently consented for PND. For these families, an additional unbiased comparison between genotype and phenotype within the clinical context using SimulConsult® (SC) diagnostic decision support software (DDSS) was performed (Figs. S1–2). Quantitative assessment of ‘zygosity pertinence’ was calculated, as a joint probability of a match between signs/symptoms and gene mutation. Cases with less than 90% pertinence were excluded from PND. This additional step was performed in order to orthogonally evaluate the pathogenic variant that was identified using ACMG guidelines.

### Integration of genotyping results into clinical practice

Families that opted to conceive again, and succeeded in becoming pregnant after receiving genetic diagnosis, returned to clinic between 2013 and 2017 and were offered the possibility of PND under conditions: (a) a singular genetic cause for the affected child could be identified with certainty, (b) the genetic cause could be confirmed through DDSS that incorporated the latest published gene discoveries and associated disease phenotypes, (c) the pregnancy was prior to 16 GW at the time of testing, (d) amniocentesis could be performed at the clinic with a reported standard risk for all complications of < 3%, (e) the condition in the older child was considered untreatable and likely to severely limit lifespan. Care was taken to avoid maternal blood contamination. Fetal DNA was extracted and genotyped using Sanger sequencing for zygosity and segregation in the family in both parents and all available older children. Assessment of maternal DNA contamination was performed using SNP analysis. Analysis of the results were discussed with the family to convey available options, and risks/benefits of eTOP in families requesting this information. In all cases, families had one or more prior affected offspring, and thus had an appreciation of the natural history of the disease that potentially impacted their fetus.

### Safety

All families with pregnancies received counseling on risks-to-benefits for prenatal testing procedures. When amniocentesis was elected, fluid sampling was performed under ultrasonic guidance at 15–16 GW to minimize the risk of complications while obtaining fetal genotyping results as early as possible. Families deciding on eTOP were referred to certified abortion clinics with standard low complication rates.

### Outcome measures

The primary outcome measure in this study was the affectation status of live-born children that were genotyped prenatally through amniocentesis. Phenotype assessment was performed after birth, at 12 months, and at 36 months (where possible) and included an evaluation of growth parameters, psychomotor development, vision, hearing and measures of pertinent features that distinguished the clinical presentation of the older offspring in the family, adjusted for age-of-onset of symptoms on a case-by-case basis. Secondary outcome measures included complications of pregnancy, amniocentesis, abortions and deliveries and included spontaneous pregnancy termination, Rh-incompatibility, severe bleeding or loss of amniotic fluid, post-procedure infections, severe cramping and fetal trauma.

### Power calculations

Given the expected 25% recurrence risk for each subsequent pregnancy in recessive disease, our study cohort of 67 births following genetic counseling provided power of 92.2% (with a two-sided type I error of 5%) to detect a significant effect of prenatal testing on reduction in the primary outcome measure, with the observed 67% decision to terminate pregnancies of an affected fetus corresponding to a change in outcome measure from predicted 17 out of 67 affected births to observed 8 out of 67 affected births (Fig. S3).

### Statistical analysis

Comparison of categorical variables using the binomial goodness-of-fit test was performed on a 2 × 2 contingency table with the following cells: expected number of affected children (25%), expected number of unaffected children with no prenatal testing (75%), number of observed affected children with prenatal testing, number of observed unaffected children with prenatal testing. Negative predictive value (NPV) was calculated according to the formula NPV = number true negatives / (number true negatives + number false negatives). Positive predictive value for the full dataset could not be determined due to inability to phenotype eTOPs..

## Results

### Identification of disease-causing variants in study participants

**Fig. 1:**
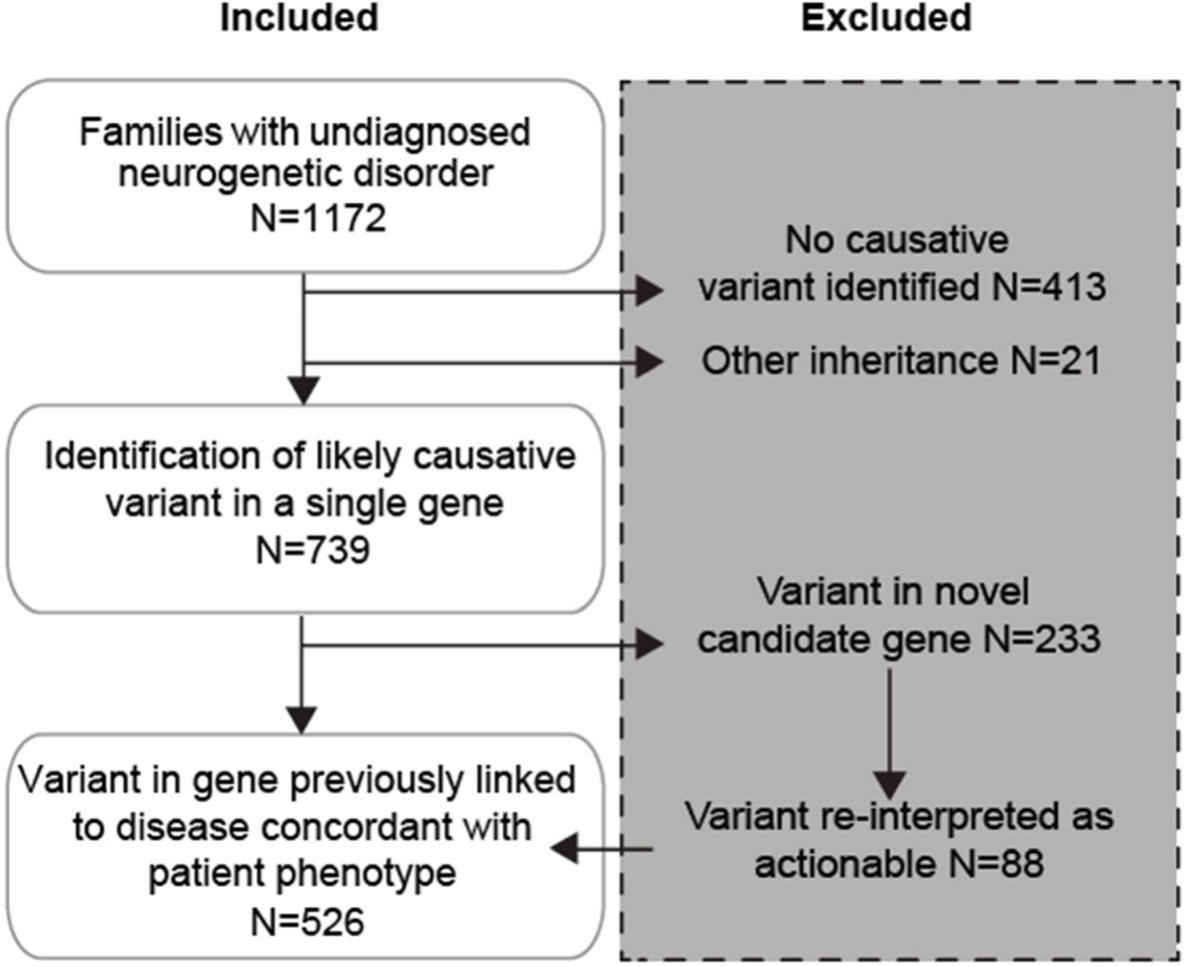
Genetic screening for disease-causing biallelic variants. All 1172 families presented with a likely autosomal recessive disorder for genetic counseling from 2010 through 2016, and underwent exome sequencing. In 413 families, no genetic diagnosis could be made despite ‘reflex to trio’ and ‘reflex to genome’. In 21 families a disease-causing variant was identified in a gene with a different inheritance pattern (i.e. de novo or X-linked), and both of these groups were excluded. In 739 families, a single variant was identified that was likely causative. Of these 233 occurred in genes not previously linked to disease and were initially excluded but in 88, subsequent variant re-interpretation following new publications allowed families to re-enroll. In all, 526 families with a pathogenic or likely pathogenic variant in a gene previously linked to a disease that was concordant with the phenotype of the older child or children in the family. *(N - number of families)*

In this retrospective study, 1172 families with a likely rPBD were previously enrolled at a single referral site in a genetic study aimed at evaluation of the genetic cause(s) (Fig. 1). Positive family history of consanguinity was reported in 92% of families. These families presented with one or more infants or children with a severe neurodevelopmental disease that significantly impacted health or predicted shortened lifespan. Fully 73% of families already had two or more affected children, consistent with a recessive mode of inheritance. Exome sequencing was performed (see Methods) with standard clinical interpretation of potentially damaging variants, including recessive bi-allelic, dominant, X-linked, de novo, post-zygotic (i.e. somatic) and structural variants. Through assessment of segregation of the variant(s) in the family and analysis of splicing in patient cell lines, evidence that the variant fulfilled ACMG guidelines was determined. In 37% of families, lack of a molecular diagnosis based upon exome led to subsequent genome sequencing in the trio, which in 7% of cases led to the identification of a likely disease variant that was missed from exome, mostly as a result of reduced exome coverage in certain regions as reported [14].

In 413 families (35.2%), no likely-causative variant (LCV) could be identified, despite a ‘reflex to trio’ and ‘reflex to genome’ approach (see below), and were thus excluded from further study. Twenty-one families (1.7%) displayed variants consistent with other forms of inheritance (i.e. de novo or X-linked) and were excluded from study in order to limit the study to recessive disease. Ultimately in 739 families (63.0%) a single biallelic LCV was identified in 441 total genes. Of these, 233 families had LCVs in 197 novel genes not implicated in disease prior to the return of molecular testing results, classified as a ‘variant of unknown significance’ (VUS) in a ‘gene of unknown significance’ (GUS). As new disease genes were constantly published, 88 of the VUS/GUSs were reinterpreted as pathogenic or likely pathogenic (ACMG guidelines) within a clinical context upon annual re-review, and the families re-enrolled in the study (Fig. 1). Similarly, through re-analysis of exome and genome data, many families with initially negative sequencing results were subsequently solved according to ACMG guidelines and re-enrolled in the study.

### Counseling parents for genetic testing results

Five hundred twenty-six families had a pathogenic or a likely pathogenic variant residing in a gene in which biallelic variants have been linked to a rPBD with a reported phenotype that closely matched the documented family phenotype, according to ACMG guidelines. All clinically relevant variants were confirmed in a clinical laboratory according to local requirement, confirmed results results were reviewed by clinical geneticists and shared with families. Parents of these 526 families were contacted to convey the sequencing results and for genetic counseling regarding prospective family planning. Each family was counseled on the prognosis and currently available treatments and/or supportive therapies for the disorder, on the available options of PND procedures and the associated risks.

### Fetal genotyping

Of 526 families that received molecular diagnoses, 91 (17.3%) conceived again, and returned for prenatal counseling within a four-year time frame between 2013 and 2017 with 101 pregnancies, as 10 families returned with two pregnancies each. There were 18 (3.4%) families lost to follow-up. The remaining 417 (79.3%) families upon follow up, either decided against future pregnancies or did not succeed in becoming pregnant during this study period. This was likely in part due to the long interval between recognition of disease in their child and the molecular diagnosis (average 2.9 years). There were no other pregnancies reported by these families in this study.

For the 101 pregnancies seeking PND, evidence supporting pathogenicity of their variants is provided in Table S1. Risks and potential false-positive and false-negative results of PND were discussed prior to ultrasound-guided amniocentesis. In 17 pregnancies, PND was declined at that point. Since for many families, the molecular diagnosis was made several years prior to the current pregnancy, and more detailed information became available both on clinical presentation and genetic variants associated with these disorders, re-evaluation of the data was appropriate. DDSS was used for those families seeking PND to increase certainty of genotype to match the phenotype in each case (Table S1). Due to the possibility that an incorrect provisional diagnosis of older offspring could have inadvertently influenced the genetic findings, we opted for a signs/symptoms-based DDSS, to provide a quantitative joint probability for a match (i.e. zygosity pertinence) with the full NGS variant table (Fig. S1-S2). In all families (84 pregnancies) pertinence was > 95% between signs/symptoms and molecular cause (Data S1), and none were excluded based upon this additional validation step.

### Clinical features in families that received fetal genotyping

**Fig. 2:**
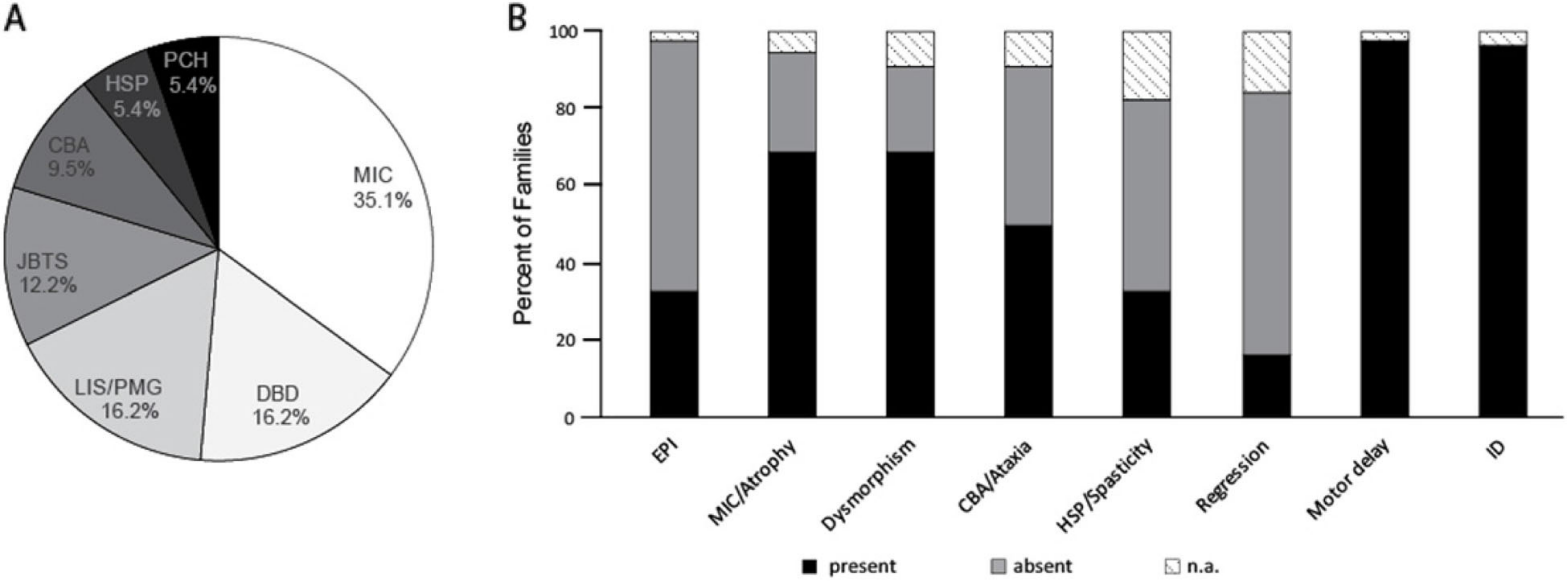
Classes of clinical diagnoses and percentage of cases with major disease features. **a** Distribution of clinical diagnoses based on a leading clinical or morphological feature in 74 of 526 families with identified molecular diagnosis in a child seeking prenatal counseling for one or more pregnancies. Most common diagnosis was microcephaly followed by degenerative brain diseases (DBD) and lissencephaly/polymicrogyria (LIS/PMG). **b** Percent of cases with corresponding symptom. Most cases presented with motor developmental delay and intellectual disability. Abbreviations: CBA-cerebellar atrophy/hypoplasia, DBD-degenerative brain disease, EPI-epilepsy, HSP-hereditary spastic paraplegia, ID-intellectual disability, JBST-Joubert Syndrome, LIS-lissencephaly, MIC-microcephaly, PCH-ponto-cerebellar hypoplasia, PMG-polymicrogyria, n.a.-data not available

Of the 74 families that received PND, clinical manifestations were assigned to one of 7 disease categories, based on presenting clinical features: (a) microcephaly (MIC), (b) degenerative brain disease (DBD, including progressive epileptic encephalopathy), (c) lissencephaly/polymicrogyria (LIS/PMG), (d) Joubert syndrome and related disorders (JBTS), (e) cerebellar atrophy (CBA), (f) hereditary spastic paraplegia (HSP) and (g) ponto-cerebellar hypoplasia (PCH) (Fig. 2). Example pedigrees, photographs and brain scan images for each disease category are shown (Fig. S3).

For each family, available clinical data included signs and symptoms of general and neurological dysfunction as well as degree of ID (Table S2). All children presented with moderate to severe motor delay and over 90% had profound or severe ID, which was likely to be associated with congenital syndromes and often involved multisystem abnormalities predicted to limit lifespan. Each case presented at least two neurological features, with the majority displaying 4 to 5 features (Fig. S4), consistent with severe rPBD. Microcephaly and dysmorphic features were observed in the majority of cases (> 60%). Features such as spasticity, ataxia, seizures and developmental regression were noted.

### Correlation of fetal genotyping with pregnancy outcomes

**Fig. 3:**
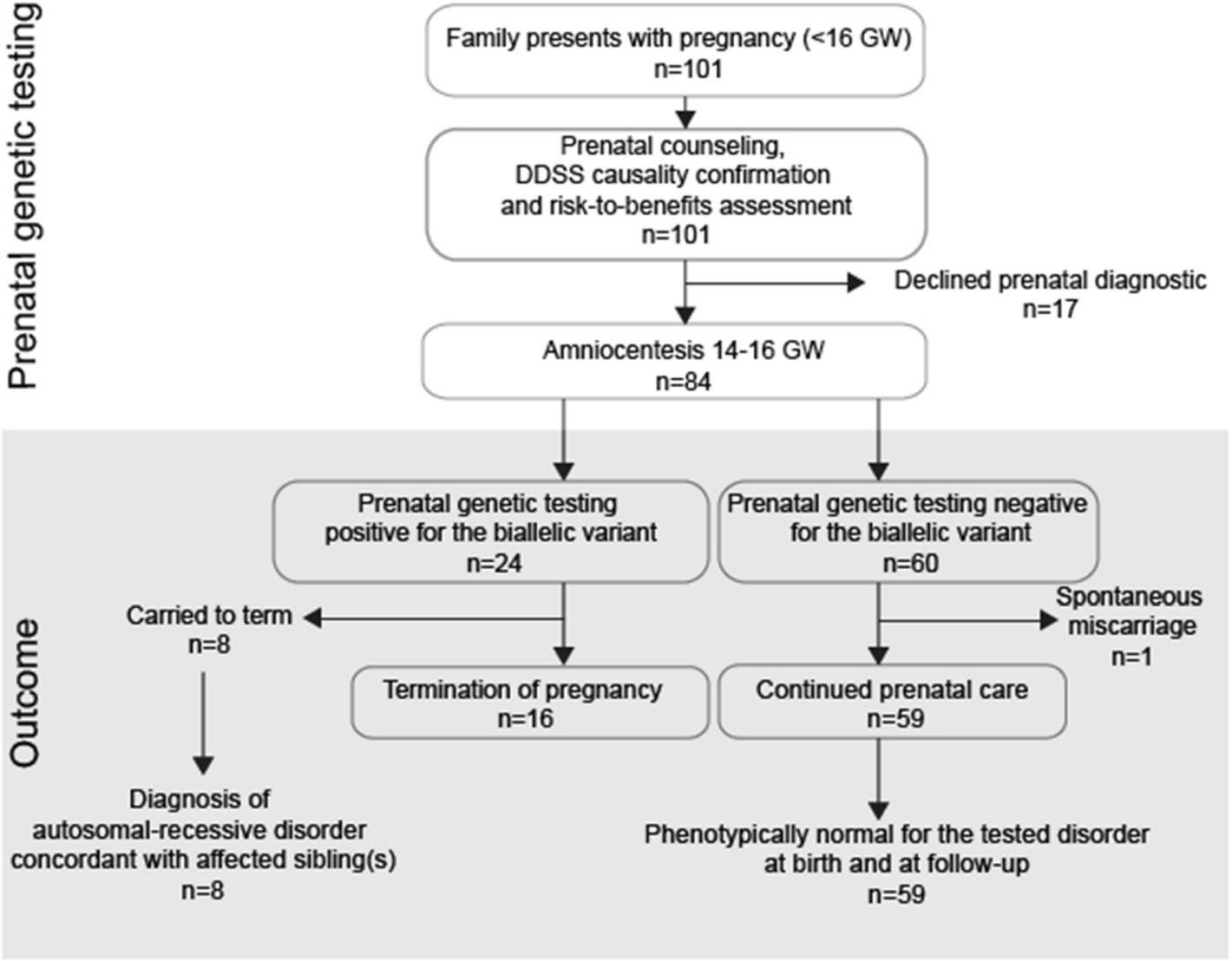
Prenatal genetic testing with workflow and outcome of pregnancies with previously identified disease-causing variant. Families with 101 pregnancies presented at < 16 gestational weeks (GW) from 2013 through 2017. Of these, 17 declined prenatal diagnostics after risk-to-benefit assessment. Diagnostic decision support software (DDSS) was applied to verify the molecular diagnosis and confirmed the relationship between genotype and phenotype in the remaining 84 pregnancies that received amniocentesis at 14–16 GW for targeted fetal genotyping. Of these, 24 tested positive and 60 tested negative for the biallelic disease-causing variant. Eight of the 24 pregnancies that tested positive were carried to term, and in each case the child was diagnosed with a disease concordant with the older affected sibling or siblings. Of 60 pregnancies that tested negative, one spontaneous miscarriage was excluded from evaluation, and 59 pregnancies were carried to term. In each of these 59 cases, the child was unaffected at birth and remained unaffected by the time of the last clinical follow-up for the tested disorder. Binomial goodness-of-fit test showed statistically significant difference in the primary outcome measure (95% CI, 0.04 to 0.20; *P* = .011) *(n - number of individual pregnancies)*

Amniocentesis followed by Sanger sequencing of the variant in the extracted DNA compared with DNA of the rest of the family, including both parents and all available children (both affected and unaffected) allowed for accurate genotyping of all 84 pregnancies. Secondary outcome measures were all negative for losses of pregnancy, extensive bleeding or post-procedural complications, there was no detectable maternal blood contamination in collected fetal samples, and no equivocal genotyping results. There were 39 that genotyped as heterozygous for the pathogenic variant and 21 as homozygous reference, all predicted to be unaffected by the disease for which they were tested. Upon follow up, we found that none of these children showed evidence of the tested disease at the latest evaluation (Fig. 3 and Table S3). Among these were one case of a spontaneous loss of pregnancy that was not attributed to the amniocentesis procedure, and one with a skeletal deformity (absent sternum) at birth that was unrelated to the phenotype of the affected older sibling, and that remained otherwise neurologically normal. None of the children showed any structural deformities attributed to the amniocentesis procedure. Ten families subsequently became pregnant again during the study period, and had a second pregnancy undergo PND (Table S3).

Out of 24 that tested positive for the biallelic pathogenic variant, 16 ultimately decided on eTOP and 8 carried the pregnancy to term. None of the 16 women experienced notable complications of the eTOP. Due to local customs and medical practice, assessment of fetal tissue for malformations or genotyping could not be performed. The 8 that were carried to term each returned to follow up clinic, and each was assessed for clinical features observed in the older affected sibling(s), according to OMIM criteria for each of gene/mutation, in an age-dependent and organ-specific fashion. Each displayed a phenotype concordant with that of the older affected sibling(s) and was ultimately diagnosed as affected by the same disease (Table S4). Thus, while positive predictive value (PPV) of PND could not be assessed, negative predictive value (NPV, i.e. probability that offspring does not have disease given negative PND) was 100%.

### Statistical evidence for reduction of recurrence in families

Tests of significance were limited to the 101 pregnancies that returned to clinic in molecularly diagnosed cases, rather than in the full cohort of 1172 cases. Power analysis to detect significant differences in outcome was performed by varying the number of pregnancies and the proportion of predicted affected pregnancies terminated. We determined that a sample size of 80, given eTOP decision in 70% of affected pregnancies, would yield > 90% power to detect a significant difference in outcome from this study (Fig. S5). The observed 12% affected and 88% unaffected for the tested disease live-births were compared with the expected 25%:75% affected:unaffected, respectively (using normative recessive inheritance rates) and showed a statistical difference in the observed percent of liveborns affected by disease compared with expected (95% CI, 0.04–0.20; *P* = 0.011). The observed ratios were also compared with the measured proportions (i.e. 28.6% tested positive for the biallelic disease-causing variant, whereas we expected 25%), using a two-tailed binomial test for the goodness-of-fit. This resulted in a *P*-value of 0.0017, indicating a significant reduction in observed recurrence risk over expected in this study.

### Assessment of decision regret by parents

We assessed parental experiences with their decision about eTOP at ~ 6 months after the predicted due date for all 24 pregnancies. None of the 8 parents of pregnancies carried to term had feelings of regret about their decision, despite clinical features of disease apparent in most. These 8 couples cited several reasons why eTOP was not pursued, including social, logistical, religious or economic reasons contributing to their decision. None of these couples were attempting to become pregnant again at the time of the 6-month follow up, and no further pregnancies were observed from these 8 couples. None of the 16 parents of the eTOPs had feeling of regret about their decision at follow-up. Several of these couples were attempting to become pregnant again by the 6-month visit, and several families had a second fetus undergo PND during the study period (Table S3).

## Discussion

Despite the expansion of NGS into the clinic, and the improvements in molecular diagnostic rates for neurogenetic disease [13, 15] successful utilization of this information to improve outcomes or reduce recurrence has been limited [16, 17]. Using NGS from families recruited for discovery of genetic causes of severe or lethal rPBDs allowed for: (a) unbiased assessment of the pathogenicity of the variant with DDSS, (b) ethical, social, and medical risk considerations, (c) PND of a genetically susceptible fetus, (d) referral for eTOP of genetically affected fetuses. Many single-gene disorders do not show gross structural defects that would be identified by fetal imaging in the first trimester, and PND is a valid alternative to identify fetuses that are predisposed to show severe disease.

PND could be applied to future families in which the cause for the index recessive case is determined by targeted panel, exome or genome sequencing. Since the current diagnostic rates with these techniques vary between ~ 25 and 60% [8, 18–20], this approach could impact a substantial number of families with recessive disease that undergo sequencing. Limitations of this study are that only 91 of the 526 families that received a molecular diagnosis returned to clinic with a subsequent pregnancy, which is only 17% of those that could have benefited. We were also not able to assess the 417 families, where no molecular cause was identified, on whether family planning decisions would have been different if a molecular cause had been identified. Nevertheless, our results could help to further justify use of NGS in genetic diseases beyond clinical management of the affected proband or affected siblings, to include the potential benefit for future family planning [16]. Even though reproductive specialists often provide genetic testing based upon experience, widespread acceptance by public health authorities and insurance providers could benefit from future prospective studies demonstrating NGS implementation that ultimately lead to reduced disease recurrence.

Family planning and counseling are some of the most challenging issues facing the medical team. Decisions ultimately lie with the mother or couple, but can be influenced by numerous ethical, social, familial, legal and logistical considerations. The concepts surrounding consent for PND are evolving in collaboration between families, physicians, sociologists and ethicists to ensure that couples are provided accurate assessment of risks and benefits [21]. By limiting this study to a single referral center where the practice was consistent among clinicians and genetic counselors, families received consistency in the approach. It will be important to evaluate psychological issues, inadvertent delays in the process, and potential barriers to service, to minimize the likelihood that families cannot receive information and medical care that they seek.

The NPV of 100% that we observed may not be representative for larger applications of this design, taking into account the 0.1% estimated sequencing error and the possibility of human error, which would negatively impact diagnostic accuracy. Additionally, PPV could not be calculated due to the inability to independently confirm the phenotype of fetuses predicted to be affected, and the absence of clinical features prior to eTOP in nearly every case. Minimal variability in expressivity of each condition in each family was observed, where there were two or more affected children diagnosed prior to PND, and each of the 8 children later diagnosed as affected displayed features nearly identical to their older affected sibling(s).

PND performed in the study was targeted to the previously identified biallelic variant in families with a previously affected offspring, and as such at best could only be used to prevent recurrence of disease. The benefit of this approach is that each family had prior experience with the disease in their older offspring, which may have facilitated the decision about subsequent pregnancies. An imperfect comparison can be made with approaches that utilize premarital carrier sequencing or fetal NGS in naïve families even in the presence of congenital malformations, where experience with the anticipated disease is minimal or nonexistent.

Although the method chosen for fetal sampling was amniocentesis, chorionic villus sampling (CVS) is a comparable alternative, if available, as it can be performed earlier than amniocentesis. This would allow for earlier return of results, provide families with additional time to consider options, and open up less invasive eTOP options, while only minimally increasing risk of complications [22]. Additionally, the variants identified in probands could be alternatively used in preimplantation genetic diagnosis (PGD) to reduce recurrence risk in subsequent pregnancies. PGD is not widely available in Egypt where this study occurred, limiting its impact, and suffers its own limitations [23–25].

In our study we focused on direct Sanger sequencing of the pathogenic allele in the fetus that was previously identified in an older sibling(s). An alternative approach utilizing NGS of fetal DNA directly in disease-naïve families would expand the benefit of PND [26], but suffer from nonpenetrant variants, as was recently reported for cystic fibrosis or Krabbe diseases [27, 28]. Consanguineous families might especially profit from this approach due to the higher risk of recessive diseases, or risk of more than one recessive disease [29].

Recent publications highlight that between 4.9–13% of cases show two different diseases or disease-causing mutations in the same child [30, 31]. Such occurrence would reveal a limitation of our approach, unless both diseases were recognized prior to fetal genotyping. We did not observe evidence of multiple single-gene disorders in any of our families, probably in part because families with clinically discordant siblings were excluded. We observed a skeletal deformity in an otherwise healthy case newborn, suggesting a second genetic condition, but the genetic origin of the skeletal deformity was not investigated. Ideally, assessment of all such conditions relevant to the fetus could be performed simultaneously.

Premarital genetic counseling is seen by some as a superior method to prevent disease, and is in reach for some common genetic alleles in certain populations [32, 33]. For instance, recessive traits in the Ashkenazi population, like Tay-Sachs disease (1:27–1:30 carrier frequency) formed the basis for genetic premarital efforts that reduced the incidence by > 90% [34]. With an expansion of NGS accessibility, preconception carrier screening for the majority of inherited diseases will be feasible, but will suffer from imperfect predictive power in many instances. Our study is unique in that it provides a practical approach for predicting the phenotype of fetuses in families with a previously affected offspring, and supports the workflow with the largest to-date retrospective analysis of outcomes.

## Conclusions

Identification of causes through NGS, coupled with prenatal fetal genotyping in subsequent pregnancies, allows families to make informed decisions to reduce recessive disease recurrence.

## Supplementary information

**Additional file 1: Section S1.** Methods. **Section S2.** Abbreviations. **Figure S1.** Flowchart of Phenome-Genome correlation analysis in SimulConsult® DDSS. **Figure S2.** SimulConsult® Summary of clinical features, family history and differential diagnosis (DD) before and after incorporation of patient genetic information. **Figure S3.** Pedigrees and patient features representing each group of clinical diagnosis. **Figure S4.** Distribution of number of clinical features per family from Table S1. **Figure S5.** Power analysis curves. **Table S1.** ACMG rank of variants and SimulConsult Decision Support Software output. **Table S2.** Clinical and imaging findings in 74 families that received prenatal diagnosis. **Table S3.** Detailed information on pathogenic variants, results of prenatal testing and pregnancy outcomes in 86 pregnancies that received amniocentesis. **Table S4.** Detailed phenotype on offspring from families in which fetus genotyped as affected according to prenatal diagnostic testing.

**Additional file 2: Data S1.** SimulConsult® Phenome-Genome output pages on each family qualifying for referral for amniocentesis.

## Data Availability

The datasets used and/or analyzed during the current study are available from the corresponding author on request. Sequence data is available at dbGaP under study ID phs000744 at the following website https://www.ncbi.nlm.nih.gov/gap/?term=phs000744.

## Abbreviations

rPBD: recessive pediatric brain disease
NGS: Next generation sequencing
PND: Prenatal diagnosis
eTOP: elective termination of pregnancy
LCV: Likely causative variant
VUS: Variant of unknown significance
GUS: Gene of unknowns significance

## Abbreviations for diagnostic codes

AEM: Autism Epilepsy Mental retardation
AGS: Aicardi Goutieres Syndrome
ARA: Autosomal Recessive Ataxia
CBA: Cerebellar Atrophy/ Hypoplasia
CCH: Corpus Callosum Hypoplasia
CDG: Congenital Disorder of Glycosylation
CLN: Ceroid Lipofuscinosis, Neuronal
CMD: Congenital Muscular Dystrophy
COACH: Coloboma Oligophrenia Ataxia Cerebellar hypoplasia Hepatic fibrosis
CS: Cockayne Syndrome
CVH: Cerebellar Vermis Hypoplasia
DBD: Degenerative Brain Disease
DMJD: Diencephalic Mesencephalic Junction Dysplasia
EBH: Encephalocele Band Heterotopia
EIEE: Early Infantile Epileptic Encephalopathy
EPI: Epilepsy
EPM: Epilepsy, Progressive Myoclonic
HLD: Hypomyelinating Leukodystrophy
HSP: Hereditary Spastic Paraplegia
HYH: Hydrocephalus
ID: Intellectual Disability
INCL: Infantile Neuronal Ceroid Lipofuscinosis
JBTS: Joubert Syndrome
LIS: Lissencephaly
MCPH: Microcephaly, primary
MIC: Microcephaly
Mito: Mitochondrial disease
MMR: Microcephaly & Mental Retardation
MR/MRT: Mental Retardation
MRE: Mental Retardation & Epilepsy
MSGP: Microcephaly & Simplified Gyral Pattern
MSS: Marinesco Sjogren Syndrome
MTI: Molar Tooth on Imaging
MTDPS5: Mitochondrial DNA Depletion Syndrome
NCL: Neuronal Ceroid Lipofuscinosis
PCH: Ponto-Cerebellar Hypoplasia
PMG: Polymicrogyria
Sd: Syndrome
SPG: Spastic Paraplegia
WMD: White Matter Disease
WWS: Walker Warburg syndrome
