# Supplementary material for: Molecular diagnosis in recessive pediatric neurogenetic disease can help reduce disease recurrence in families
Source: BMC Med Genomics. 2020 May 13;13:68. doi: 10.1186/s12920-020-0714-1 (PMC7218834; doi:10.1186/s12920-020-0714-1)
Supplement: Supplementary file 1 — Additional file 1: Section S1. Methods. Section S2. Abbreviations. Figure S1. Flowchart of Phenome-Genome correlation analysis in SimulConsult® DDSS. Figure S2. SimulConsult® Summary of clinical features, family history and differential diagnosis (DD) before and after incorporation of patient genetic information. Figure S3. Pedigrees and patient features representing each group of clinical diagnosis. Figure S4. Distribution of number of clinical features per family from Table S1. Figure S5. Power analysis curves. Table S1. ACMG rank of variants and SimulConsult Decision Support Software output. Table S2. Clinical and imaging findings in 74 families that received prenatal diagnosis. Table S3. Detailed information on pathogenic variants, results of prenatal testing and pregnancy outcomes in 86 pregnancies that received amniocentesis. Table S4. Detailed phenotype on offspring from families in which fetus genotyped as affected according to prenatal diagnostic testing. [file 12920_2020_714_MOESM1_ESM.docx]

**Supplementary Material**

**Molecular diagnosis in recessive pediatric neurogenetic disease supports reduction of recurrence in families**

Mahmoud Y. Issa^1,2^, Zinayida Chechlacz^1^, Valentina Stanley^1^, Renee D. George^1^, Jennifer McEvoy-Venneri^1^, Denice Belandres^1^, Hasnaa M. Elbendary^2^, Kahled R. Gaber^3^, Ahmad Nabil^4^, Mohamed S. Abdel-Hamid^4^, Joseph G. Gleeson^1^, Maha S. Zaki^2+^,

**Table of contents**

| Section S1 | Methods | p. 2 |
| --- | --- | --- |
| Figure S1 | Flowchart of Phenome-Genome correlation analysis in SimulConsult® DDSS | p. 3 |
| Figure S2 | SimulConsult® Summary of clinical features, family history and differential diagnosis (DD) before and after incorporation of patient genetic information | p. 4 |
| Figure S3 | Pedigrees and patient features representing each group of clinical diagnosis | p. 5 |
| Figure S4 | Distribution of number of clinical features per family from Table S1 | p. 6 |
| Figure S5 | Power analysis curves | p. 7 |
| Table S1 | ACMG rank of variants and SImulConsult® zygosity pertinence | p. 8-15 |
| Table S2 | Clinical and imaging findings in 74 families that received prenatal diagnosis | p. 16-17 |
| Table S3 | Detailed information on pathogenic variants, results of prenatal testing and pregnancy outcomes in 86 pregnancies that received amniocentesis | p. 18-19 |
| Table S4 | Detailed phenotype on offspring from families in which fetus genotyped as affected according to prenatal diagnostic testing. | p. 20 |
| References |  | p. 21 |
| Data S1 | SimulConsult® Phenome-Genome output pages on each family qualifying for referral to amniocentesis. | p. 22-171 |

**Methods**

**Whole exome sequencing and variant prioritization**.

We performed WES in 1172 families with affected(s) displaying features of severe neurodevelopmental disorder consistent with recessive mode of inheritance. Exome sequencing. Variant calling and variant prioritization was performed essentially as described [1] with following modifications:

1) Following GATK variant calling, variants were annotated with Variant Effect Predictor or with in-house software and the SeattleSeq server [2].

2) The variant was identified as rare if allele frequency in gnomAD was less than 0.1% and in the Greater Middle eastern [1] population of less than 0.1%. For known ClinVar variants, no maximum AF threshold was set.

3) For consanguineous families, the variant was required to be present within the Linkage peak as defined by parametric linkage analysis with LOD > 1.8 or in a ‘run of homozygosity’ of at least 1 Mb [3].

4) Compound heterozygous variants were called and prioritized as following/ according to the same requirements for families in which there was no documented history of parental consanguinity. Phase of potential compound heterozygous variants was established by Sanger sequencing the parents, and only instances in which variants were consistent with recessive (i.e. one mutant allele from each parent), segregating in the family as a strict recessive, were prioritized for assessment.

**Reporting genotyping results.**

The results of the research sequencing were provided to the medical team and subsequently to the families after confirmation of the variant with ‘pathogenic’ or a ‘likely pathogenic’ variant (or equivalent in the current ACMG guidelines by the time of variant identification) in a gene that was reported in OMIM in association with a severe pediatric neurologic disease after confirmation of the variant with Sanger sequencing, after confirmation that the variant segregated in the family as a strict recessive trait by genotyping all available healthy and diseased individuals, including genetically informative extended family members. For further assessment of the effect on gene function for VUSs predicted to impact splicing, splicing around the mutation was assessed in primary dermal fibroblasts in willing families, using primers to amplify cDNA, followed by Sanger sequencing. Combining this information with variant interpretation led to classification of variants within ACMG guidelines.

**SimulConsult**® **(SC) variant validation**

SC Genome-Phenome Analyzer was used in this study for the following reasons: 1) it provided a mechanism for hypothesis-independent confirmation of the molecular cause, free from assumptions about mode of inheritance, number of genes involved, or which clinical findings were most relevant [4], 2) is was the only software that ranked the probability for the variant to be disease-causing and the probability for the disease to correspond to the correct patient diagnosis, 3) it contains one of the largest datasets for clinical features of severe inherited conditions (in particular pediatric genetics and neurology) with more than 6500 diagnoses and over 3600 genes, 4) it was among the 8 best performing finalists in the CLARITY study [5] and 5) has been successfully implicated previously in a similar cohort of patients [4]. Relevant information regarding family and disease history, physical and neurological exam and diagnostic laboratory data (i.e. imaging, metabolic, electrophysiological tests) and medical specialist consultations (i.e. ophthalmologic and cardiologic) were assembled within the SC interface to generate a differential diagnosis (Figure S1 and Figure S2).  Subsequently, the entire annotated variant table, typically consisting of tens of thousands of variants and their zygosities, was collated into SC, which then assigned a severity score to each variants based upon effect scores (e.g., missense versus frameshift), functional and conservation damage prediction score and allele frequency using a clinically validated scale from 1 (benign) to 5 (most severe) and then determined the allele zygosities that were plausible from the variant table, including those for monoallelic (dominant) and biallelic (recessive, including homozygous or compound heterozygous) conditions.  Computation of a “pertinence” metric for each gene zygosity, representing the joint probability for the clinical features and the genetic severity score for each known OMIM disease, provided a quantitative measurement of the ‘goodness of fit’ between the candidate zygosities and the presenting features [4].

**Supplementary Figure 1. Flowchart of Phenome-Genome correlation in SimulConsult**®  **DDSS**

Clinical information on the patient with family history in line with a probable recessive Mendelian disorder was first evaluated to generate a list of differential diagnoses (DD) (Step 1 and Step 2). Patient sequencing data was mapped and filtered to generate a vcf table incorporating variant pathogenicity and conservation scores along with their zygosities (Step 3). These tables were used by SimulConsult® software to assign severity scores to each variant (Step 4) aligned with the DD that were generated in Step 2, thus providing a calculated joint probability between signs/symptoms and genetic mutation match (Pertinence). This combinatorial approach led to the identification of a single variant with the highest pertinence (likelihood to be disease-causing in this patient) and of the precise genetic diagnosis (Step 5). The pertinence of the initial clinical symptoms was recalculated according to the established diagnosis (Step 6) and is shown in summary tables after incorporation of the genetic information for each patient.

**Supplementary Figure 2. SimulConsult**® **summary of clinical features, family history and differential diagnosis (DD) before and after incorporation of patient genetic information**

Clinical findings and family history without patient genetic information (Step 1, upper left) resulted in output of likely clinical diagnoses with the respective probability for each (Step 2, bottom left). Incorporation of the patient genetic variants with respective conservation and pathogenicity scores (calculated together as variant severity in SC, Step 4) allowed for the calculation of the variant pertinence metric that represents the strength of the correlation between genetic variant and the phenotypic presentation. As a result, only LIS2 that was initially ranked 5^th^ on the DD list, pertained the highest joint probability for the signs and symptoms.

***
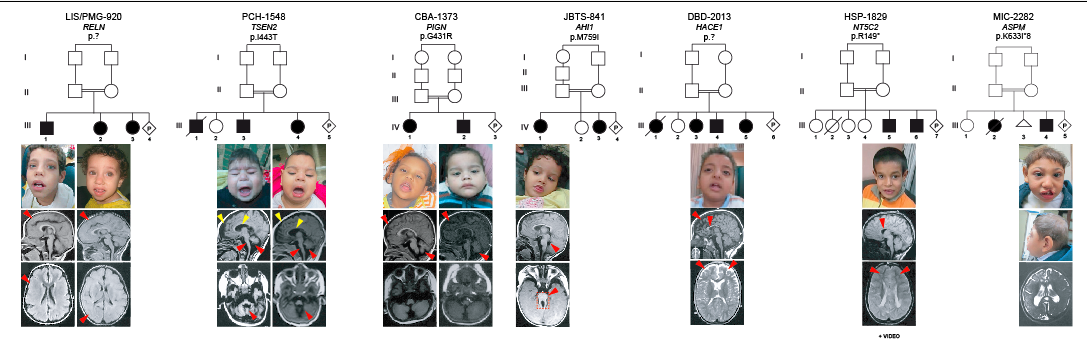
*Supplementary Figure 3. Pedigrees and clinical features representing each group of clinical diagnoses.**

Example families with a clinical diagnosis, gene with a biallelic pathogenic variant and predicted effect on protein. Each family here presented with 2 or more affected children with nearly identical disease (filled symbols). Older affected siblings were previously deceased in families 1548, 2013 and 2282 (diagonal line). Subsequent pregnancy indicated with diamond and P. Below shows facial images and brain scans of patients for each family. Patients from family LIS/PMG-920 (lissencephaly/polymicrogyria) with a pathogenic variant in *RELN* showed a broad nasal bridge, a sloping forehead. Brain MRI showed a simplification of cerebral cortical gyral pattern with a thickened cerebral cortical mantle (red arrowheads) on midline sagittal and axial brain MRI. Patients from family PCH-1548 (pontocerebellar hypoplasia) with a pathogenic variant in *TSEN2* showed a sloping forehead and a long philtrum. Brain MRI showed brainstem pontine and cerebellar hemispheric hypoplasia (red arrowheads), along with cerebral cortical atrophy and thin corpus callosum (yellow arrowheads). Patients from family CBA-1373 (cerebellar atrophy) with pathogenic variant in *PIGN* presented with dysmorphic low-set ears, and epicanthal folds. Brain MRI showed cerebellar hypoplasia and cerebral cortical atrophy (red arrowheads). Patient from family JBST-841 (Joubert syndrome) with a pathogenic variant in *AHI1* presented with anteverted nostrils, tented upper lip with protruding tongue, and high arched eyebrows. Brain MRI showed deep posterior interpeduncular fossa and thickened and elongated superior cerebellar peduncles characteristic of the “molar tooth” sign. Patients from family DBD-2013 (degenerative brain disease) with a pathogenic variant in *HACE1* showed a broad forehead and hypertelorism. Brain MRI showed cerebral cortical atrophy and hypoplastic corpus callosum (red arrowheads). Patients from family HSP-1829 (hereditary spastic paraplegia) with a pathogenic variant in *NT5C2* gene showed no notable dysmorphic features but brain MRI showed corpus callosum hypogenesis and diffuse white matter abnormalities (red arrowheads). Patients from family MIC- 2282 (microcephaly) with a pathogenic variant in *ASPM* showed cerebral microcephaly, coarsened facial features, prominent eyes and arched eyebrows, short sloping forehead, lip cleft and large ears. Brain MRI showed simplified cerebral cortical gyral pattern.

***
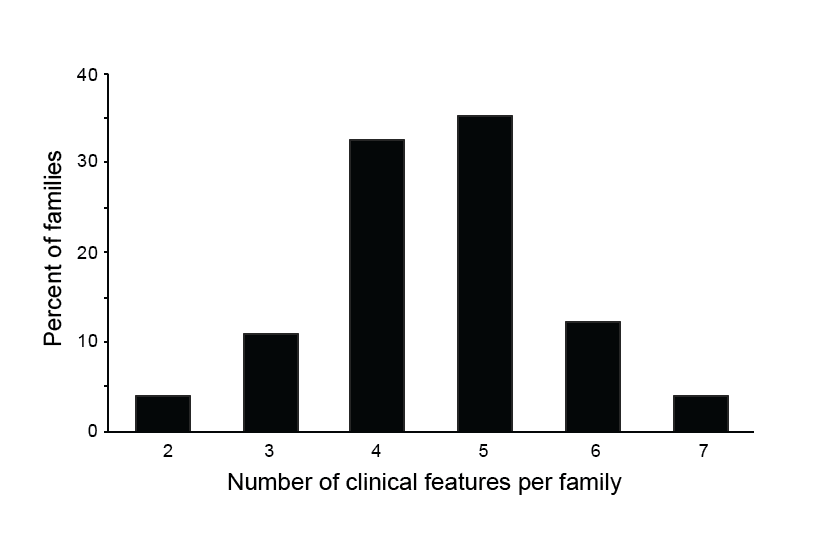
***

**Supplementary Figure 4. Distribution of number of clinical features per family from Table S1.** *X-*axis lists the number of clinical features (pathogenic signs and symptoms) observed in the affected members of each family. Clinical features include: epilepsy, microcephaly, brain atrophy, spasticity, motor delay, intellectual disability, ataxia and dysmorphism. *Y-*axis lists the percent of families within each group. A majority of families presented with 4 to 5 assessed clinical feature.


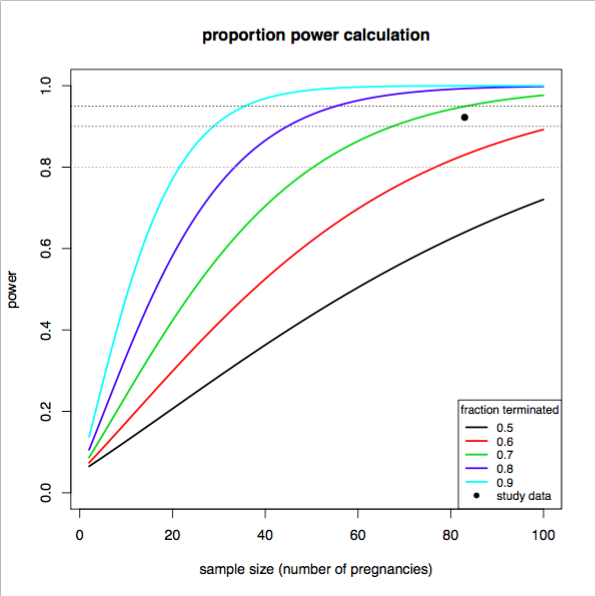


**Supplementary Figure 5. Power analysis curves.** Power to detect significance in the fraction of affected births, compared to 25% expected affected pregnancies in the absence of prenatal testing. Power was calculated as a function of sample size and effect size was calculated from the fraction of predicted affected pregnancies terminated. A lower ‘fraction terminated’ resulted in a smaller effect size. We used a two-tailed proportion test (R function pwr.p.test), with sample size equal to the number of pregnancies minus the number terminated, defined by 25% predicted affected, and fraction terminated. The study data of 67% predicted affected terminated and 83 pregnancies results in a power of 92.7%.

7

|  | **ID #** | **Presumptive Diagnosis** | **Gene** | **Variant Type** | **Variant position (hg19)** | **ACMG Classification** | **ACMG evidence** | **NM Transcript and cDNA position** | **Protein** | **Simul Consult® Variant Severity** | **Simul Consult® Zygosity Pertinence** |
| --- | --- | --- | --- | --- | --- | --- | --- | --- | --- | --- | --- |
| 1 | 520 | EBH | LAMB1 | indel fs* | 7:107592590delinsGACCCAGTGCTTGTGTCTTCCTAATGTGCTTGTGTCTTCCTAAT | Pathogenic | PVS1, PM4, PP1, PP4 | NM_002291.2:c.3141_3158delinsGACCCAGTGCTTGTGTCTTCCTAATGTGCTTGTGTCTTCCTAAT | NP_002282.2: p.K1049fsX1056 | 5 | >95% |
| 2 | 702 | MMR | VPS13B | missense/ splice | 8:100589861G>T | Pathogenic | PVS1, PP1-M, PM2 | NM_017890.4:c.5295G>T | NP_060360.3:p.Glu1765Asp | 5 | >95% |
| 3 | 711 | LIS-PMG | KATNB1 | missense | 16:57789805T>G | Likely pathogenic | PM2, PS4-M, PP1, PP5 | NM_005886.2:c.1619T>G | NP_005877.2:p.Leu540Arg | 5 | >95% |
| 4 | 718 | MIC | CIT | missense | 12:120295424C>A | Likely pathogenic | PS3, PM2, PP1, PP3 | NM_001206999.1:c.317G>T | NP_001193928.1:p.Gly106Val | 5 | >95% |
| 5 | 819 | PCH | TSEN54 | missense | 17:73513639G>T | Likely pathogenic | PM2, PP1-M, PP3, PP5, | NM_207346.2:c.371G>T | NP_997229.2:p.Gly124Val | 5 | >95% |
| 6 | 841 | MTI | AHI1 | missense | 6:135752442C>T | Likely pathogenic | PM2, PP1-M, PS4-M, PP3 | NM_001134830.1:c.2277G>A | NP_060121.3:p.Met759Ile | 5 | >95% |
| 7 | 920 | LIS | RELN | splice | 7:103207046A>C | Pathogenic | PVS1, PM2, PP1 | NM_005045.3:c.4747+2T>G | NP_005036.2 | 5 | >95% |
| 8 | 923 | PCH | TSEN54 | missense | 17:73518081G>T | Likely pathogenic | PS1, PP1, PP5 | NM_207346.2:c.919G>T | NP_997229.2:p.Ala307Ser | 5 | >95% |
| 9 | 997 | MTI | TMEM67 | nonsense/ splice | 8:94767192T>A, 8:94817111G>C | Pathogenic /Likely pathogenic | PVS1, PM2, PP1/ PM2, PM3, PP1, PP3 | NM_153704.5:c.50T>A/NM_153704.5:c.2439+5G>C | NP_714915.3:p.Leu17Ter/ N/A | 4C | >95% |
| 10 | 1004 | MIC | GJC2 | missense | 1:228345562C>T | Likely pathogenic | PM1, PM2, PP1, PP3 | NM_020435.3:c.103C>T | NP_065168.2:p.Arg35Cys | 5 | >95% |
| 11 | 1010 | MTI | AHI1 | del fs* | 6:135778720GTCTA>G | Pathogenic | PVS1, PM2, PP1 | NM_017651.4:c.1059_1062del | NP_060121.3:p.Arg354LeufsTer6 | 5 | >95% |
| 12 | 1190 | MTI | TMEM138 | del fs* | 11:61135400AC>A | Pathogenic | PVS1, PM2, PP1 | NM_016464.4:c.307del | NP_057548.1:p.Leu102PhefsTer24 | 5 | >95% |
| 13 | 1261 | PMG | ADGRG1 | ins fs* | 16:57689383A>AG | Pathogenic | PVS1, PM2, PP1 | NM_005682.6:c.842dup | NP_005673.3:p.Ser281ArgfsTer5 | 5 | >95% |
| 14 | 1269 | MIC | ZNF335 | missense | 20:44590754T>C | Likely pathogenic | PM2, PP1-S, PP3 | NM_022095.3:c.1601A>G | NP_071378.1:p.Tyr534Cys | 5 | >95% |
| 15 | 1273 | MIC | POMT2 | missense | 14:77746411A>G | Likely pathogenic | PP1-S, PM2, PP3 | NM_013382.5:c.1738T>C | NP_037514.2:p.Ser580Pro | 5 | >95% |
| 16 | 1368 | COACH | TMEM237 | del fs* | 2:202498140AG>A | Pathogenic | PVS1, PP1-S | NM_001044385.2:c.288del | NP_001037850.1:p.Ser97ProfsTer49 | 4 | >95% |
| 17 | 1373 | CBA | PIGN | missense | 18:59780510C>T | Likely pathogenic | PP1-S, PM2, PP3 | NM_012327.5:c.1291G>A | NP_036459.1:p.Gly431Arg | 5 | >95% |
| 18 | 1381 | WWS | POMGNT1 | del fs* | 1:46658038ACCCAG>A | Pathogenic | PVS1, PM2, PM4 | NM_001243766.1:c.1350_1354del | NP_001230695.1:p.Trp451AlafsTer11 | 5 | >95% |
| 19 | 1391 | PCH | AMPD2 | missense | 1:110173362G>T | Likely pathogenic | PM2, PM5, PP3, PP5 | NM_001257360.1:c.2377G>T | NP_001244289.1:p.Asp793Tyr | 5 | >95% |
| 20 | 1393 | HSP | ALS2 | missense | 2:202591271C>T | Likely pathogenic | PP1-S, PM2, PP3 | NM_020919.3:c.3184G>A | NP_065970.2:p.Gly1062Arg | 5 | >95% |
| 21 | 1406 | MRE | KCTD7 | missense | 7:66104184C>T | Likely pathogenic | PP1-S, PM2, PP2 | NM_153033.4:c.835C>T | NP_694578.1:p.Arg279Cys | 5 | >95% |
| 22 | 1524 | MTI | C5ORF42 | ins fs*/ missense | 5:37231049C>CAA, 5:37213735T>C | Pathogenic/Likely pathogenic | PVS1, PP1-S, PM2/PP1-S, PM2, PM3, PP3 | NM_023073.3:c.1039_1040dup/NM_023073.3:c.2846A>G | NP_075561.3:p.Leu347PhefsTer3/NP_075561.3:p.Tyr949Cys | 5C | >95% |
| 23 | 1548 | PCH | TSEN2 | missense | 3:12573148T>C | Likely pathogenic | PP1-S, PM2, PP3 | NM_001321278.1:c.1328T>C | NP_001308207.1:p.Ile443Thr | 5 | >95% |
| 24 | 1588 | CBA | FKTN | missense | 9:108382302T>C | Likely pathogenic | PP1-S, PM2, PP3 | NM_006731.2:c.1132T>C | NP_006722.2:p.Trp378Arg | 5 | >95% |
| 25 | 1592 | DMJD | PCDH12 | del fs* | 5:141334905TC>T | Pathogenic | PVS1, PM2, PP1, PP5 | NM_016580.3:c.2511del | NP_057664.1:p.Ser838AlafsTer26 | 5 | >95% |
| 26 | 1644 | MR-MIC | VPS13B | del fs* | 8:100779134TTA>T | Pathogenic | PVS1, PM2, PP1 | NM_017890.4:c.7259_7260del | NP_060360.3:p.Leu2420CysfsTer18 | 5 | >95% |
| 27 | 1702 | ARA | ALDH5A1 | ins fs* | 6:24528264A>AC | Pathogenic | PVS1, PM2, PP1 | NM_170740.1:c.1254dup | NP_733936.1:p.Val419ArgfsTer16 | 5 | >95% |
| 28 | 1713 | MR | CRBN | missense | 3:3194228C>T | Likely pathogenic | PP1-S, PM2, PP3 | NM_016302.3:c.1060G>A | NP_057386.2:p.Gly354Arg | 5 | >95% |
| 29 | 1743 | NCL | PPT1 | nonsense | 1:40539819G>A | Pathogenic | PVS1, PM2, PP5 | NM_000310.3:c.835C>T | NP_000301.1:p.Gln279Ter | 5 | >95% |
| 30 | 1829 | HSP | NT5C2 | nonsense | 10:104861028T>A | Pathogenic | PVS1, PM2, PP1 | NM_012229.4:c.445A>T | NP_036361.1:p.Arg149Ter | 5 | >95% |
| 31 | 1894 | MIC-CVH | ANK3 | del fs* | 10:61829656CTG>C | Pathogenic | PVS1, PP1-S, PM2 | NM_020987.4:c.10981_10982del | NP_066267.2:p.Gln3661ValfsTer22 | 5 | >95% |
| 32 | 2006 | MIC-WMD | RNASEH2C | missense | 11:65487533G>A | Pathogenic | PS1, PP1-S, PM2, PP2, PP3, PP5 | NM_032193.3:c.451C>T | NP_115569.2:p.Pro151Ser | 5 | >95% |
| 33 | 2007 | MTI | ARMC9 | splice | 2:232071007G>T | Likely pathogenic | PM2, PP1-M, PP3, PP5 | NM_001271466.3:c.51+5G>T | NP_079415.3 | 3 | >95% |
| 34 | 2013 | MRE | HACE1 | splice | 6:105198348C>T | Pathogenic | PVS1, PM2, PP3 | NM_020771.3:c.2212-1G>A | NP_065822.2 | 5 | >95% |
| 35 | 2020 | HYH | CENPJ | nonsense | 13:25487031G>A | Pathogenic | PVS1, PM2, PP5 | NM_018451.4:c.133C>T | NP_060921.3:p.Arg45Ter | 5 | >95% |
| 36 | 2022 | MTI | AHI1 | nonsense | 6:135768160G>A | Pathogenic | PVS1, PM2, PP5 | NM_001134830.1:c.1765C>T | NP_060121.3:p.Arg589Ter | 5 | >95% |
| 37 | 2027 | MIC-CBA | WDR73 | missense | 15:85188983C>T | Likely pathogenic | PM2, PP1-M, PS4-M, PP5 | NM_032856.3:c.602G>A | NP_116245.2:p.Gly201Glu | 5 | >95% |
| 38 | 2028 | MSGP | ASPM | nonsense | 1:197062286G>A | Pathogenic | PVS1, PM2, PP5 | NM_018136.4:c.9190C>T | NP_060606.3:p.Arg3064Ter | 5 | >95% |
| 39 | 2056 | CDG | SRD5A3 | nonsense | 4:56225611G>A | Pathogenic | PVS1, PM2, PP1, PP5 | NM_024592.4:c.320G>A | NP_078868.1:p.Trp107Ter | 5 | >95% |
| 40 | 2131 | PMG | WDR62 | missense | 19:36577615C>T | Likely pathogenic | PP1-S, PM2, PP3 | NM_001083961.1:c.1669C>T | NP_001077430.1:p.Arg557Trp | 4 | >95% |
| 41 | 2132 | HSP | RNASEH2B | missense | 13:51519581G>A | Likely pathogenic | PS3, PP2, PP3, PP5 | NM_024570.3:c.529G>A | NP_078846.2:p.Ala177Thr | 5 | >95% |
| 42 | 2236 | Martsolf | RAB3GAP1 | splice | 2:135883820G>C | Pathogenic | PVS1, PM2, PP3 | NM_001172435.1:c.899+1G>C | NP_036365.1 | 5 | >95% |
| 43 | 2245 | PMG | ADGRG1 | nonsense | 16:57685282C>T | Pathogenic | PVS1, PM2, PP5 | NM_005682.6:c.235C>T | NP_005673.3:p.Arg79Ter | 5 | >95% |
| 44 | 2247 | CS | ERCC8 | nonsense | 5:60214191G>C | Pathogenic | PVS1, PM2, PP5 | NM_000082.3:c.300C>G | NP_000073.1:p.Tyr100Ter | 5 | >95% |
| 45 | 2248 | CCH | MCOLN1 | del fs* | 19:7590039TG>T | Pathogenic | PVS1, PM2, PP1 | NM_020533.2:c.226del | NP_065394.1:p.Val76SerfsTer16 | 5 | >95% |
| 46 | 2282 | MIC | ASPM | del fs* | 1:197111484TTC>T | Pathogenic | PVS1, PP1-S, PM2 | NM_018136.4:c.1896_1897del | NP_060606.3:p.Lys633IlefsTer8 | 5 | >95% |
| 47 | 2303 | Martsolf | RAB3GAP1 | nonsense | 2:135893304G>A | Pathogenic | PVS1, PM2, PP1 | NM_001172435.1:c.1725G>A | NP_001165906.1:p.Trp575Ter | 5 | >95% |
| 48 | 2421 | MSS | TBC1D20 | nonsense | 20:428590G>A | Pathogenic | PVS1, PM2, PP1 | NM_144628.3:c.199C>T | NP_653229.1:p.Arg67Ter | 5 | >95% |
| 49 | 2424 | MIC | ST3GAL5 | missense | 2:86067500C>T | Likely pathogenic | PP1-S, PM2, PP3 | NM_003896.3:c.1024G>A | NP_003887.3:p.Gly342Ser | 5 | >95% |
| 50 | 2450 | CMD | GMPPB | missense | 3:49760037G>A | Likely pathogenic | PP1-M, PM2, PP2, PP3, PP5 | NM_013334.3:c.553C>T | NP_037466.2:p.Arg185Cys | 4 | >95% |
| 51 | 2566 | MIC | PYCR2 | nonsense | 1:226108909G>A | Pathogenic | PVS1, PM2, PP5 | NM_013328.3:c.796C>T | NP_037460.2:p.Arg266Ter | 5 | >95% |
| 52 | 2641 | MTI | TMEM138 | missense | 11:61135470G>A | Likely pathogenic | PP1-M, PM2, PP2, PP3, PP5 | NM_016464.5:c.376G>A | NP_057548.1:p.Ala126Thr | 4 | >95% |
| 53 | 2643 | MIC | SLC1A4 | ins fs* | 2:65243654T>TG | Pathogenic | PVS1, PM2, PP1 | NM_003038.4:c.885dup | NP_003029.2:p.Lys296GlufsTer62 | 5 | >95% |
| 54 | 2664 | MIC | PYCR2 | nonsense | 1:226108909G>A | Pathogenic | PVS1, PM2, PP5 | NM_013328.3:c.796C>T | NP_037460.2:p.Arg266Ter | 5 | >95% |
| 55 | 2715 | PMG | POMGNT1 | missense | 1:46657796C>T | Likely pathogenic | PM1, PM2, PP1, PP3 | NM_001243766.1:c.1513G>A | NP_001230695.1:p.Gly505Ser | 5 | >95% |
| 56 | 2730 | DBD | CLN2 | ins fs*/ missense | 11:6636216T>TAG, 11:6636204C>T | Pathogenic/Pathogenic | PVS1, PM2, PP1/PS1, PM1, PM2, PM3, PP1, PP2, PP5 | NM_000391.3:c.1430_1431dup/NM_000391.3:c.1444G>A | NP_000382.3:p.Thr478LeufsTer11/NP_000382.3:p.Gly482Arg | 5C | >95% |
| 57 | 2761 | AEM | PIGT | nonsense/ missense | 20:44047543C>A, 20:44047955C>T | Pathogenic/Likely pathogenic | PVS1, PM2, PP1/PM2, PM3, PP1, PP3 | NM_015937.5:c.417C>A/NM_015937.5:c.514C>T | NP_057021.2:p.Cys139Ter/NP_057021.2:p.Arg172Cys | 5C | >95% |
| 58 | 2887 | HSP | ALS2 | missense | 2:202626188 C>A | Likely pathogenic | PP1-S, PM2, PP3 | NM_020919.3:c.529G>T | NP_065970.2:p.Gly177Cys | 5 | >95% |
| 59 | 2889 | MIC-CBA | WDR73 | missense | 15:85188983C>T | Likely pathogenic | PM2, PP1-M, PS4-M, PP5 | NM_032856.3:c.602G>A | NP_116245.2:p.Gly201Glu | 5 | >95% |
| 60 | 2904 | NCL | KCTD7 | missense | 7:66104041C>T | Likely pathogenic | PM2, PP1-M, PP2, PP3 | NM_153033.4:c.692C>T | NP_694578.1:p.Ser231Phe | 5 | >95% |
| 61 | 3064 | MR | LINS1 | del fs* | 15:101115275TCA>T | Pathogenic | PVS1, PM2, PP1 | NM_001040616.2:c.546_547del | NP_001035706.1:p.Ser184Ter | 4 | >95% |
| 62 | 3127 | MIC | AP4M1 | ins fs* | 7:99703627G>GT | Likely pathogenic | PVS1, PM2 | NM_004722.3:c.974+2dup | NP_004713.2 | 4 | >95% |
| 63 | 3130 | MIC | UBE3B | missense | 12:109959356G>C | Likely pathogenic | PP1-S, PM2, PP3 | NM_130466.3:c.2364G>C | NP_569733.2:p.Glu788Asp | 5 | >95% |
| 64 | 3158 | EIEE | MOCS2 | missense (Met1) | 5:52405557C>T | Pathogenic | PVS1, PM2, PP1 | NM_004531.4:c.-646G>A | NP_904327.1 | 5 | >95% |
| 65 | 3163 | CBA-MIC | SIL1 | missense | 5:138282969C>G | Likely pathogenic | PP1-M, PM2, PP2, PP3, PP4 | NM_022464.5:c.1223G>C | NP_071909.1:p.Cys408Ser | 5 | >95% |
| 66 | 3172 | MIC | PARS2 | missense | 1:55223696C>A | Likely pathogenic | PM2, PP1-S, PP3 | NM_152268.3:c.1139G>T | NP_689481.2:p.Gly380Val | 5 | >95% |
| 67 | 3191 | MIC-HYH | ASPM | del fs* | 1:197112693 TC>T | Pathogenic | PVS1, PM2, PP1 | NM_018136.4:c.688del | NP_060606.3:p.Glu230AsnfsTer30 | 4 | >95% |
| 68 | 3200 | MIC-CCH | AP4M1 | del fs* | 7:99702686CG>C | Pathogenic | PVS1, PM2, PP1 | NM_004722.3:c.694del | NP_004713.2:p.Glu232LysfsTer10 | 4 | >95% |
| 69 | 3208 | MSGP | PHGDH | missense | 1:120285493G>A | Likely pathogenic | PP1-M, PM2, PP2, PP3, PP5 | NM_006623.3:c.1273G>A | NP_006614.2:p.Val425Met | 5 | >95% |
| 70 | 3226 | CBA | PLA2G6 | splice | 22:38525460C>T | Pathogenic | PVS1, PM2, PP1 | NM_003560.3:c.1186+1G>A | NP_003551.2 | 5 | >95% |
| 71 | 3310 | DBD | GALC | missense | 14:88416259A>G | Likely pathogenic | PP1-S, PM2, PP3 | NM_000153.3:c.1268T>C | NP_000144.2:p.Leu423Pro | 5 | >95% |
| 72 | 3356 | DBD-NCL | PPT1 | nonsense | 1:40558158A>C | Pathogenic | PVS1, PM2, PP1 | NM_000310.3:c.146T>G | NP_000301.1:p.Leu49Ter | 5 | >95% |
| 73 | 3818 | WMD | PCDH12 | del fs* | 5:141334650 CAG>C | Pathogenic | PVS1, PM2, PP1 | NM_016580.3:c.2765_2766del | NP_057664.1:p.Pro922ArgfsTer62 | 4 | >95% |
| 74 | 4101 | Mito | SUCLA2 | missense | 13:48563018A>G | Likely pathogenic | PP1-S, PM2, PP3 | NM_003850.2:c.370T>C | NP_003841.1:p.Ser124Pro | 5 | >95% |

**Supplementary Table 1. SimulConsult (SC) validation of variant pertinence for each diagnosis in 74 families seeking prenatal genetic counseling.** Family ID, presumptive diagnosis (see abbreviations), gene mutated, type of mutation, hg19 annotation, ACMG classification and evidence, NM Transcript, cDNA position, SimulConsult® Variant Severity and SimulConsult® Zygosity Pertinence. Variant severity score was assigned independently of phenotype and incorporated a pathogenicity scores of 1 to 5 (1:benign, 5: loss-of-function). C: compound heterozygous. Zygosity Pertinence, ranging from 0-100%, provides a joint probability of a match between signs/symptoms and gene mutation. None of the comparisons resulted in exclusion of a family from this study. Abbreviations: see Section S2 in the Supplement. PM, pathogenic moderate; PP, pathogenic supporting; PS, pathogenic strong; PVS, pathogenic very strong.

| # | **Study#** | **Clinical Diagnosis** | **Gene** | **Genetic Diagnosis (SC/OMIM)** | **Disease group** | **EPI** | **Motor delay** | **ID** | **CBA or Ataxia** | **Spasticity** | **Regression** | **MIC or Atrophy** | **Dys**  **morphic** | **ID Score** |
| --- | --- | --- | --- | --- | --- | --- | --- | --- | --- | --- | --- | --- | --- | --- |
| 1 | 520 | EBH | LAMB1 | LIS5 | LIS/PMG | y | y | y | y | n.a. | y | y | y | profound |
| 2 | 702 | MMR | VPS13B | Cohen Syndrome | MIC | n | y | y | n | n | n | y | y | severe |
| 3 | 711 | LIS-PMG | KATNB1 | LIS6 | LIS/PMG | y | y | y | n | n | n | y | y | severe |
| 4 | 718 | MIC | CIT | MCPH17 | MIC | n | y | y | n | y | n | y | y | severe |
| 5 | 819 | PCH | TSEN54 | PCH2 | PCH | y | y | y | y | n.a. | n.a. | y | n | profound |
| 6 | 841 | MTI | AHI1 | JBTS3 | MTI | n | y | y | y | n.a. | n | n | y | profound |
| 7 | 920 | LIS | RELN | LIS2 | LIS/PMG | n | y | y | n | n.a. | n | y | n | severe |
| 8 | 923 | PCH | TSEN54 | PCH2 | PCH | y | y | y | y | y | n.a. | y | n | profound |
| 9 | 997 | MTI | TMEM67 | JBTS6 | MTI | y | y | y | y | n | n | n | y | profound |
| 10 | 1004 | MIC | GJC2 | HLD2-Pelizaeus-Merzbacher-Like | MIC | n | y | y | y | y | n | y | n | severe |
| 11 | 1010 | MTI | AHI1 | JBTS3 | MTI | n | y | y | y | n | n.a. | n | y | profound |
| 12 | 1190 | MTI | TMEM138 | JBTS16 | MTI | n | y | y | y | n | n | n | y | profound |
| 13 | 1261 | PMG | GPR56 | PMG | LIS/PMG | y | y | y | y | y | n | n | y | severe |
| 14 | 1269 | MIC | ZNF335 | MCPH10 | MIC | y | y | y | n | y | n | y | y | severe |
| 15 | 1273 | MIC | POMT2 | Muscular dystrophy-dystroglycanopathy B2 | MIC | n | y | y | n | n | n | y | y | profound |
| 16 | 1368 | COACH | TMEM237 | JBTS14 | MTI | y | y | y | y | n | n | n | y | profound |
| 17 | 1373 | CBA | PIGN | Multiple congen anom hypot seiz | CBA | y | y | y | y | n | n | y | y | profound |
| 18 | 1381 | WWS | POMGNT1 | Muscular dystrophy-dystroglycanopathy A3 | LIS/PMG | n | y | y | y | n | n | n.a. | y | profound |
| 19 | 1391 | PCH | AMPD2 | PCH9 | PCH | y | y | y | y | y | n | y | y | profound |
| 20 | 1393 | HSP | ALS2 | Juvenile primary lateral sclerosis | HSP | n | y | n.a. | n | y | n.a. | n | n | n.a. |
| 21 | 1406 | MRE | KCTD7 | EPM3 | DBD | y | y | y | n.a. | n | y | y | n | severe |
| 22 | 1524 | MTI | C5ORF42 | JBTS17 | MTI | n | y | y | y | n | n | y | y | severe |
| 23 | 1548 | PCH | TSEN2 | PCH2 | PCH | n | y | y | y | y | n | y | y | profound |
| 24 | 1588 | CBA | FKTN | Muscular dystrophy-dystroglycanopathy A4 | CBA | n | y | y | y | n | n | y | y | severe |
| 25 | 1592 | DMJD | PCDH12 | MIC, seizures, spasticity, and brain calcification | MIC | y | y | y | n | y | n | y | y | profound |
| 26 | 1644 | MR-MIC | VPS13B | Cohen Sd | MIC | n | y | y | n | n | n | y | y | severe |
| 27 | 1702 | ARA | ALDH5A1 | Succinic semialdehyde dehydrogenase deficiency | CBA | n | y | y | y | y | n.a. | n | y | severe |
| 28 | 1713 | MR | CRBN | MR | DBD | n | y | y | n | n | n | n | n | severe |
| 29 | 1743 | NCL | PPT1 | INCL (CLN1) | DBD | y | y | y | n | y | y | y | n | profound |
| 30 | 1829 | HSP | NT5C2 | SPG45 | HSP | n | y | y | n | y | n | n | n | moderate |
| 31 | 1894 | MIC-CVH | ANK3 | MRT37 | MIC | n | y | y | y | n | n | y | y | severe |
| 32 | 2006 | MIC-WMD | RNASEH2C | AGS | MIC | y | y | y | y | y | n.a. | y | y | profound |
| 33 | 2007 | MTI | ARMC9 | JBTS30 | MTI | n | y | y | y | n | n | n | y | severe |
| 34 | 2013 | MRE | HACE1 | Spastic paraplegia and psychomotor retardation | DBD | y | y | y | n | n.a. | y | n | y | profound |
| 35 | 2020 | HYH | CENPJ | Seckel Sd | LIS/PMG | n | n.a. | y | n.a. | n.a. | n.a. | y | n.a. | severe |
| 36 | 2022 | MTI | AHI1 | JBTS3 | MTI | n | y | y | y | n | n | n | y | severe |
| 37 | 2027 | MIC-CBA | WDR73 | Galloway-Mowat Sd | CBA | n | y | y | y | n.a. | n | y | y | severe |
| 38 | 2028 | MSGP | ASPM | MCPH5 | MIC | n | y | y | n | n | n | y | y | severe |
| 39 | 2056 | CDG | SRD5A3 | CDG1Q | LIS/PMG | n | y | y | y | n.a. | n | n | y | severe |
| 40 | 2131 | PMG | WDR62 | MCPH2 | LIS/PMG | n | y | y | n | y | n | y | y | severe |
| 41 | 2132 | HSP | RNASEH2B | AGS | LIS/PMG | n | y | n.a. | n | y | y | n | n | n.a. |
| 42 | 2236 | Martsolf | RAB3GAP1 | Warburg/Martsolf | MIC | n.a. | y | y | n.a. | n.a. | n.a. | y | y | severe |
| 43 | 2245 | PMG | GPR56 | PMG | LIS/PMG | n.a. | y | y | n.a. | n.a. | n.a. | y | n.a. | severe |
| 44 | 2247 | CS | ERCC8 | Cockayne Sd | MIC | n | y | y | n | n | n | y | y | severe |
| 45 | 2248 | CCH | MCOLN1 | Mucolipidosis | HSP | n | y | y? | n | y | n.a. | n.a. | y | severe |
| 46 | 2282 | MIC | ASPM | MCPH5 | MIC | n | y | y | n | n.a. | n | y | y | profound |
| 47 | 2303 | Martsolf | RAB3GAP1 | Warburg/Micro | MIC | n | y | y | n | y | n | y | y | severe |
| 48 | 2421 | MSS | TBC1D20 | Warburg/Micro | MIC | n | y | y | y | n | n | y | y | severe |
| 49 | 2424 | MIC | ST3GAL5 | Salt and Pepper Developmental Regression Sd | MIC | y | y | y | n | n | n | y | y | severe |
| 50 | 2450 | CMD | GMPPB | Muscular dystrophy-dystroglycanopathy A14 | MIC | n | y | y | n | n | n | y | y | severe |
| 51 | 2566 | MIC | PYCR2 | HLD10 | MIC | n | y | y | n | n | n | y | y | severe |
| 52 | 2641 | MTI | TMEM138 | JBTS16 | MTI | n | y | y | y | n | n | n | y | severe |
| 53 | 2643 | MIC | SLC1A4 | Spastic tetraplegia, CCH, and progressive MIC | MIC | n | y | y | n | y | n | y | y | severe |
| 54 | 2664 | MIC | PYCR2 | HLD10 | MIC | n | y | y | n | n | n | y | y | severe |
| 55 | 2715 | PMG | POMGNT1 | Muscular dystrophy-dystroglycanopathy A3 | LIS/PMG | y | y | y | n.a. | n.a. | n | y | n.a. | severe |
| 56 | 2730 | DBD | TPP1=CLN2 | CLN2 | DBD | n | y | y | y | n | y | y | n | severe |
| 57 | 2761 | AEM | PIGT | Multiple congenital anomalies-hypotonia-seizures 3 | DBD | y | y | y | n | n | y | n | n | severe |
| 58 | 2887 | HSP | ALS2 | Juvenile primary lateral sclerosis | HSP | y | y | n.a. | n.a. | y | n | n.a. | n.a. | n.a. |
| 59 | 2889 | MIC-CBA | WDR73 | Galloway-Mowat Sd | CBA | n | y | y | y | n | n | y | y | severe |
| 60 | 2904 | NCL | KCTD7 | EPM3 | DBD | y | y | y | n | n | y | y | n | severe |
| 61 | 3064 | MR | LINS1 | MRT27 | DBD | n | y | y | n | n | n | n | y | severe |
| 62 | 3127 | MIC | AP4M1 | SPG50 | MIC | y | y | y | n | y | n | y | y | severe |
| 63 | 3130 | MIC | UBE3B | Kaufman oculocerebrofacial Sd | MIC | n | y | y | n | n | n | y | y | profound |
| 64 | 3158 | EIEE | MOCS2 | Molybdenum cofactor deficiency, classical | DBD | y | n.a. | y | n.a. | n | y | n.a. | n.a. | profound |
| 65 | 3163 | CBA-MIC | SIL1 | MSS | CBA | n | y | y | y | n | n | y | y | profound |
| 66 | 3172 | MIC | PARS2 | PARS2-related mtDNA depletion | MIC | y | y | y | n | y | n | y | n | profound |
| 67 | 3191 | MIC-HYH | ASPM | MCPH5 | MIC | n | y | y | y | y | n.a. | y | y | profound |
| 68 | 3200 | MIC-CCH | AP4M1 | SPG50 | MIC | n | y | y | n | y | n | y | y | severe |
| 69 | 3208 | MSGP | PHGDH | 3-PHGDH deficiency, infantile | MIC | n | y | y | n | n.a. | n | y | y | profound |
| 70 | 3226 | CBA | PLA2G6 | NBIA2B: neuroaxonal dystrophy, atypical | CBA | n | y | y | y | n | y | n | n | profound |
| 71 | 3310 | DBD | GALC | Krabbe globoid cell leukodystrophy, infantile | DBD | n | y | y | n | y | y | y | n.a. | profound |
| 72 | 3356 | DBD-NCL | PPT1 | CLN1 (INCL): infantile neuronal ceroid lipofuscinosis | DBD | y | y | y | n | n | y | y | n | profound |
| 73 | 3818 | WMD | PCDH12 | MIC, seizures, spasticity, and brain calcification | MIC | n | y | y | n | y | n.a. | y | y | severe |
| 74 | 4101 | Mito | SUCLA2 | MTDPS5: SUCLA2-related mtDNA depletion Sd | DBD | n | y | y | n | n | n | y | n.a. | profound |

**Supplementary Table 2. Clinical and imaging findings in 74 families that received prenatal diagnosis**

(y - finding present, n - finding not present, n.a. - data on the finding not available, Sd - syndrome abbreviations see Section S2)

| # | **Study #** | **FET #** | **Diagnosis** | **Gene** | **Mutation position (hg19)** | **# affected**  **before pregnancy** | **Results**  **of testing** | **Family decision** | **Outcome** |
| --- | --- | --- | --- | --- | --- | --- | --- | --- | --- |
| 1 | 520 | 1 | EBH | LAMB1 | c.3141_3158delinsGACCCAGTGCTTGTGTCTTCCTAATGTGCTTGTGTCTTCCTAAT | 3 | Hom ref | Delivery | Normal |
| 2 | 520 | 2 | EBH | LAMB1 | c.3141_3158delinsGACCCAGTGCTTGTGTCTTCCTAATGTGCTTGTGTCTTCCTAAT | 3 | Hom ref | Delivery | Normal |
| 3 | 702 | 1 | MMR | VPS13B | 8:100589861G>T | 4 | Het | Delivery | Normal |
| 4 | 711 | 1 | LIS-PMG | KATNB1 | 16:57789805T>G | 2 | Hom mut | Delivery | Affected |
| 5 | 718 | 1 | MIC | CIT | 12:120295424C>A | 4 | Hom mut | Delivery | Affected |
| 6 | 819 | 1 | PCH | TSEN54 | 17:73513639G>T | 2 | Het | Delivery | Normal |
| 7 | 819 | 2 | PCH | TSEN54 | 17:73513639G>T |  | Het | Delivery | Normal |
| 8 | 841 | 1 | MTI | AHI1 | 6:135752442C>T | 4 | Hom mut | Delivery | Affected |
| 9 | 841 | 2 | MTI | AHI1 | 6:135752442C>T | 4 | Hom mut | TOP | n.a. |
| 10 | 920 | 1 | LIS | RELN | 7:103207046A>C | 3 | Hom ref | Delivery | Normal |
| 11 | 923 | 1 | PCH | TSEN54 | 17:73518081G>T | 5 | Hom ref | Delivery | Normal |
| 12 | 997 | 1 | MTI | TMEM67 | 8:94767192T>A, 8:94817111G>C | 1 | Het | Delivery | Normal |
| 13 | 1004 | 1 | MIC | GJC2 | 1:228345562C>T | 2 | Hom mut | Delivery | Affected |
| 14 | 1010 | 1 | MTI | AHI1 | 6:135778720GTCTA>G | 1 | Hom mut | Delivery | Affected |
| 15 | 1190 | 1 | MTI | TMEM138 | 11:61135400AC>A | 2 | Hom mut | TOP | n.a. |
| 16 | 1261 | 1 | PMG | ADGRG1 | 16:57689383A>AG | 2 | Hom mut | TOP | n.a. |
| 17 | 1269 | 1 | MIC | ZNF335 | 20:44590754T>C | 2 | Hom ref | Delivery | Normal |
| 18 | 1269 | 2 | MIC | ZNF335 | 20:44590754T>C | 2 | Hom mut | TOP | n.a. |
| 19 | 1273 | 1 | MIC | POMT2 | 4:77746411A>G | 4 | Het | Delivery | Normal |
| 20 | 1273 | 2 | MIC | POMT2 | 4:77746411A>G | 4 | Hom mut | Delivery | Affected |
| 21 | 1368 | 1 | COACH | TMEM237 | 2:202498140AG>A | 1 | Hom mut | TOP | n.a. |
| 22 | 1373 | 1 | CBA | PIGN | 18:59780510C>T | 1 | Hom mut | Delivery | Affected |
| 23 | 1381 | 1 | WWS | POMGNT1 | 1:46658038ACCCAG>A | 1 | Het | Delivery | Normal |
| 24 | 1391 | 1 | PCH | AMPD2 | 1:110173362G>T | 1 | Het | Delivery | Normal |
| 25 | 1393 | 1 | HSP | ALS2 | 2:202591271C>T | 2 | Het | Delivery | Normal |
| 26 | 1406 | 1 | MRE | KCTD7 | 7:66104184C>T | 2 | Hom ref | Delivery | Normal |
| 27 | 1524 | 1 | MTI | C5ORF42 | 5:37231049C>CAA, 5:37213735T>C | 2 | Het | Delivery | Normal |
| 28 | 1548 | 1 | PCH | TSEN2 | 3:12573148T>C | 2 | Hom ref | Delivery | Normal |
| 29 | 1548 | 2 | PCH | TSEN2 | 3:12573148T>C | 2 | Het | Delivery | Normal |
| 30 | 1588 | 1 | CBA | FKTN | 9:108382302T>C | 1 | Hom mut | TOP | n.a. |
| 31 | 1592 | 1 | DMJD | PCDH12 | 5:141334905TC>T | 2 | Hom ref | Delivery | Normal |
| 32 | 1644 | 1 | MR-MIC | VPS13B | 8:100779134TTA>T | 1 | Het | Delivery | Normal |
| 33 | 1702 | 1 | ARA | ALDH5A1 | 6:24528264A>AC | 2 | Het | Delivery | Normal |
| 34 | 1713 | 1 | MR | CRBN | 3:3194228C>T | 3 | Hom ref | Delivery | Normal |
| 35 | 1743 | 1 | NCL | PPT1 | 1:40539819G>A | 2 | Hom ref | Delivery/STOP | n.a. |
| 36 | 1829 | 1 | HSP | NT5C2 | 10:104861028T>A | 2 | Het | Delivery | Normal |
| 37 | 1894 | 1 | MIC-CVH | ANK3 | 10:61829656CTG>C | 2 | Het | Delivery | Normal |
| 38 | 2006 | 1 | MIC-WMD | RNASEH2C | 11:65487533G>A | 2 | Het | Delivery | Normal |
| 39 | 2007 | 1 | MTI | ARMC9 | 2:232071007G>T | 2 | Hom mut | TOP | n.a. |
| 40 | 2013 | 1 | MRE | HACE1 | 6:105198348C>T | 3 | Hom ref | Delivery | Normal |
| 41 | 2020 | 1 | HYH | CENPJ | 13:25487031G>A | 2 | Het | Delivery | Normal |
| 42 | 2022 | 1 | MTI | AHI1 | 6:135768160G>A | 2 | Het | Delivery | Normal |
| 43 | 2027 | 1 | MIC-CBA | WDR73 | 15:85188983C>T | 2 | Hom ref | Delivery | Normal |
| 44 | 2028 | 1 | MSGP | ASPM | 1:197062286G>A | 2 | Het | Delivery | Normal |
| 45 | 2056 | 1 | CDG | SRD5A3 | 4:56225611G>A | 2 | Hom mut | Delivery | Affected |
| 46 | 2131 | 1 | PMG | WDR62 | 19:36577615C>T | 1 | Het | Delivery | Normal |
| 47 | 2132 | 1 | HSP | RNASEH2B | 13:51519581G>A | 2 | Hom ref | Delivery | Normal |
| 48 | 2236 | 1 | Martsolf | RAB3GAP1 | 2:135883820G>C | 1 | Het | Delivery | Normal |
| 49 | 2245 | 1 | PMG | ADGRG1 | 16:57685282C>T | 1 | Het | Delivery | Normal |
| 50 | 2247 | 2 | CS | ERCC8 | 5:60214191G>C | 2 | Het | Delivery | Normal |
| 51 | 2247 | 1 | CS | ERCC8 | 5:60214191G>C | 2 | Hom mut | TOP | n.a. |
| 52 | 2248 | 1 | CCH | MCOLN1 | 9:7590039TG>T | 2 | Het | Delivery | Normal |
| 53 | 2282 | 1 | MIC | ASPM | 1:197111484TTC>T | 2 | Het | Delivery | Normal |
| 54 | 2303 | 1 | Martsolf | RAB3GAP1 | 2:135893304G>A | 2 | Hom ref | Delivery | Normal |
| 55 | 2421 | 1 | MSS | TBC1D20 | 20:428590G>A | 2 | Het | Delivery | Normal |
| 56 | 2424 | 1 | MIC | ST3GAL5 | 2:86067500C>T | 2 | Hom mut | TOP | n.a. |
| 57 | 2424 | 2 | MIC | ST3GAL5 | 2:86067500C>T | 2 | Het | Delivery | Normal |
| 58 | 2450 | 1 | CMD | GMPPB | 3:49760037G>A | 2 | Het | Delivery | Normal |
| 59 | 2566 | 1 | MIC | PYCR2 | 1:226108909G>A | 1 | Hom mut | TOP | n.a. |
| 60 | 2641 | 1 | MTI | TMEM138 | 11:61135470G>A | 1 | Het | Delivery | Normal |
| 61 | 2643 | 1 | MIC | SLC1A4 | 2:65243654T>TG | 2 | Hom mut | TOP | n.a. |
| 62 | 2664 | 2 | MIC | PYCR2 | 1:226108909G>A | 1 | Hom ref | Delivery | Normal |
| 63 | 2664 | 1 | MIC | PYCR2 | 1:226108909G>A | 1 | Hom mut | TOP | n.a. |
| 64 | 2715 | 1 | PMG | POMGNT1 | 1:46657796C>T | 1 | Het | Delivery | Normal |
| 65 | 2715 | 2 | PMG | POMGNT1 | 1:46657796C>T | 1 | Hom ref | Delivery | Normal |
| 66 | 2730 | 1 | DBD | CLN2 | 11:6636216T>TAG, 11:6636204C>T | 2 | Het | Delivery | Normal |
| 67 | 2761 | 1 | AEM | PIGT | 20:44047543C>A, 20:44047955C>T | 2 | Hom ref | Delivery | Normal |
| 68 | 2887 | 1 | HSP | ALS2 | 2:202626188 C>A | 2 | Hom mut | TOP | n.a. |
| 69 | 2889 | 1 | MIC-CBA | WDR73 | 15:85188983C>T | 2 | Hom mut | TOP | n.a. |
| 70 | 2904 | 1 | NCL | KCTD7 | 7:66104041C>T | 1 | Het | Delivery | Normal |
| 71 | 3064 | 1 | MR | LINS1 | 15:101115275TCA>T | 2 | Hom ref | Delivery | Normal |
| 72 | 3127 | 1 | MIC | AP4M1 | 7:99703627G>GT | 2 | Het | Delivery | Normal |
| 73 | 3130 | 1 | MIC | UBE3B | 12:109959356G>C | 3 | Het | Delivery | Normal |
| 74 | 3158 | 1 | EIEE | MOCS2 | 5:52405557C>T | 3 | Het | Delivery | Normal |
| 75 | 3163 | 1 | CBA-MIC | SIL1 | 5:138282969C>G | 2 | Hom ref | Delivery | Normal |
| 76 | 3172 | 1 | MIC | PARS2 | 1:55223696C>A | 3 | Het | Delivery | Normal |
| 77 | 3191 | 1 | MIC-HYH | ASPM | 1:197112693 TC>T | 1 | Hom mut | TOP | n.a. |
| 78 | 3200 | 1 | MIC-CCH | AP4M1 | 7:99702686CG>C | 3 | Het | Delivery | Normal |
| 79 | 3208 | 1 | MSGP | PHGDH | 1:120285493G>A | 1 | Het | Delivery | Normal |
| 80 | 3226 | 1 | CBA | PLA2G6 | 22:38525460C>T | 2 | Hom mut | TOP | n.a. |
| 81 | 3310 | 1 | DBD | GALC | 14:88416259A>G | 2 | Hom ref | Delivery | Normal |
| 82 | 3356 | 1 | DBD-NCL | PPT1 | 1:40558158A>C | 2 | Het | Delivery | Normal |
| 83 | 3818 | 1 | WMD | PCDH12 | 5:141334650 CAG>C | 1 | Het | Delivery | Normal |
| 84 | 4101 | 1 | Mito | SUCLA2 | 13:48563018c.370T>C | 2 | Hom ref | Delivery | Normal |

**Supplementary Table 3. Detailed information on pathogenic variants, results of prenatal testing and pregnancy outcomes in 86 pregnancies that received amniocentesis.** Hom - homozygous, Het - heterozygous, mut – mutant, ref – reference, TOP - termination of pregnancy, STOP - spontaneous TOP, FET # - number of pregnancy with fetal testing, n.a. - data on outcome not available, for the clinical diagnosis abbreviations see Section S2. In column 6, patients with compound heterozygous mutations have two separate mutations listed. Families in which two subsequent pregnancies were assessed through PND are listed with ‘2’ in column 3.

| # | **Study#** | **Clinical Diagnosis** | **Gene** | **Genetic Diagnosis (SC/OMIM)** | **Disease group** | **EPI** | **Motor delay** | **ID** | **CBA or Ataxia** | **Spasticity** | **Regression** | **MIC or Atrophy** | **Dysmorphism** | **ID Score** |
| --- | --- | --- | --- | --- | --- | --- | --- | --- | --- | --- | --- | --- | --- | --- |
| 1 | 711 | LIS-PMG | KATNB1 | LIS6 | LIS/PMG | y | y | y | n | y | y | y | y | profound |
| 2 | 718 | MIC | CIT | MCPH17 | MIC | n | y | y | n | y | n | y | y | severe |
| 3 | 841 | MTI | AHI1 | JBTS3 | MTI | n | y | y | y | n.a. | n | n | y | profound |
| 4 | 1004 | MIC | GJC2 | HLD2-Pelizaeus-Merzbacher-Like | MIC | n | y | y | y | y | n | y | n | severe |
| 5 | 1010 | MTI | AHI1 | JBTS3 | MTI | n | y | y | y | n | n.a. | n | y | severe |
| 6 | 1273 | MIC | POMT2 | Muscular dystrophy-dystroglycanopathy B2 | MIC | n | y | y | n | y | n | y | y | severe |
| 7 | 1373 | CBA | PIGN | Multiple congen anom hypot seiz | CBA | y | y | y | n | n | n | y | y | profound |
| 8 | 2056 | CDG | SRD5A3 | CDG1Q | LIS/PMG | n | y | y | y | n.a. | n | y | n | severe |

**Supplementary Table 4. Detailed phenotype on offspring from families in which fetus genotyped as affected according to prenatal diagnostic testing.** (y - finding present, n - finding not present, n.a. - data on the finding not available, Sd - syndrome abbreviations see Section S2).

**References**

1. Novarino G, Fenstermaker AG, Zaki MS, Hofree M, Silhavy JL, Heiberg AD, Abdellateef M, Rosti B, Scott E, Mansour L, et al: Exome sequencing links corticospinal motor neuron disease to common neurodegenerative disorders. *Science* 2014, 343:506-511.

2. Dixon-Salazar TJ, Silhavy JL, Udpa N, Schroth J, Bielas S, Schaffer AE, Olvera J, Bafna V, Zaki MS, Abdel-Salam GH, et al: Exome sequencing can improve diagnosis and alter patient management. *Sci Transl Med* 2012, 4:138ra178.

3. Narasimhan V, Danecek P, Scally A, Xue Y, Tyler-Smith C, Durbin R: BCFtools/RoH: a hidden Markov model approach for detecting autozygosity from next-generation sequencing data. *Bioinformatics* 2016, 32:1749-1751.

4. Segal MM, Abdellateef M, El-Hattab AW, Hilbush BS, De La Vega FM, Tromp G, Williams MS, Betensky RA, Gleeson J: Clinical pertinence metric enables hypothesis-independent genome-phenome analysis for neurologic diagnosis. *J Child Neurol* 2015, 30:881-888.

5. Brownstein CA, Beggs AH, Homer N, Merriman B, Yu TW, Flannery KC, DeChene ET, Towne MC, Savage SK, Price EN, et al: An international effort towards developing standards for best practices in analysis, interpretation and reporting of clinical genome sequencing results in the CLARITY Challenge. *Genome Biol* 2014, 15:R53.
