# Supplementary material for: Molecular diagnosis in recessive pediatric neurogenetic disease can help reduce disease recurrence in families
Source: BMC Med Genomics. 2020 May 13;13:68. doi: 10.1186/s12920-020-0714-1 (PMC7218834; doi:10.1186/s12920-020-0714-1)
Supplement: Supplementary file 2 — Additional file 2: Data S1. SimulConsult® Phenome-Genome output pages on each family qualifying for referral for amniocentesis. [file 12920_2020_714_MOESM2_ESM.pdf]

SimulConsult

Family 520

Summary for a 14 year old boy with:

Pertinent positive findings

* = required to be in diseases considered; onsets can be at an age, by an age, or unknown

Req'd	Onset	Finding	Pertinence
*	≤1y	MRI: hypomyelination type of white matter abnormality	
*	≤Bir	Encephalocele or cerebral meningocele	
	≤1y	CT or MRI: subcortical bands of gray matter	
*	≤1y	CT, MRI or head USG: hydrocephalus, not ex-vacuo	
*	≤1y	CT or MRI: pan-cerebellar atrophy or hypoplasia	
	≤3y	Regression	
	≤1y	CT or MRI: cerebral cortex atrophy or hypoplasia	
	≤1y	CT or MRI: brain cysts or cavities	
	≤3y	Seizures with abnormal movements	
	≤1y	Motor developmental delay	
	≤3y	Intellectual disability	
	≤3y	Hypotonia	
*	≤1y	CT or MRI: lissencephaly	
	≤3y	Apraxia	
	≤3m	Nystagmus, non-rotary	

high→

Family history

Family history based on known clinical findings

1 of 1 sisters affected
1 of 1 brothers affected
Mother not affected
Father not affected
Consanguinity: 1st cousin

Pertinent gene findings from the variant table

Severity scores shown on left, with maximum of 5, and potential compound heterozygotes marked with "C"

Severity	Finding	Pertinence
5	LAMB1 gene variants (biallelic)	
5	PEX1 gene variants (biallelic)	
4	MSH6 gene variant (monoallelic)	
4	CYP2D6 gene variant (monoallelic)	
4	TTN gene variant (monoallelic)	

high→

Differential diagnosis

Disease	Probability
LIS5: LAMB1-related lissencephaly, AR	
Muscular dystrophy-dystroglycanopathy A4	
Galloway-Mowat syndrome 1, WDR73-related	
Adams-Oliver syndrome 2, DOCK6-related	
Muscular dystrophy-dystroglycanopathy A12	
Muscular dystrophy-dystroglycanopathy A2	
Muscular dystrophy-dystroglycanopathy A7	
CMV, symptomatic congenital infection	
Muscular dystrophy-dystroglycanopathy A3	
MCPH17: microcephaly, primary AR, CIT-related	

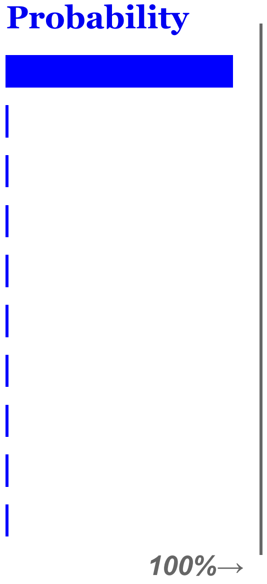

Most useful tests for this patient

Top tests ranked by usefulness in narrowing the differential, taking into account cost and treatability

Order	Test
☐	Creatine kinase high
☐	Eye: anterior chamber abnormalities
☐	Bundle: EMG (electromyogram)
☐	EMG: myopathic changes
☐	CT or MRI: vermal cerebellar atrophy or hypoplasia

Generated by SimulConsult® on 30 April 2018 13:55 using software of 20 March 2018 12:14 and database of 24 April 2018 7:07.

Disease incidence was used. Onset was ignored. Genome used.

SimulConsult

Family 702

Summary for a 19 year old man with:

Pertinent positive findings

* = required to be in diseases considered; onsets can be at an age, by an age, or unknown

Req'd	Onset	Finding	Pertinence
*	≤1y	Digits: slender fingers and toes	
	≤6y	Teeth: macrodontia	
*	≤3y	Microcephaly	
	≤3y	Motor developmental delay	
	≤3y	Intellectual disability	
			high→

Family history

Family history based on known clinical findings

0 of 2 maternal uncles affected
0 of 1 sisters affected
0 of 1 brothers affected
3 of 11 nearby contacts affected
Mother not affected
Father not affected
Consanguinity: 3rd cousin

Pertinent gene findings from the variant table

Severity scores shown on left, with maximum of 5, and potential compound heterozygotes marked with "C"

Severity	Finding	Pertinence
5	VPS13B (COH1) gene variants (biallelic)	
4	SCN1A gene variant (monoallelic)	
5	KCTD7 gene variants (biallelic)	
4	IFIH1 gene variant (monoallelic)	
2C	GLYCTK gene variants (biallelic)	
		high→

Differential diagnosis

Disease	Probability
Cohen syndrome	
Chromosome 22q11.2 deletion syndrome	
Chromosome 3q29 recurrent deletion syndrome	
Pitt-Hopkins syndrome	
Chromosome 18q deletion syndrome	
Mowat-Wilson syndrome	
Emanuel syndrome	
Mandibulofacial dysostosis with microcephaly	
Koolen-De Vries syndrome	
Kaufman oculocerebrofacial syndrome	
	100%→

Most useful tests for this patient

Top tests ranked by usefulness in narrowing the differential, taking into account cost and treatability

Order	Test
☐	Retinal dystrophy or atrophy
☐	ERG (electroretinogram) abnormal
☐	Pigmentary retinopathy
☐	Neutropenia
☐	Bundle: CBC: complete blood count

Generated by SimulConsult® on 30 April 2018 14:22 using software of 20 March 2018 12:14 and database of 24 April 2018 7:07.
Disease incidence was used. Onset was ignored. Genome used.

SimulConsult

Family 711

Summary for a 16 year old girl with:

Pertinent positive findings

* = required to be in diseases considered; onsets can be at an age, by an age, or unknown

Req'd	Onset	Finding	Pertinence
*	≤1y	CT or MRI: lissencephaly	
*	≤1y	Microcephaly	
*	≤3y	Seizures with abnormal movements	
	≤1y	Motor developmental delay	
	≤1y	Intellectual disability	

high→

Family history

Family history based on known clinical findings

0 of 1 brothers affected
1 of 7 nearby contacts affected
Mother not affected
Father not affected
Consanguinity: 1st cousin

Pertinent gene findings from the variant table

Severity scores shown on left, with maximum of 5, and potential compound heterozygotes marked with "C"

Severity	Finding	Pertinence
5	KATNB1 gene variants (biallelic)	
4	BRAF gene variant (monoallelic)	
3	EFTUD2 gene variant (monoallelic)	
5	RAI1 gene variant (monoallelic)	
3	TAF1 gene variant (X-linked)	

high→

Differential diagnosis

Disease

- LIS6: KATNB1-related lissencephaly, AR
- CMV, symptomatic congenital infection
- CFC1: cardiofaciocutaneous syndrome, BRAF-related
- Mandibulofacial dysostosis with microcephaly
- Chromosome 1p36 deletion syndrome
- LIS2: RELN-related lissencephaly, AR
- EIEE10: epileptic encephalopathy, early infantile, PNKP-related
- MCPH2: microcephaly, primary AR, WDR62-related
- PCH10: pontocerebellar hypoplasia, CLP1-related
- Muscular dystrophy-dystroglycanopathy A4

Probability

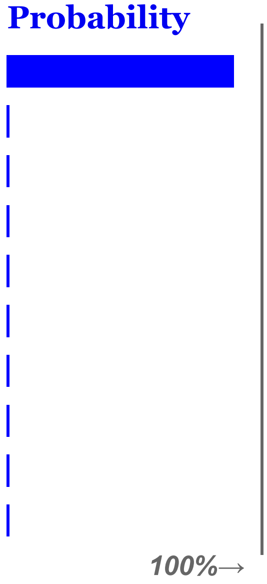

Most useful tests for this patient

Top tests ranked by usefulness in narrowing the differential, taking into account cost and treatability

Order	Test
☐	CT or MRI: subcortical bands of gray matter
☐	CT or MRI: heterotopias or subependymal nodules
☐	CT or MRI: thick cortex
☐	MRI: polymicrogyria
☐	MRI: white matter abnormality

Generated by SimulConsult® on 30 April 2018 14:37 using software of 20 March 2018 12:14 and database of 24 April 2018 7:07.
Disease incidence was used. Onset was ignored. Genome used.

SimulConsult

Family 718

Summary for a 11 year old girl with:

Pertinent positive findings

* = required to be in diseases considered; onsets can be at an age, by an age, or unknown

Req'd	Onset	Finding	Pertinence
	≤1y	Hypertelorism	
*	≤3y	CT or MRI: cerebral cortex atrophy or hypoplasia	
	≤1y	Hyperreflexia	
*	≤Bir	Microcephaly	
	≤1y	Epicanthus	
*	≤1y	Motor developmental delay	
*	≤1y	Intellectual disability	

high→

Pertinent negative findings

Absent	Finding	Pertinence
X	Seizures with abnormal movements	
X	Hearing impairment	

high→

Family history

Family history based on known clinical findings

1 of 1 sisters affected
2 of 2 brothers affected
Mother not affected
Father not affected
Consanguinity: 1st cousin

Pertinent gene findings from the variant table

Severity scores shown on left, with maximum of 5, and potential compound heterozygotes marked with "C"

Severity	Finding	Pertinence
5	CIT gene variants (biallelic)	
2	GRIA4 gene variant (monoallelic)	
4	TRIO gene variant (monoallelic)	
5C	NTRK1 gene variants (biallelic)	
2	CDON gene variant (monoallelic)	

high→

Differential diagnosis

Disease	Probability
MCPH17: microcephaly, primary AR, CIT-related	
Galloway-Mowat syndrome 1, WDR73-related	
Galloway-Mowat syndrome 5, TPRKB-related	
Aspartylglucosaminuria	
MRT61: mental retardation, AR, RUSC2-related	
CMV, symptomatic congenital infection	
PEHO syndrome	
Encephalopathy, progressive, early-onset with brain atrophy and thin corpus callosum	
Spastic tetraplegia, thin corpus callosum, and progressive microcephaly	
Aicardi-Goutières syndrome, AR	
	100%→

Most useful tests for this patient

Top tests ranked by usefulness in narrowing the differential, taking into account cost and treatability

Order	Test
☐	CT or MRI: lissencephaly
☐	Nephrotic degree to proteinuria
☐	Bundle: UA (urinalysis)
☐	Albumin low in serum
☐	Renal structural abnormalities

Generated by SimulConsult® on 30 April 2018 14:46 using software of 20 March 2018 12:14 and database of 24 April 2018 7:07.
Disease incidence was used. Onset was ignored. Genome used.

SimulConsult

Family 819

Summary for a 13 year old girl with:

Pertinent positive findings

* = required to be in diseases considered; onsets can be at an age, by an age, or unknown

Req'd	Onset	Finding	Pertinence
*	≤3y	Motor developmental delay	
*	≤3y	CT or MRI: pontine atrophy or hypoplasia	
	≤3y	Microcephaly	
*	≤3y	Intellectual disability	
	≤3y	Seizures with abnormal movements	
*	≤3y	CT or MRI: pan-cerebellar atrophy or hypoplasia	
	≤3y	Ataxia	

high→

Family history

Family history based on known clinical findings

1 of 2 brothers affected
Mother not affected
Father not affected
Consanguinity: 1st cousin

Pertinent gene findings from the variant table

Severity scores shown on left, with maximum of 5, and potential compound heterozygotes marked with "C"

Severity	Finding	Pertinence
5	TSEN54 gene variants (biallelic)	
3	ARID1A gene variant (monoallelic)	
3	LRP2 gene variants (biallelic)	
5	CAMK2B gene variant (monoallelic)	
5	MYH3 gene variant (monoallelic)	

high→

Differential diagnosis

Disease | **Probability**

- PCH2: pontocerebellar hypoplasia 2
- PCH4: pontocerebellar hypoplasia, TSEN54-related
- CDG1A: PMM2-related
- PCH11: pontocerebellar hypoplasia, TBC1D23-related
- PCH1B: pontocerebellar hypoplasia, EXOSC3-related
- VLDLR-related cerebellar hypoplasia
- PCH9: pontocerebellar hypoplasia, AMPD2
- PCH10: pontocerebellar hypoplasia, CLP1-related
- PCH1A: pontocerebellar hypoplasia, VRK1-related
- PCH3: pontocerebellar hypoplasia 3

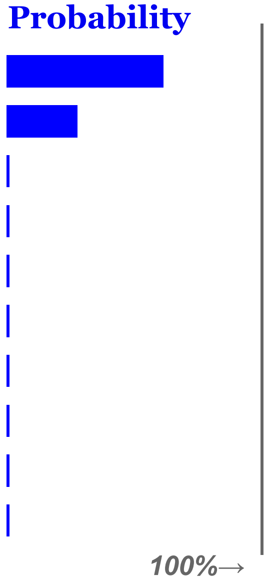

Most useful tests for this patient

Top tests ranked by usefulness in narrowing the differential, taking into account cost and treatability

Order	Test
☐	CT or MRI: cerebral gliosis
☐	Myoglobinuria
☐	CT or MRI: vermal cerebellar atrophy or hypoplasia
☐	CT or MRI: brain cysts or cavities
☐	CT or MRI: brainstem atrophy or hypoplasia

Generated by SimulConsult® on 30 April 2018 15:49 using software of 20 March 2018 12:14 and database of 24 April 2018 7:07.
Disease incidence was used. Onset was ignored. Genome used.

SimulConsult

Family 841

Summary for a 11 year old girl with:

Pertinent positive findings

* = required to be in diseases considered; onsets can be at an age, by an age, or unknown

Req'd	Onset	Finding	Pertinence
	≤3y	Mouth: tongue rhythmic movements, abnormal	
	≤3y	Nystagmus, non-rotary	
*	≤3y	Motor developmental delay	
*	≤3y	Intellectual disability	
	≤3y	Hypotonia	
	≤3y	CT or MRI: vermal cerebellar atrophy or hypoplasia	
*	≤3y	Oculomotor apraxia	
*	≤3y	CT or MRI: molar tooth sign on brain imaging	
	≤1y	Mouth: oral frenulum, accessory	
			high→

Family history

Family history based on known clinical findings

1 of 2 sisters affected
Mother not affected
Father not affected
Consanguinity: 1st cousin

Pertinent gene findings from the variant table

Severity scores shown on left, with maximum of 5, and potential compound heterozygotes marked with "C"

Severity	Finding	Pertinence
5	AHI1 gene variants (biallelic)	
2	VWF gene variant (monoallelic)	
2	HTRA1 gene variant (monoallelic)	
2	MEF2A gene variant (monoallelic)	
2	KCNA5 gene variant (monoallelic)	
		high→

Differential diagnosis

Disease	Probability
JBTS3: Joubert syndrome, AHI1-related	
JBTS30: Joubert syndrome, ARMC9-related	
JBTS1: Joubert syndrome, INPP5E-related	
JBTS25: Joubert syndrome, CEP104-related	
JBTS27: Joubert syndrome, B9D1-related	
JBTS28: Joubert syndrome, MKS1-related	
JBTS32: Joubert syndrome, SUFU-related	
JBTS26: Joubert syndrome, KIAA0556-related	
JBTS29: Joubert syndrome, TMEM107-related	
JBTS34: Joubert syndrome, B9D2-related	

100%→

Most useful tests for this patient

Top tests ranked by usefulness in narrowing the differential, taking into account cost and treatability

Order	Test
☐	Blood urea nitrogen (BUN) high
☐	ERG (electroretinogram) abnormal
☐	Renal structural abnormalities
☐	Pigmentary retinopathy
☐	CT or MRI: brainstem atrophy or hypoplasia

Generated by SimulConsult® on 30 April 2018 9:40 using software of 20 March 2018 12:14 and database of 24 April 2018 7:07.
Disease incidence was used. Onset was ignored. Genome used.

SimulConsult

Family 920

Summary for a 12 year old girl with:

Pertinent positive findings

* = required to be in diseases considered; onsets can be at an age, by an age, or unknown

Req'd	Onset	Finding	Pertinence
*	≤3y	CT or MRI: lissencephaly	
*	≤3y	Microcephaly	
	≤3y	Lactate high in serum	
	≤3y	Hypotonia	
*	≤3y	Intellectual disability	
*	≤3y	Motor developmental delay	
	≤3y	Ammonia high in plasma especially after protein	
			high→

Family history

Family history based on known clinical findings

1 of 1 sisters affected
1 of 1 brothers affected
Mother not affected
Father not affected
Consanguinity: 1st cousin

Pertinent gene findings from the variant table

Severity scores shown on left, with maximum of 5, and potential compound heterozygotes marked with "C"

Severity	Finding	Pertinence
5	RELN gene variants (biallelic)	
4	COL4A1 gene variant (monoallelic)	
4	ELAC2 gene variants (biallelic)	
3C	DCHS1 gene variants (biallelic)	
2	SDCCAG8 gene variants (biallelic)	
		high→

Differential diagnosis

Disease	Probability
LIS2: RELN-related lissencephaly, AR	
PEHO syndrome	
Encephalopathy, neonatal severe, with lactic acidosis and brain abnormalities	
Band-like calcification with simplified gyration and polymicrogyria	
Schizencephaly, familial	
COXPD17: combined OXPHOS deficiency, ELAC2-related	
MCPH17: microcephaly, primary AR, CIT-related	
CMV, symptomatic congenital infection	
Muscular dystrophy-dystroglycanopathy A4	
Galloway-Mowat syndrome 3, OSGEP-related	
	100%→

Most useful tests for this patient

Top tests ranked by usefulness in narrowing the differential, taking into account cost and treatability

Order	Test
☐	CT or MRI: pan-cerebellar atrophy or hypoplasia
☐	CT or MRI: thick cortex
☐	CT or MRI: cerebral cortex atrophy or hypoplasia
☐	MRI: white matter abnormality
☐	CT or MRI: corpus callosum hypogenesis

Generated by SimulConsult® on 27 April 2018 15:34 using software of 20 March 2018 12:14 and database of 24 April 2018 7:07.
Disease incidence was used. Onset was ignored. Genome used.

SimulConsult

Family 923

Summary for a 10 year old girl with:

Pertinent positive findings

* = required to be in diseases considered; onsets can be at an age, by an age, or unknown

Req'd	Onset	Finding	Pertinence
*	≤1y	Spasticity character to hypertonia	
*	≤3y	CT or MRI: pontine atrophy or hypoplasia	
	≤Bir	Microcephaly	
*	≤3y	Intellectual disability	
*	≤1y	Motor developmental delay	
*	≤3y	CT or MRI: pan-cerebellar atrophy or hypoplasia	
			high→

Family history

Family history based on known clinical findings

0 of 2 sisters affected
0 of 1 brothers affected
3 of 11 nearby contacts affected
Mother not affected
Father not affected
Consanguinity: 2nd cousin

Pertinent gene findings from the variant table

Severity scores shown on left, with maximum of 5, and potential compound heterozygotes marked with "C"

Severity	Finding	Pertinence
5	TSEN54 gene variants (biallelic)	
2	TBC1D24 gene variants (biallelic)	
4	MYH7 gene variant (monoallelic)	
4	IGSF1 gene variant (X-linked)	
4	CCDC88C gene variant (monoallelic)	
		high→

Differential diagnosis

Disease	Probability
PCH2: pontocerebellar hypoplasia 2	
PCH4: pontocerebellar hypoplasia, TSEN54-related	
PCH9: pontocerebellar hypoplasia, AMPD2	
PCH11: pontocerebellar hypoplasia, TBC1D23-related	
PCH10: pontocerebellar hypoplasia, CLP1-related	
PCH8: pontocerebellar hypoplasia, CHMP1A-related	
PCH3: pontocerebellar hypoplasia 3	
PEHO-like syndrome, CCDC88A-related	
Asparagine synthetase deficiency	
Pyruvate dehydrogenase E1α deficiency, late infantile	

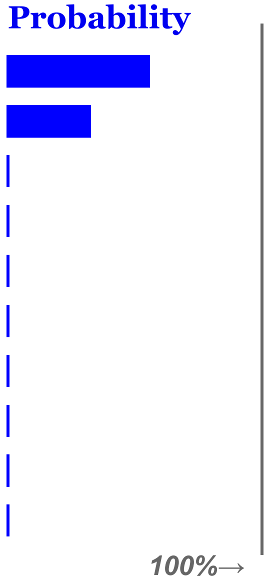

Most useful tests for this patient

Top tests ranked by usefulness in narrowing the differential, taking into account cost and treatability

Order	Test
☐	CT or MRI: cerebral gliosis
☐	CT or MRI: vermal cerebellar atrophy or hypoplasia
☐	Myoglobinuria
☐	CT or MRI: brain cysts or cavities
☐	CT or MRI: brainstem atrophy or hypoplasia

Generated by SimulConsult® on 30 April 2018 16:05 using software of 20 March 2018 12:14 and database of 24 April 2018 7:07.

Disease incidence was used. Onset was ignored. Genome used.

SimulConsult

Family 997

Summary for a 10 year old girl with:

Pertinent positive findings

* = required to be in diseases considered; onsets can be at an age, by an age, or unknown

Req'd	Onset	Finding	Pertinence
	≤1y	Eye movement deficit, horizontal	
	≤3y	Nose: nasal bridge, depressed	
*	≤3y	Intellectual disability	
*	≤3y	CT or MRI: corpus callosum hypogenesis	
*	≤3y	Nystagmus, non-rotary	
*	≤3y	Motor developmental delay	
*	≤3y	CT or MRI: molar tooth sign on brain imaging	
	≤3y	Ataxia	
*	≤1y	CT or MRI: vermal cerebellar atrophy or hypoplasia	
	≤1y	Mouth: wide	
	≤1y	EEG: spikes, focal	

high→

Family history

Family history based on known clinical findings

0 of 1 sisters affected
0 of 1 brothers affected
Mother not affected
Father not affected
Consanguinity: From same ethnic group

Pertinent gene findings from the variant table

Severity scores shown on left, with maximum of 5, and potential compound heterozygotes marked with "C"

Severity	Finding	Pertinence
4C	TMEM67 gene variants (biallelic)	
2	CHD7 gene variant (monoallelic)	
3	DYNC2H1 gene variant (monoallelic)	
2	ERBB4 gene variant (monoallelic)	
5	FREM1 gene variant (monoallelic)	

high→

Differential diagnosis

Disease	Probability
JBTS6: Joubert syndrome, TMEM67-related	
JBTS2: Joubert syndrome, TMEM216-related	
JBTS16: Joubert syndrome, TMEM138-related	
JBTS5: Joubert syndrome, CEP290-related	
JBTS3: Joubert syndrome, AHI1-related	
JBTS32: Joubert syndrome, SUFU-related	
JBTS24: Joubert syndrome, TCTN2-related	
JBTS15: Joubert syndrome, CEP41-related	
JBTS12: acrocallosal syndrome, KIF7-related	
JBTS18: Joubert syndrome, TCTN3-related	
	100%→

Most useful tests for this patient

Top tests ranked by usefulness in narrowing the differential, taking into account cost and treatability

Order	Test
☐	Transaminases (LFTs) high
☐	Retinal dystrophy or atrophy
☐	Bundle: Liver biopsy
☐	Liver biopsy: fibrosis or cirrhosis
☐	Renal structural abnormalities

Generated by SimulConsult® on 30 April 2018 16:21 using software of 20 March 2018 12:14 and database of 24 April 2018 7:07.

Disease incidence was used. Onset was ignored. Genome used.

SimulConsult

Family 1004

Summary for a 12 year old girl with:

Pertinent positive findings

* = required to be in diseases considered; onsets can be at an age, by an age, or unknown

Req'd	Onset	Finding	Pertinence
	≤3y	MRI: hypomyelination type of white matter abnormality	
*	≤3y	Hyperreflexia	
*	≤3y	Spasticity character to hypertonia	
	≤6y	Dysarthria or abnormal sound character	
	≤3y	Weakness, significant	
	≤1y	Nystagmus, non-rotary	
	≤3y	CT or MRI: pan-cerebellar atrophy or hypoplasia	
	≤3y	Intellectual disability	
*	≤3y	Motor developmental delay	
	≤3y	Gait disturbance	
	≤3y	Microcephaly	
*	≤3y	MRI: white matter abnormality	
			high→

Family history

Family history based on known clinical findings

0 of 1 sisters affected
1 of 1 brothers affected
Mother not affected
Father not affected
Consanguinity: 3rd cousin

Pertinent gene findings from the variant table

Severity scores shown on left, with maximum of 5, and potential compound heterozygotes marked with "C"

Severity	Finding	Pertinence
5	GJC2 gene variants (biallelic)	
3C	EIF2B5 gene variants (biallelic)	
2C	PEX1 gene variants (biallelic)	
2C	MMP21 gene variants (biallelic)	
2	DVL1 gene variant (monoallelic)	
		high→

Differential diagnosis

Disease	Probability
HLD2: Pelizaeus-Merzbacher-like disease 1	
Salla disease	
SPAX8: spastic ataxia, AR, with hypomyelinating leukodystrophy	
SPG75: spastic paraplegia, AR, MAG-related	
HLD6: hypomyelinating leukodystrophy, TUBB4A -related	
NBIA2A: INAD: infantile neuroaxonal dystrophy, classic	
SPG11: Spastic paraplegia, AR with thin corpus callosum, mental retardation	
Aicardi-Goutières syndrome, AR	
HLD1: Pelizaeus-Merzbacher disease, classic	
Neurodevelopmental disorder with progressive microcephaly, spasticity, and brain anomalies	

100%→

Most useful tests for this patient

Top tests ranked by usefulness in narrowing the differential, taking into account cost and treatability

Order	Test
☐	Bundle: Nerve conduction studies
☐	Nerve conduction: NCV slow, sensory
☐	Nerve conduction: NCV slow, motor
☐	Nerve conduction: low amplitude sensory action potentials
☐	CT or MRI: corpus callosum hypogenesis

Generated by SimulConsult® on 30 April 2018 16:33 using software of 20 March 2018 12:14 and database of 24 April 2018 7:07.

Disease incidence was used. Onset was ignored. Genome used.

SimulConsult

Family 1010

Summary for a 10 year old girl with:

Pertinent positive findings

* = required to be in diseases considered; onsets can be at an age, by an age, or unknown

Req'd	Onset	Finding	Pertinence
*	✓	CT or MRI: molar tooth sign on brain imaging	
	≤3y	Nystagmus, non-rotary	
	≤6y	Infections, frequent	
*	≤6y	Ataxia	
*	≤3y	Motor developmental delay	
	≤3y	Mouth: tongue, protruding	
	≤3y	Hypotonia	
	≤3y	Intellectual disability	

high→

Pertinent negative findings

Absent	Finding	Pertinence
X	Polyuria	
X	Renal failure or severe dysfunction	

high→

Family history

Family history based on known clinical findings

0 of 1 sisters affected
Mother not affected
Father not affected
Consanguinity: 1st cousin

Pertinent gene findings from the variant table

Severity scores shown on left, with maximum of 5, and potential compound heterozygotes marked with "C"

Severity	Finding	Pertinence
5	AHI1 gene variants (biallelic)	
4C	VPS13B (COH1) gene variants (biallelic)	
2	COL7A1 gene variant (monoallelic)	
2C	APOB gene variants (biallelic)	
2	RERE gene variant (monoallelic)	

high→

Differential diagnosis

Disease | **Probability**

- JBTS3: Joubert syndrome, AHI1-related
- JBTS12: acrocallosal syndrome, KIF7-related
- JBTS26: Joubert syndrome, KIAA0556-related
- Trisomy 21 (Down syndrome)
- JBTS24: Joubert syndrome, TCTN2-related
- JBTS13: Joubert syndrome, TCTN1-related
- JBTS9: Joubert syndrome, CC2D2A-related
- JBTS8: Joubert syndrome, ARL13B-related
- JBTS6: Joubert syndrome, TMEM67-related
- JBTS15: Joubert syndrome, CEP41-related

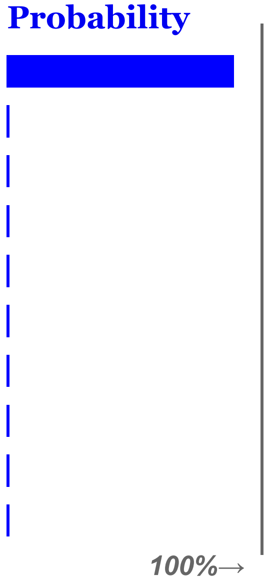

Most useful tests for this patient

Top tests ranked by usefulness in narrowing the differential, taking into account cost and treatability

Order	Test
☐	Pigmentary retinopathy
☐	Blood urea nitrogen (BUN) high
☐	Renal structural abnormalities
☐	MRI: polymicrogyria
☐	ERG (electroretinogram) abnormal

Generated by SimulConsult® on 30 April 2018 16:48 using software of 20 March 2018 12:14 and database of 24 April 2018 7:07.
Disease incidence was used. Onset was ignored. Genome used.

SimulConsult

Family 1190

Summary for a 9 year old boy with:

Pertinent positive findings

* = required to be in diseases considered; onsets can be at an age, by an age, or unknown

Req'd	Onset	Finding	Pertinence
	≤3y	Renal structural abnormalities	
*	≤1y	Nystagmus, non-rotary	
	≤Bir	Digits: polydactyly	
*	≤1y	CT or MRI: pan-cerebellar atrophy or hypoplasia	
*	≤3y	Motor developmental delay	
	≤3y	Respiratory difficulty	
*	≤3y	Intellectual disability	
	≤3y	Ataxia	
*	≤3y	Oculomotor apraxia	
	≤3y	CT or MRI: vermal cerebellar atrophy or hypoplasia	
*	≤3y	CT or MRI: molar tooth sign on brain imaging	
			high→

Family history

Family history based on known clinical findings

1 of 1 sisters affected
0 of 1 brothers affected
Mother not affected
Father not affected
Consanguinity: 1st cousin

Pertinent gene findings from the variant table

Severity scores shown on left, with maximum of 5, and potential compound heterozygotes marked with "C"

Severity	Finding	Pertinence
5	TMEM138 gene variants (biallelic)	
4	SLC3A1 gene variants (monoallelic)	
4	EVC gene variant (monoallelic)	
4	FBLN5 gene variant (monoallelic)	
4	CPT1A gene variants (biallelic)	
		high→

Differential diagnosis

Disease	Probability
JBTS16: Joubert syndrome, TMEM138-related	
OFD6: orofaciodigital syndrome 6	
JBTS12: acrocallosal syndrome, KIF7-related	
JBTS5: Joubert syndrome, CEP290-related	
JBTS3: Joubert syndrome, AHI1-related	
JBTS7: Joubert syndrome, RPGRIP1L-related	
JBTS21: Joubert syndrome, CSPP1-related	
JBTS20: Joubert syndrome, TMEM231-related	
JBTS9: Joubert syndrome, CC2D2A-related	
JBTS30: Joubert syndrome, ARMC9-related	

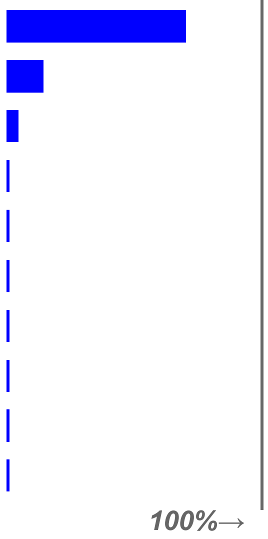

Most useful tests for this patient

Top tests ranked by usefulness in narrowing the differential, taking into account cost and treatability

Order	Test
☐	C5orf42 gene variants (biallelic)
☐	Pigmentary retinopathy
☐	Tongue biopsy: hamartomas
☐	Blood urea nitrogen (BUN) high
☐	Retinal dystrophy or atrophy

Generated by SimulConsult® on 30 April 2018 22:18 using software of 20 March 2018 12:14 and database of 24 April 2018 7:07.
Disease incidence was used. Onset was ignored. Genome used.

SimulConsult

Family 1261

Summary for a 10 year old boy with:

Pertinent positive findings

* = required to be in diseases considered; onsets can be at an age, by an age, or unknown

Req'd	Onset	Finding	Pertinence
*	≤1y	MRI: polymicrogyria	
	≤3y	Spasticity character to hypertonia	
	≤3y	Hypertonia / stiffness	
	≤1y	Myoclonus	
	≤1y	CT or MRI: cerebral cortex atrophy or hypoplasia	
*	≤1y	MRI: white matter abnormality	
*	≤3y	CT or MRI: pan-cerebellar atrophy or hypoplasia	
*	≤3y	Seizures with abnormal movements	
*	≤3y	Intellectual disability	
	≤3y	Hyperreflexia	
*	≤3y	Motor developmental delay	
	≤1y	Skin: hypopigmentation, generalized	

high→

Family history

Family history based on known clinical findings

1 of 2 sisters affected
Mother not affected
Father not affected
Consanguinity: 1st cousin

Pertinent gene findings from the variant table

Severity scores shown on left, with maximum of 5, and potential compound heterozygotes marked with "C"

Severity	Finding	Pertinence
5	ADGRG1 gene variants (biallelic)	
5	NPC1 gene variants (biallelic)	
2	PDHX gene variant (monoallelic)	
2C	NPHP4 gene variants (biallelic)	
2	AKT3 gene variant (monoallelic)	

high→

Differential diagnosis

Disease	Probability
Polymicrogyria, bilateral frontoparietal	
PEHO syndrome	
CMV, symptomatic congenital infection	
Galloway-Mowat syndrome 4, TP53RK-related	
D-bifunctional peroxisomal enzyme deficiency	
CLN8: neuronal ceroid lipofuscinosis, Turkish variant	
Aicardi-Goutières syndrome, AR	
Angelman syndrome	
PCH4: pontocerebellar hypoplasia, TSEN54-related	
Neurodevelopmental disorder with progressive microcephaly, spasticity, and brain anomalies	

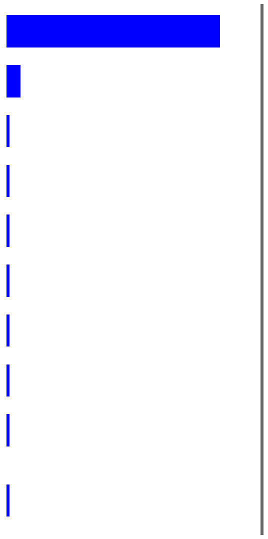

100%→

Most useful tests for this patient

Top tests ranked by usefulness in narrowing the differential, taking into account cost and treatability

Order	Test
☐	Bundle: EEG (electroencephalogram)
☐	EEG: spikes, generalized
☐	ZNHIT3 gene variants (biallelic)
☐	CT, MRI or head USG: hydrocephalus, not ex-vacuo
☐	EEG: hypsarrhythmia

Generated by SimulConsult® on 1 May 2018 9:35 using software of 20 March 2018 12:14 and database of 24 April 2018 7:07.

Disease incidence was used. Onset was ignored. Genome used.

SimulConsult

Family 1269

Summary for a 9 year old girl with:

Pertinent positive findings

* = required to be in diseases considered; onsets can be at an age, by an age, or unknown

Req'd	Onset	Finding	Pertinence
	≤1y	Seizures with abnormal movements	
*	≤1y	CT or MRI: corpus callosum hypogenesis	
	≤1y	MRI: white matter abnormality	
	≤3y	CT or MRI: cerebral cortex atrophy or hypoplasia	
*	≤1y	Hypertonia / stiffness	
	≤3y	Spasticity character to hypertonia	
*	≤1y	Microcephaly	
*	≤3y	Intellectual disability	
*	≤1y	Motor developmental delay	
			high→

Family history

Family history based on known clinical findings

1 of 1 brothers affected
Mother not affected
Father not affected
Consanguinity: 1st cousin

Pertinent gene findings from the variant table

Severity scores shown on left, with maximum of 5, and potential compound heterozygotes marked with "C"

Severity	Finding	Pertinence
5	ZNF335 gene variants (biallelic)	
2	ANKRD11 gene variant (monoallelic)	
2	GP1BA gene variant (monoallelic)	
2	SYNE2 gene variant (monoallelic)	
2	EPHB4 gene variant (monoallelic)	
		high→

Differential diagnosis

Disease

- MCPH10: microcephaly, primary AR, ZNF335-related
- Spastic tetraplegia, thin corpus callosum, and progressive microcephaly
- PCH9: pontocerebellar hypoplasia, AMPD2
- Neurodevelopmental disorder with progressive microcephaly, spasticity, and brain anomalies
- Neurodevelopmental disorder with microcephaly, hypotonia, & brain anomalies
- Galloway-Mowat syndrome 3, OSGEP-related
- PCH10: pontocerebellar hypoplasia, CLP1-related
- Encephalopathy, progressive, early-onset with brain atrophy and thin corpus callosum
- Webb-Dattani syndrome
- Epilepsy, hearing loss, and mental retardation syndrome

Probability

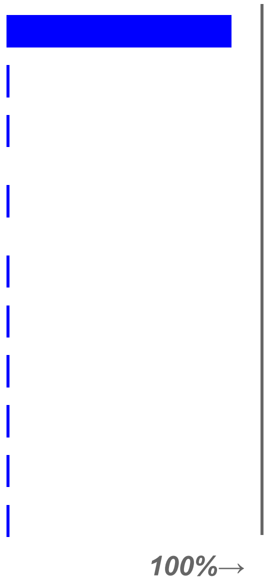

Most useful tests for this patient

Top tests ranked by usefulness in narrowing the differential, taking into account cost and treatability

Order	Test
☐	CT or MRI: cerebral gliosis
☐	CT or MRI: lissencephaly
☐	CT or MRI: pan-cerebellar atrophy or hypoplasia
☐	CT or MRI: brainstem atrophy or hypoplasia
☐	MRI: hypomyelination type of white matter abnormality

Generated by SimulConsult® on 1 May 2018 9:49 using software of 20 March 2018 12:14 and database of 24 April 2018 7:07.

Disease incidence was used. Onset was ignored. Genome used.

SimulConsult

Family 1273

Summary for a 14 year old boy with:

Pertinent positive findings

* = required to be in diseases considered; onsets can be at an age, by an age, or unknown

Req'd	Onset	Finding	Pertinence
*	≤3y	Microcephaly	
	≤6y	Scoliosis with or without kyphosis	
*	≤6y	EMG: myopathic changes	
*	≤3y	Intellectual disability	
*	≤3y	Motor developmental delay	
*	≤3y	Creatine kinase high	
*	≤3y	Hypotonia	
*	≤3y	Weakness, significant	
			high→

Family history

Family history based on known clinical findings

0 of 2 sisters affected
3 of 7 nearby contacts affected
Mother not affected
Father not affected
Consanguinity: 1st cousin

Pertinent gene findings from the variant table

Severity scores shown on left, with maximum of 5, and potential compound heterozygotes marked with "C"

Severity	Finding	Pertinence
5	POMT2 gene variants (biallelic)	
2	TRIP4 gene variants (biallelic)	
3	KIF5C gene variant (monoallelic)	
3	COL6A2 gene variant (monoallelic)	
3	GIGYF2 gene variant (monoallelic)	
		high→

Differential diagnosis

Disease | **Probability**

- Muscular dystrophy-dystroglycanopathy B2
- Muscular dystrophy-dystroglycanopathy A2
- Muscular dystrophy-dystroglycanopathy A4
- Muscular dystrophy-dystroglycanopathy B5
- Muscular dystrophy, congenital, with cataracts and intellectual disability
- Carey-Fineman-Ziter syndrome
- Marinesco-Sjögren syndrome
- Muscular dystrophy-dystroglycanopathy A1
- Muscular dystrophy-dystroglycanopathy A7
- Muscular dystrophy-dystroglycanopathy A3

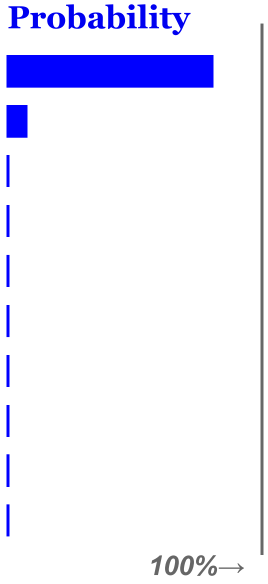

Most useful tests for this patient

Top tests ranked by usefulness in narrowing the differential, taking into account cost and treatability

Order	Test
☐	Bundle: MRI scan of the brain
☐	Bundle: CT scan of the brain
☐	Eye: anterior chamber abnormalities
☐	CT or MRI: vermal cerebellar atrophy or hypoplasia
☐	CT or MRI: lissencephaly

Generated by SimulConsult® on 1 May 2018 10:09 using software of 20 March 2018 12:14 and database of 24 April 2018 7:07.
Disease incidence was used. Onset was ignored. Genome used.

SimulConsult

Family 1368

Summary for a 8 year old boy with:

Pertinent positive findings

* = required to be in diseases considered; onsets can be at an age, by an age, or unknown

Req'd	Onset	Finding	Pertinence
	≤1y	Hypogonadism or cryptorchidism	
*	≤1y	Seizures with abnormal movements	
*	≤3y	Intellectual disability	
*	≤3y	Oculomotor apraxia	
	≤Bir	Chorioretinal coloboma	
	≤3m	Respiratory difficulty	
*	≤1y	Hypotonia	
*	≤3y	Motor developmental delay	
	≤1y	Hyperreflexia	
*	≤1y	CT or MRI: molar tooth sign on brain imaging	
	≤1y	Hepatomegaly	
	≤1y	Splenomegaly	
	≤3m	Tachycardia	

high→

Family history

Family history based on known clinical findings

Mother not affected
Father not affected
Consanguinity: 1st cousin

Pertinent gene findings from the variant table

Severity scores shown on left, with maximum of 5, and potential compound heterozygotes marked with "C"

Severity	Finding	Pertinence
4	TMEM237 gene variants (biallelic)	
2	TGFB2 gene variant (monoallelic)	
2C	SPATA16 gene variants (biallelic)	
2	STK11 gene variant (monoallelic)	
2	RAD54L gene variant (monoallelic)	

high→

Differential diagnosis

Disease	Probability
JBTS14: Joubert syndrome, TMEM237-related	
JBTS34: Joubert syndrome, B9D2-related	
JBTS15: Joubert syndrome, CEP41-related	
JBTS30: Joubert syndrome, ARMC9-related	
JBTS17: Joubert syndrome, C5orf42-related	
JBTS26: Joubert syndrome, KIAA0556-related	
JBTS12: acrocallosal syndrome, KIF7-related	
JBTS2: Joubert syndrome, TMEM216-related	
JBTS16: Joubert syndrome, TMEM138-related	
JBTS6: Joubert syndrome, TMEM67-related	

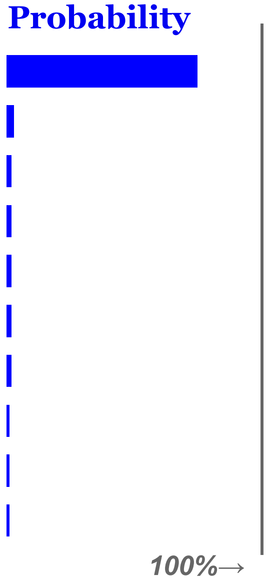

Most useful tests for this patient

Top tests ranked by usefulness in narrowing the differential, taking into account cost and treatability

Order	Test
☐	Blood urea nitrogen (BUN) high
☐	Renal structural abnormalities
☐	ERG (electroretinogram) abnormal
☐	CT, MRI or head USG: hydrocephalus, not ex-vacuo
☐	CT or MRI: brainstem atrophy or hypoplasia

Generated by SimulConsult® on 1 May 2018 10:21 using software of 20 March 2018 12:14 and database of 24 April 2018 7:07.

Disease incidence was used. Onset was ignored. Genome used.

SimulConsult

Family 1373

Summary for a 10 year old girl with:

Pertinent positive findings

* = required to be in diseases considered; onsets can be at an age, by an age, or unknown

Req'd	Onset	Finding	Pertinence
*	≤3y	CT or MRI: pan-cerebellar atrophy or hypoplasia	
*	≤3y	CT or MRI: cerebral cortex atrophy or hypoplasia	
	≤1y	Nose: nasal bridge, depressed	
	≤1y	Ears: auricles, dysplastic	
*	≤3y	Seizures with abnormal movements	
*	≤3y	Motor developmental delay	
	≤3y	Hypotonia	
*	≤3y	Intellectual disability	
	≤1y	Plantar creases, deep	
	≤1y	Palmar creases, deep	
			high→

Family history

Family history based on known clinical findings

Mother not affected
Father not affected
Consanguinity: 2nd cousin

Pertinent gene findings from the variant table

Severity scores shown on left, with maximum of 5, and potential compound heterozygotes marked with "C"

Severity	Finding	Pertinence
5	PIGN gene variants (biallelic)	
4	SMARCA4 gene variant (monoallelic)	
3	ARID1B gene variant (monoallelic)	
3	SHANK3 gene variant (monoallelic)	
5	SETBP1 gene variant (monoallelic)	
		high→

Differential diagnosis

Disease	Probability
Multiple congenital anomalies-hypotonia-seizures 1	
Pallister-Killian syndrome	
PCH3: pontocerebellar hypoplasia 3	
Zhu-Tokita-Takenouchi-Kim syndrome	
Multiple congenital anomalies-hypotonia-seizures 3	
Neurodevelopmental disorder with microcephaly, hypotonia, & brain anomalies	
Costello syndrome	
MTDPS13: FBXL4 encephalomyopathic mtDNA depletion syndrome	
Fetal alcohol syndrome	
Wolf-Hirschhorn syndrome	

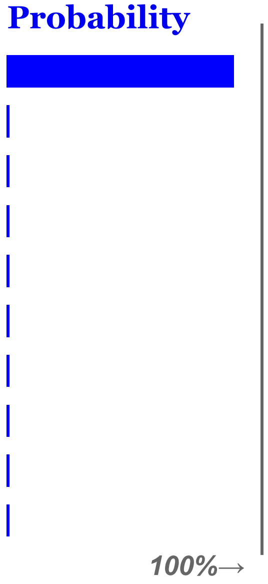

Most useful tests for this patient

Top tests ranked by usefulness in narrowing the differential, taking into account cost and treatability

Order	Test
☐	Bundle: Echocardiogram
☐	Imaging: patent foramen ovale
☐	Imaging: patent ductus arteriosus
☐	CT or MRI: corpus callosum hypogenesis
☐	Imaging: atrial septal defect

Generated by SimulConsult® on 27 April 2018 16:28 using software of 20 March 2018 12:14 and database of 24 April 2018 7:07.
Disease incidence was used. Onset was ignored. Genome used.

SimulConsult

Family 1381

Summary for a 11 year old boy with:

Pertinent positive findings

* = required to be in diseases considered; onsets can be at an age, by an age, or unknown

Req'd	Onset	Finding	Pertinence
*	≤3y	CT, MRI or head USG: hydrocephalus, not ex-vacuo	
	≤1y	Myopia, severe	
*	≤3y	CT or MRI: brain cysts or cavities	
*	≤3y	Intellectual disability	
*	≤3y	Motor developmental delay	
	≤1y	Hypotonia	
	≤3y	MRI: white matter abnormality	
*	≤1y	Creatine kinase high	
*	≤3y	EMG: myopathic changes	
*	≤1y	CT or MRI: lissencephaly	
*	≤1y	CT or MRI: vermal cerebellar atrophy or hypoplasia	
*	≤3y	Muscle biopsy: myopathic or dystrophic changes	
			high→

Family history

Family history based on known clinical findings

Mother not affected
Father not affected
Consanguinity: 1st cousin

Pertinent gene findings from the variant table

Severity scores shown on left, with maximum of 5, and potential compound heterozygotes marked with "C"

Severity	Finding	Pertinence
5	POMGNT1 gene variants (biallelic)	
2	TNXB gene variant (monoallelic)	
2	MYO1A gene variant (monoallelic)	
2	GDF5 gene variant (monoallelic)	
2	DTNA gene variant (monoallelic)	
		high→

Differential diagnosis

Disease	Probability
Muscular dystrophy-dystroglycanopathy A3	
Muscular dystrophy-dystroglycanopathy A1	
Muscular dystrophy-dystroglycanopathy A4	
Muscular dystrophy-dystroglycanopathy A11	
Muscular dystrophy-dystroglycanopathy A2	
Muscular dystrophy-dystroglycanopathy A5	
Muscular dystrophy-dystroglycanopathy A6	
Muscular dystrophy-dystroglycanopathy A12	
Muscular dystrophy-dystroglycanopathy A7	
Muscular dystrophy-dystroglycanopathy A10	
	100%→

Most useful tests for this patient

Top tests ranked by usefulness in narrowing the differential, taking into account cost and treatability

Order	Test
☐	Retinal dystrophy or atrophy
☐	ERG (electroretinogram) abnormal
☐	Bundle: EEG (electroencephalogram)
☐	EEG: spikes, generalized
☐	CT or MRI: brainstem atrophy or hypoplasia

Generated by SimulConsult® on 1 May 2018 10:56 using software of 20 March 2018 12:14 and database of 24 April 2018 7:07.
Disease incidence was used. Onset was ignored. Genome used.

SimulConsult

Family 1391
Summary for a 4 year old boy with:

Pertinent positive findings

* = required to be in diseases considered; onsets can be at an age, by an age, or unknown

Req'd	Onset	Finding	Pertinence
*	≤1y	CT or MRI: pontine atrophy or hypoplasia	
*	≤1y	MRI: white matter abnormality	
	≤1y	CT or MRI: corpus callosum hypogenesis	
*	≤1y	Spasticity character to hypertonia	
	≤1y	Hypertonia / stiffness	
	≤1y	Hyperreflexia	
*	≤1y	CT or MRI: pan-cerebellar atrophy or hypoplasia	
*	≤3y	Seizures with abnormal movements	
*	≤1y	CT or MRI: cerebral cortex atrophy or hypoplasia	
*	≤1y	Motor developmental delay	
*	≤1y	Intellectual disability	

high→

Family history

Family history based on known clinical findings

0 of 2 sisters affected
0 of 1 brothers affected
Mother not affected
Father not affected
Consanguinity: 1st cousin

Pertinent gene findings from the variant table

Severity scores shown on left, with maximum of 5, and potential compound heterozygotes marked with "C"

Severity	Finding	Pertinence
5	AMPD2 gene variants (biallelic)	
2	DNASE1 gene variant (monoallelic)	
2C	PLCB1 gene variants (biallelic)	
2	LPL gene variant (monoallelic)	
2	LRP4 gene variant (monoallelic)	

high→

Differential diagnosis

Disease	Probability
PCH9: pontocerebellar hypoplasia, AMPD2	
PCH10: pontocerebellar hypoplasia, CLP1-related	
Asparagine synthetase deficiency	
Pyruvate dehydrogenase E1α deficiency, early infantile	
PCH7: pontocerebellar hypoplasia 7	
Pyruvate dehydrogenase E1α deficiency, late infantile	
PCH4: pontocerebellar hypoplasia, TSEN54-related	
PCH2: pontocerebellar hypoplasia 2	
Isolated sulfite oxidase deficiency, classic early-onset	
PCH3: pontocerebellar hypoplasia 3	

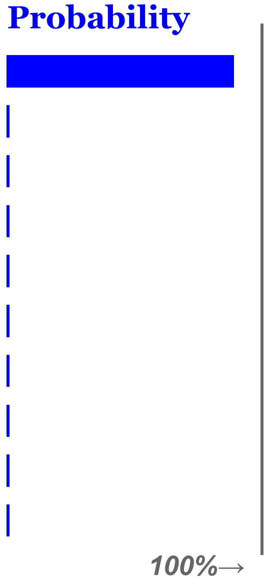

Most useful tests for this patient

Top tests ranked by usefulness in narrowing the differential, taking into account cost and treatability

Order	Test
☐	CT or MRI: brainstem atrophy or hypoplasia
☐	MRI: hypomyelination type of white matter abnormality
☐	Lactate high in serum
☐	Lactate high in CSF
☐	Metabolic acidosis

Generated by SimulConsult® on 10 May 2018 16:13 using software of 9 May 2018 09:46 and database of 8 May 2018 10:16.
Disease incidence was used. Onset was ignored. Genome used.

SimulConsult

Family 1393

Summary for a 10 year old girl with:

Pertinent positive findings

* = required to be in diseases considered; onsets can be at an age, by an age, or unknown

Req'd	Onset	Finding	Pertinence
*	≤3y	Motor developmental delay	
*	≤3y	Spasticity character to hypertonia	
*	≤3y	Hypertonia / stiffness	
*	≤3y	Weakness, significant	
			high→

Family history

Family history based on known clinical findings

1 of 1 nearby contacts affected
Mother not affected
Father not affected
Consanguinity: 2nd cousin

Pertinent gene findings from the variant table

Severity scores shown on left, with maximum of 5, and potential compound heterozygotes marked with "C"

Severity	Finding	Pertinence
5	ALS2 gene variants (biallelic)	
3	BICD2 gene variant (monoallelic)	
5	PYGM gene variant (monoallelic)	
5	COL6A3 gene variants (biallelic)	
5	COL6A3 gene variant (monoallelic)	
		high→

Differential diagnosis

Disease	Probability
Infantile ascending hereditary spastic paraplegia	
Juvenile primary lateral sclerosis	
Toxoplasmosis, symptomatic congenital infection	
CP: cerebral palsy, mild	
CP: cerebral palsy, severe	
Folate deficiency, cerebral	
Phenylketonuria	
Spastic paraplegia, optic atrophy, and neuropathy	
MRXS13: mental retardation, X-linked syndromic, MECP2-related	
MTDPS4A: POLG-related mtDNA depletion	

100%→

Most useful tests for this patient

Top tests ranked by usefulness in narrowing the differential, taking into account cost and treatability

Order	Test
☐	Bundle: CT scan of the brain
☐	Bundle: MRI scan of the brain
☐	CT or MRI: cerebral cortex atrophy or hypoplasia
☐	SER abnormal
☐	X-ray or CT: brain calcifications

Generated by SimulConsult® on 1 May 2018 11:23 using software of 20 March 2018 12:14 and database of 24 April 2018 7:07.
Disease incidence was used. Onset was ignored. Genome used.

SimulConsult

Family 1406

Summary for a 16 year old girl with:

Pertinent positive findings

* = required to be in diseases considered; onsets can be at an age, by an age, or unknown

Req'd	Onset	Finding	Pertinence
*	≤3y	Regression	
*	≤3y	Microcephaly	
*	≤3y	Intellectual disability	
*	≤3y	Motor developmental delay	
*	@3y	Seizures with abnormal movements	
	≤3y	ABR abnormal	
			high→

Family history

Family history based on known clinical findings

1 of 1 brothers affected
Mother not affected
Father not affected
Consanguinity: 1st cousin

Pertinent gene findings from the variant table

Severity scores shown on left, with maximum of 5, and potential compound heterozygotes marked with "C"

Severity	Finding	Pertinence
5	KCTD7 gene variants (biallelic)	
4	SCN1A gene variant (monoallelic)	
4	KAT6B gene variant (monoallelic)	
2	ANKRD11 gene variant (monoallelic)	
3	KIF4A gene variant (X-linked)	
		high→

Differential diagnosis

Disease | **Probability**

- EPM3: progressive myoclonic epilepsy 3
- EIEE6: Dravet syndrome (SCN1A-related)
- HLD6: hypomyelinating leukodystrophy, TUBB4A -related
- Gaucher disease type 2, acute infantile neuronopathic
- Creatine deficiency: GAMT creatine synthesis
- Krabbe globoid cell leukodystrophy, infantile
- MTDPS4A: POLG-related mtDNA depletion
- Aicardi-Goutières syndrome, AR
- Folate deficiency, cerebral
- Arginase deficiency

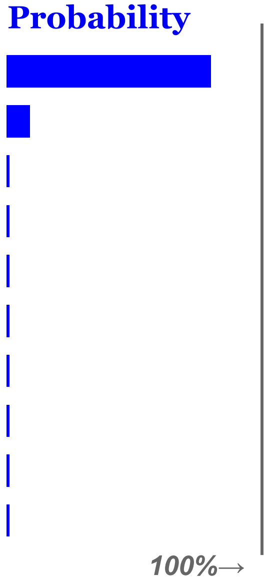

Most useful tests for this patient

Top tests ranked by usefulness in narrowing the differential, taking into account cost and treatability

Order	Test
☐	Bundle: EEG (electroencephalogram)
☐	EEG: spikes, focal
☐	Bundle: MRI scan of the brain
☐	Bundle: CT scan of the brain
☐	CT or MRI: cerebral cortex atrophy or hypoplasia

Generated by SimulConsult® on 1 May 2018 11:30 using software of 20 March 2018 12:14 and database of 24 April 2018 7:07.

Disease incidence was used. Onset was ignored. Genome used.

SimulConsult®

Family 1524

Summary for a 7 year old girl with:

Pertinent positive findings

* = required to be in diseases considered; onsets can be at an age, by an age, or unknown

Req'd	Onset	Finding	Pertinence
	≤3y	CT or MRI: cerebral cortex atrophy or hypoplasia	
	≤3y	Intellectual disability	
*	≤3y	Motor developmental delay	
	≤3y	Respiratory difficulty	
*	≤3y	Oculomotor apraxia	
	≤3y	Hypotonia	
	≤3y	CT or MRI: vermal cerebellar atrophy or hypoplasia	
*	≤3y	CT or MRI: molar tooth sign on brain imaging	
	≤3y	Mouth: tongue rhythmic movements, abnormal	

high→

Pertinent negative findings

Absent	Finding	Pertinence
X	Digits: polydactyly	

high→

Family history

Family history based on known clinical findings

0 of 1 sisters affected
0 of 1 brothers affected
Mother not affected
Father not affected
Consanguinity: From same village

Pertinent gene findings from the variant table

Severity scores shown on left, with maximum of 5, and potential compound heterozygotes marked with "C"

Severity	Finding	Pertinence
5C	C5orf42 gene variants (biallelic)	
2	DYNC1H1 gene variant (monoallelic)	
2	KIF2A gene variant (monoallelic)	
2	IQSEC2 gene variant (X-linked)	
2	ABCA1 gene variant (monoallelic)	

high→

Differential diagnosis

Disease	Probability
OFD6: orofaciodigital syndrome 6	
JBTS17: Joubert syndrome, C5orf42-related	
JBTS30: Joubert syndrome, ARMC9-related	
JBTS1: Joubert syndrome, INPP5E-related	
JBTS12: acrocallosal syndrome, KIF7-related	
JBTS26: Joubert syndrome, KIAA0556-related	
JBTS25: Joubert syndrome, CEP104-related	
JBTS29: Joubert syndrome, TMEM107-related	
JBTS28: Joubert syndrome, MKS1-related	
JBTS27: Joubert syndrome, B9D1-related	
	100%→

Most useful tests for this patient

Top tests ranked by usefulness in narrowing the differential, taking into account cost and treatability

Order	Test
☐	Tongue biopsy: hamartomas
☐	Pigmentary retinopathy
☐	CT or MRI: 4th ventricle enlargement, major
☐	Retinal dystrophy or atrophy
☐	X-ray: metacarpal bone deformed, hypoplastic or absent

Generated by SimulConsult® on 1 May 2018 12:46 using software of 20 March 2018 12:14 and database of 24 April 2018 7:07.
Disease incidence was used. Onset was ignored. Genome used.

SimulConsult

Family 1548

Summary for a 8 year old boy with:

Pertinent positive findings

* = required to be in diseases considered; onsets can be at an age, by an age, or unknown

Req'd	Onset	Finding	Pertinence
*	≤3y	CT or MRI: pontine atrophy or hypoplasia	
	≤3y	CT or MRI: brain cysts or cavities	
*	≤3y	CT or MRI: pan-cerebellar atrophy or hypoplasia	
	≤1y	Ears large	
	≤3y	CT or MRI: corpus callosum hypogenesis	
	≤3y	Spasticity character to hypertonia	
*	≤3y	Intellectual disability	
*	≤3y	Motor developmental delay	
*	≤3y	CT or MRI: cerebral cortex atrophy or hypoplasia	
	≤3y	Microcephaly	

high→

Family history

Family history based on known clinical findings

0 of 1 sisters affected
1 of 1 brothers affected
Mother not affected
Father not affected
Consanguinity: 1st cousin

Pertinent gene findings from the variant table

Severity scores shown on left, with maximum of 5, and potential compound heterozygotes marked with "C"

Severity	Finding	Pertinence
5	TSEN2 gene variants (biallelic)	
4	HPS3 gene variant (monoallelic)	
4	CACNA1B gene variant (monoallelic)	
4	LAMB3 gene variant (monoallelic)	
4	PTH1R gene variant (monoallelic)	

high→

Differential diagnosis

Disease	Probability
PCH2: pontocerebellar hypoplasia 2	
Asparagine synthetase deficiency	
Pyruvate dehydrogenase E1α deficiency, early infantile	
Pyruvate dehydrogenase E1α deficiency, late infantile	
PCH4: pontocerebellar hypoplasia, TSEN54-related	
PCH3: pontocerebellar hypoplasia 3	
Galloway-Mowat syndrome 1, WDR73-related	
PCH1B: pontocerebellar hypoplasia, EXOSC3-related	
Muscular dystrophy-dystroglycanopathy B2	
PCH7: pontocerebellar hypoplasia 7	

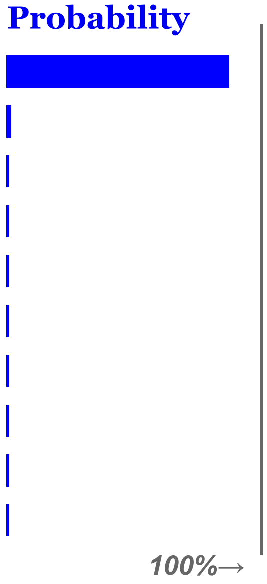

Most useful tests for this patient

Top tests ranked by usefulness in narrowing the differential, taking into account cost and treatability

Order	Test
☐	TSEN54 gene variants (biallelic)
☐	Creatine kinase high
☐	CT or MRI: brainstem atrophy or hypoplasia
☐	ASNS gene variants (biallelic)
☐	Bundle: EEG (electroencephalogram)

Generated by SimulConsult® on 27 April 2018 16:41 using software of 20 March 2018 12:14 and database of 24 April 2018 7:07.
Disease incidence was used. Onset was ignored. Genome used.

SimulConsult

Family 1588

Summary for a 11 year old girl with:

Pertinent positive findings

* = required to be in diseases considered; onsets can be at an age, by an age, or unknown

Req'd	Onset	Finding	Pertinence
*	≤3y	Muscular atrophy or hypoplasia	
	≤1y	CT or MRI: vermal cerebellar atrophy or hypoplasia	
	≤3y	CT or MRI: brain cysts or cavities	
	≤3y	Intellectual disability	
	≤3y	CT or MRI: pan-cerebellar atrophy or hypoplasia	
*	≤3y	Muscular hypertrophy	
	≤3y	Hypotonia	
*	≤3y	Creatine kinase high	
	≤3y	Weakness, significant	
*	≤3y	Motor developmental delay	
*	≤3y	Muscle biopsy: α-dystroglycan hypoglycosylation	
	≤3y	Ataxia	

high→

Family history

Family history based on known clinical findings

Mother not affected
Father not affected
Consanguinity: 2nd cousin

Pertinent gene findings from the variant table

Severity scores shown on left, with maximum of 5, and potential compound heterozygotes marked with "C"

Severity	Finding	Pertinence
5	FKTN gene variants (biallelic)	
4	ATP2B3 gene variant (X-linked)	
4	SYP gene variant (X-linked)	
4	LPL gene variant (monoallelic)	
4	ANK2 gene variant (monoallelic)	

high→

Differential diagnosis

Disease | **Probability**

- Muscular dystrophy-dystroglycanopathy A4
- Muscular dystrophy-dystroglycanopathy B5
- Muscular dystrophy-dystroglycanopathy B1
- Muscular dystrophy-dystroglycanopathy A2
- Muscular dystrophy-dystroglycanopathy C4
- Muscular dystrophy-dystroglycanopathy B2
- Muscular dystrophy-dystroglycanopathy B6
- Muscular dystrophy-dystroglycanopathy C5
- Muscular dystrophy-dystroglycanopathy C3
- CDG1A: PMM2-related

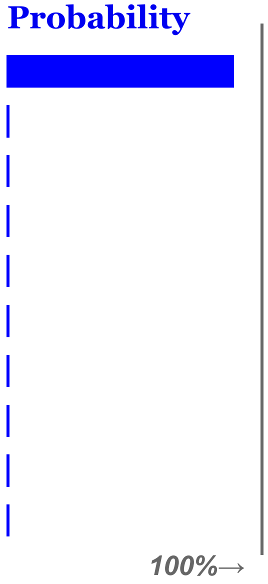

Most useful tests for this patient

Top tests ranked by usefulness in narrowing the differential, taking into account cost and treatability

Order	Test
☐	Eye: anterior chamber abnormalities
☐	CT or MRI: cerebral cortex atrophy or hypoplasia
☐	Bundle: Echocardiogram
☐	MRI: polymicrogyria
☐	Imaging: dilated cardiomyopathy

Generated by SimulConsult® on 1 May 2018 13:09 using software of 20 March 2018 12:14 and database of 24 April 2018 7:07.

Disease incidence was used. Onset was ignored. Genome used.

SimulConsult

Family 1592

Summary for a 7 year old boy with:

Pertinent positive findings

* = required to be in diseases considered; onsets can be at an age, by an age, or unknown

Req'd	Onset	Finding	Pertinence
	≤1y	Ears large	
	≤1y	Hypertelorism	
	≤1y	Forehead, sloping	
	≤1y	Ears low-set	
	≤1y	Hyperreflexia	
*	≤1m	Seizures with abnormal movements	
*	@Bir	Microcephaly	
*	≤1y	Hypertonia / stiffness	
*	≤3y	Intellectual disability	
*	≤1y	Motor developmental delay	
	≤1y	Basal ganglia nature to brain calcifications	

high→

Family history

Family history based on known clinical findings

0 of 1 sisters affected
1 of 1 brothers affected
Mother not affected
Father not affected
Consanguinity: 1st cousin

Pertinent gene findings from the variant table

Severity scores shown on left, with maximum of 5, and potential compound heterozygotes marked with "C"

Severity	Finding	Pertinence
5	PCDH12 gene variants (biallelic)	
2C	PLA2G6 gene variants (biallelic)	
5	NOTCH1 gene variant (monoallelic)	
2	NTRK2 gene variant (monoallelic)	
4	FGF8 gene variant (monoallelic)	

high→

Differential diagnosis

Disease	Probability
Microcephaly, seizures, spasticity, and brain calcification	
Galloway-Mowat syndrome 3, OSGEP-related	
Microcephaly-capillary malformation syndrome	
Cockayne syndrome I, moderate or classic	
Microhydranencephaly	
MTDPS4A: POLG-related mtDNA depletion	
Cockayne syndrome II, severe or early-onset	
MECP2 duplication syndrome, classical	
MCPH17: microcephaly, primary AR, CIT-related	
Asparagine synthetase deficiency	
	100%→

Most useful tests for this patient

Top tests ranked by usefulness in narrowing the differential, taking into account cost and treatability

Order	Test
☐	X-ray or CT: brain calcifications
☐	OSGEP gene variants (biallelic)
☐	Nephrotic degree to proteinuria
☐	Bundle: UA (urinalysis)
☐	Albumin low in serum

Generated by SimulConsult® on 1 May 2018 13:26 using software of 20 March 2018 12:14 and database of 24 April 2018 7:07.
Disease incidence was used. Onset was ignored. Genome used.

SimulConsult

Family 1644

Summary for a 8 year old boy with:

Pertinent positive findings

* = required to be in diseases considered; onsets can be at an age, by an age, or unknown

Req'd	Onset	Finding	Pertinence
	≤1y	Ears large	
	≤1y	Palpebral fissure downslanted	
*	≤1y	Microcephaly	
	≤1y	Facial weakness	
	≤1y	Ears low-set	
	≤3y	CT or MRI: corpus callosum hypogenesis	
	≤3y	Hyporeflexia	
	≤1y	Weakness, significant	
*	≤3y	Intellectual disability	
*	≤3y	Hypotonia	
	≤3y	Motor developmental delay	
			high→

Family history

Family history based on known clinical findings

0 of 1 brothers affected
Mother not affected
Father not affected
Consanguinity: 1st cousin

Pertinent gene findings from the variant table

Severity scores shown on left, with maximum of 5, and potential compound heterozygotes marked with "C"

Severity	Finding	Pertinence
5	VPS13B (COH1) gene variants (biallelic)	
2C	ABCD3 gene variants (biallelic)	
5	MBD5 gene variant (monoallelic)	
2C	HSPG2 gene variants (biallelic)	
4	DMD gene variant (X-linked)	
		high→

Differential diagnosis

Disease	Probability
Cohen syndrome	
Muscular dystrophy-dystroglycanopathy A4	
Muscular dystrophy-dystroglycanopathy B2	
Muscular dystrophy-dystroglycanopathy B1	
Corpus callosum agenesis and polyneuropathy	
Coffin-Lowry syndrome, males	
CHARGE syndrome	
IHPRF2: infantile hypotonia, psychomotor retardation, characteristic facies	
Complex cortical dysplasia with other brain malformations 1	
Vici syndrome	

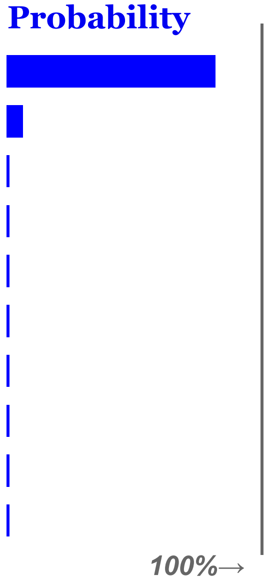

Most useful tests for this patient

Top tests ranked by usefulness in narrowing the differential, taking into account cost and treatability

Order	Test
☐	Retinal dystrophy or atrophy
☐	Creatine kinase high
☐	ERG (electroretinogram) abnormal
☐	Eye: anterior chamber abnormalities
☐	FKTN gene variants (biallelic)

Generated by SimulConsult® on 1 May 2018 13:47 using software of 20 March 2018 12:14 and database of 24 April 2018 7:07.
Disease incidence was used. Onset was ignored. Genome used.

SimulConsult

Family 1702

Summary for a 13 year old girl with:

Pertinent positive findings

* = required to be in diseases considered; onsets can be at an age, by an age, or unknown

Req'd	Onset	Finding	Pertinence
*	≤3y	Motor developmental delay	
*	≤3y	Ataxia	
	≤3y	Hypertonia / stiffness	
*	≤3y	CT or MRI: pan-cerebellar atrophy or hypoplasia	
*	≤3y	Intellectual disability	
			high→

Pertinent negative findings

Absent	Finding	Pertinence
X	Nerve conduction: NCV slow, motor	
X	EMG: neurogenic (denervation) changes	
		high→

Family history

Family history based on known clinical findings

1 of 2 sisters affected
Mother not affected
Father not affected
Consanguinity: 1st cousin

Pertinent gene findings from the variant table

Severity scores shown on left, with maximum of 5, and potential compound heterozygotes marked with "C"

Severity	Finding	Pertinence
5	ALDH5A1 gene variants (biallelic)	
4	SPG7 gene variants (biallelic)	
2	GCLC gene variants (biallelic)	
2	SMARCA4 gene variant (monoallelic)	
5	TTBK2 gene variant (monoallelic)	
		high→

Differential diagnosis

Disease	Probability
Succinic semialdehyde dehydrogenase deficiency	
SCAR20: Spinocerebellar ataxia, AR, SNX14-related	
CLN8: neuronal ceroid lipofuscinosis, Turkish variant	
PCH11: pontocerebellar hypoplasia, TBC1D23-related	
CLN7: neuronal ceroid lipofuscinosis, Gypsy variant	
SCAR2: Spinocerebellar ataxia, AR, congenital nonprogressive	
HLD11: leukodystrophy, hypomyelinating, POLR1C-related	
NBIA2B: neuroaxonal dystrophy, atypical	
PCH8: pontocerebellar hypoplasia, CHMP1A-related	
Polymicrogyria, bilateral frontoparietal	

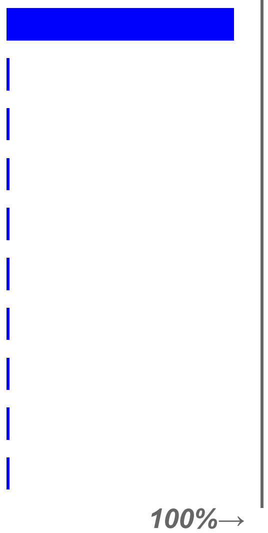

100%→

Most useful tests for this patient

Top tests ranked by usefulness in narrowing the differential, taking into account cost and treatability

Order	Test
☐	Bundle: Organic acids in urine
☐	SSADH activity low in leukocytes
☐	4-hydroxybutyric acid high in urine
☐	4-hydroxybutyric acid high in CSF
☐	4-hydroxybutyric acid high in blood

Generated by SimulConsult® on 1 May 2018 13:57 using software of 20 March 2018 12:14 and database of 24 April 2018 7:07.

Disease incidence was used. Onset was ignored. Genome used.

SimulConsult

Family 1713

Summary for a 12 year old boy with:

Pertinent positive findings

* = required to be in diseases considered; onsets can be at an age, by an age, or unknown

Req'd	Onset	Finding	Pertinence
*	≤3y	Intellectual disability	
*	≤3y	Motor developmental delay	

high→

Pertinent negative findings

Absent	Finding	Pertinence
X	Dysarthria or abnormal sound character	
X	Ears: auricles, dysplastic	
X	Seizures with abnormal movements	
X	Autistic behavior	

high→

Family history

Family history based on known clinical findings

1 of 2 sisters affected
Mother not affected
Father not affected

Pertinent gene findings from the variant table

Severity scores shown on left, with maximum of 5, and potential compound heterozygotes marked with "C"

Severity	Finding	Pertinence
5	CRBN gene variants (biallelic)	
5	KRAS gene variant (monoallelic)	
4	FOXG1 gene variant (monoallelic)	
5	ERCC8 gene variant (monoallelic)	
4	DYNC1H1 gene variant (monoallelic)	

high→

Differential diagnosis

Disease / **Probability**

- MRT2: mental retardation, AR, CRBN-related
- NS3: Noonan syndrome, KRAS-related
- CFC2: cardiofaciocutaneous syndrome, KRAS-related
- Rett syndrome, congenital variant
- CMV, symptomatic congenital infection
- Cockayne syndrome II, severe or early-onset
- Cockayne syndrome I, moderate or classic
- MRD13: mental retardation, AD, DYNC1H1-related
- Toxoplasmosis, symptomatic congenital infection
- CMT2O: DYNC1H1-related

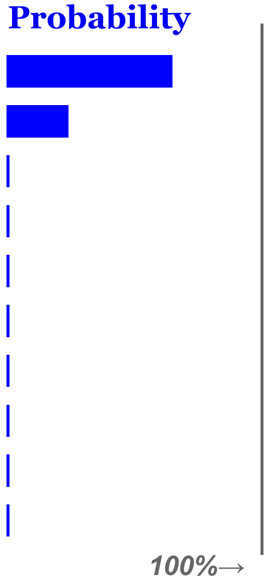

Most useful tests for this patient

Top tests ranked by usefulness in narrowing the differential, taking into account cost and treatability

Order	Test
☐	ECG: rhythm or conduction abnormalities
☐	Bundle: Echocardiogram
☐	X-ray: bone age delayed
☐	Bundle: Arthritis
☐	X-ray: vertebrae abnormal

Generated by SimulConsult® on 1 May 2018 14:28 using software of 20 March 2018 12:14 and database of 24 April 2018 7:07.
Disease incidence was used. Onset was ignored. Genome used.

SimulConsult

Family 1743

Summary for a 11 year old boy with:

Pertinent positive findings

* = required to be in diseases considered; onsets can be at an age, by an age, or unknown

Req'd	Onset	Finding	Pertinence
*	≤6y	Motor developmental delay	
*	≤3y	Regression	
*	≤3y	Visual impairment despite lens correction	
*	≤6y	Spasticity character to hypertonia	
*	≤3y	Seizures with abnormal movements	
*	≤6y	Intellectual disability	
			high→

Family history

Family history based on known clinical findings

1 of 1 brothers affected
Mother not affected
Father not affected
Consanguinity: 1st cousin

Pertinent gene findings from the variant table

Severity scores shown on left, with maximum of 5, and potential compound heterozygotes marked with "C"

Severity	Finding	Pertinence
5	PPT1 gene variants (biallelic)	
4	SLC1A2 gene variant (monoallelic)	
5	FAM83H gene variant (monoallelic)	
5	MYO7A gene variant (monoallelic)	
5	CACNA1B gene variant (monoallelic)	
		high→

Differential diagnosis

Disease

CLN1 (INCL): infantile neuronal ceroid lipofuscinosis

CLN3: juvenile neuronal ceroid lipofuscinosis

EIEE41: early infantile epileptic encephalopathy, SLC1A2-related

CLN7: neuronal ceroid lipofuscinosis, Gypsy variant

MTDPS4A: POLG-related mtDNA depletion

Aicardi-Goutières syndrome, AR

CLN8: neuronal ceroid lipofuscinosis, Turkish variant

Refsum disease, infantile

PARS2-related mtDNA depletion

NAGA deficiency I: Schindler disease type I

Probability

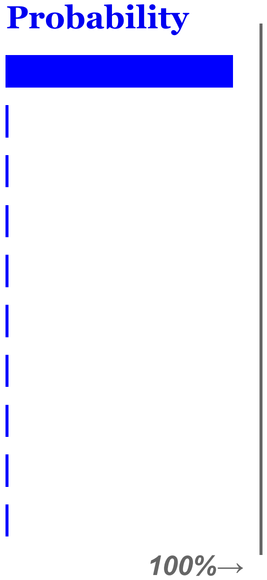

100%→

Most useful tests for this patient

Top tests ranked by usefulness in narrowing the differential, taking into account cost and treatability

Order	Test
☐	Bundle: Skin biopsy
☐	Palmitoyl-protein thioesterase activity low in leukocytes or fibroblasts
☐	EM: lymphocytes with granular osmophilic deposits inclusions on electron microscopy
☐	ERG (electroretinogram) abnormal
☐	Skin biopsy: granular osmophilic deposits inclusions in EM

Generated by SimulConsult® on 1 May 2018 14:41 using software of 20 March 2018 12:14 and database of 24 April 2018 7:07.
Disease incidence was used. Onset was ignored. Genome used.

SimulConsult

Family 1829

Summary for a 15 year old boy with:

Pertinent positive findings

* = required to be in diseases considered; onsets can be at an age, by an age, or unknown

Req'd	Onset	Finding	Pertinence
*	≤3y	Gait disturbance	
	≤3y	CT or MRI: corpus callosum hypogenesis	
	≤3y	Intellectual disability	
*	≤3y	Contractures or passive limited range of motion	
	≤3y	Spasticity character to hypertonia	
*	≤3y	Motor developmental delay	
*	≤3y	Hypertonia / stiffness	
			high→

Family history

Family history based on known clinical findings

0 of 3 sisters affected
1 of 2 brothers affected
2 of 5 nearby contacts affected
Mother not affected
Father not affected
Consanguinity: 1st cousin

Pertinent gene findings from the variant table

Severity scores shown on left, with maximum of 5, and potential compound heterozygotes marked with "C"

Severity	Finding	Pertinence
5	NT5C2 gene variants (biallelic)	
4	NIPBL gene variant (monoallelic)	
2	COQ8A gene variants (biallelic)	
4	CHST6 gene variants (biallelic)	
4C	VPS13C gene variants (biallelic)	
		high→

Differential diagnosis

Disease	Probability
SPG45: spastic paraplegia, AR, NT5C2-related	
FG syndrome	
SPG11: Spastic paraplegia, AR with thin corpus callosum, mental retardation	
SPG54: spastic paraplegia 54	
PCH8: pontocerebellar hypoplasia, CHMP1A-related	
MECP2 duplication syndrome, classical	
HLD1: Pelizaeus-Merzbacher disease, connatal	
HLD1: Pelizaeus-Merzbacher disease, classic	
MECP2 duplication syndrome with cytogenetically visibility	
PLP1 null syndrome	

100%→

Most useful tests for this patient

Top tests ranked by usefulness in narrowing the differential, taking into account cost and treatability

Order	Test
☐	MRI: white matter abnormality
☐	CT or MRI: pan-cerebellar atrophy or hypoplasia
☐	Creatine kinase high
☐	CT or MRI: cerebral cortex atrophy or hypoplasia
☐	ABR abnormal

Generated by SimulConsult® on 30 April 2018 10:15 using software of 20 March 2018 12:14 and database of 24 April 2018 7:07.
Disease incidence was used. Onset was ignored. Genome used.

SimulConsult

Family 1894

Summary for a 8 year old girl with:

Pertinent positive findings

* = required to be in diseases considered; onsets can be at an age, by an age, or unknown

Req'd	Onset	Finding	Pertinence
	≤3y	Hyperreflexia	
*	≤1y	Hypotonia	
	≤3y	Microcephaly	
	≤3y	CT or MRI: vermal cerebellar atrophy or hypoplasia	
*	≤3y	Intellectual disability	
*	≤3y	Motor developmental delay	
			high→

Family history

Family history based on known clinical findings

0 of 1 sisters affected
Mother not affected
Father not affected
Consanguinity: 1st cousin

Pertinent gene findings from the variant table

Severity scores shown on left, with maximum of 5, and potential compound heterozygotes marked with "C"

Severity	Finding	Pertinence
5	ANK3 gene variants (biallelic)	
5	CIC gene variant (monoallelic)	
3	KAT6B gene variant (monoallelic)	
2C	VPS13B (COH1) gene variants (biallelic)	
5	FLNB gene variant (monoallelic)	
		high→

Differential diagnosis

Disease	Probability
MRT37: mental retardation, AR, ANK3-related	
PCH11: pontocerebellar hypoplasia, TBC1D23-related	
Microcephaly, seizures, cerebral and cerebellar atrophy	
MRD45: mental retardation, AD, CIC-related	
Genitopatellar syndrome	
Mevalonic aciduria, infantile	
EIEE49: early infantile epileptic encephalopathy, DENND5A-related	
Vici syndrome	
HLD6: hypomyelinating leukodystrophy, TUBB4A -related	
Cohen syndrome	

100%→

Most useful tests for this patient

Top tests ranked by usefulness in narrowing the differential, taking into account cost and treatability

Order	Test
☐	CT or MRI: corpus callosum hypogenesis
☐	MRI: white matter abnormality
☐	CT or MRI: pan-cerebellar atrophy or hypoplasia
☐	CT or MRI: cerebral cortex atrophy or hypoplasia
☐	MRI: hypomyelination type of white matter abnormality

Generated by SimulConsult® on 1 May 2018 14:54 using software of 20 March 2018 12:14 and database of 24 April 2018 7:07.

Disease incidence was used. Onset was ignored. Genome used.

SimulConsult®

Family 2006

Summary for a 6 year old girl with:

Pertinent positive findings

* = required to be in diseases considered; onsets can be at an age, by an age, or unknown

Req'd	Onset	Finding	Pertinence
*	≤3y	Basal ganglia nature to brain calcifications	
*	≤3y	Seizures with abnormal movements	
	≤3y	CT or MRI: basal ganglia abnormalities	
*	≤3y	Motor developmental delay	
	≤1y	Hyperreflexia	
	≤3y	Hypertonia / stiffness	
*	≤3y	Intellectual disability	
*	≤3y	CT or MRI: pan-cerebellar atrophy or hypoplasia	
	≤3y	CT or MRI: brainstem atrophy or hypoplasia	
*	≤3y	CT or MRI: cerebral cortex atrophy or hypoplasia	
	≤3y	MRI: white matter abnormality	
	≤1y	Facial weakness	
	≤1y	Ears low-set	
	≤1y	Ears large	

high→

Family history

Family history based on known clinical findings

1 of 3 sisters affected
Mother not affected
Father not affected
Consanguinity: 1st cousin

Pertinent gene findings from the variant table

Severity scores shown on left, with maximum of 5, and potential compound heterozygotes marked with "C"

Severity	Finding	Pertinence
5	RNASEH2C gene variants (biallelic)	
4	COL5A1 gene variant (monoallelic)	
4	TMEM43 gene variant (monoallelic)	
4	PKD2 gene variant (monoallelic)	
4	FAT4 gene variants (biallelic)	

high→

Differential diagnosis

Disease — **Probability**

- Aicardi-Goutières syndrome, AR
- Cerebrotendinous xanthomatosis
- CMV, symptomatic congenital infection
- Biotinidase deficiency, profound
- Muscular dystrophy-dystroglycanopathy A4
- HLD14: leukodystrophy, hypomyelinating, UFM1-related
- Hyperornithinemia-hyperammonemia-homocitrullinuria syndrome
- PCH9: pontocerebellar hypoplasia, AMPD2
- Wilson disease
- HLD10: leukodystrophy, hypomyelinating, PYCR2-related

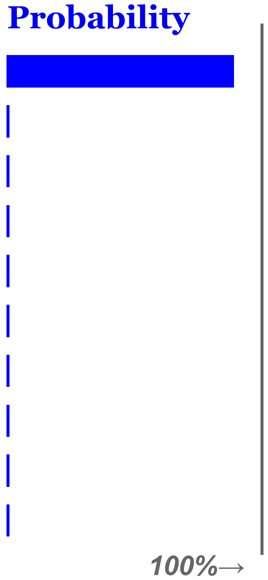

100%→

Most useful tests for this patient

Top tests ranked by usefulness in narrowing the differential, taking into account cost and treatability

Order	Test
☐	WBC high in CSF
☐	Interferon-α in the CSF elevated
☐	X-ray or CT: brain calcifications
☐	Bundle: MR spectroscopy
☐	Neopterin high in CSF

Generated by SimulConsult® on 1 May 2018 15:24 using software of 20 March 2018 12:14 and database of 24 April 2018 7:07.

Disease incidence was used. Onset was ignored. Genome used.

SimulConsult

Family 2007

Summary for a 16 year old girl with:

Pertinent positive findings

* = required to be in diseases considered; onsets can be at an age, by an age, or unknown

Req'd	Onset	Finding	Pertinence
	@Bir	Breath holding	
	≤3y	Eyes: ptosis	
*	≤1y	Intellectual disability	
	≤1y	Hypotonia	
*	≤3y	Nystagmus, non-rotary	
*	≤3y	Ataxia	
*	≤3m	Motor developmental delay	
*	≤3y	Oculomotor apraxia	
*	≤3y	CT or MRI: molar tooth sign on brain imaging	

high→

Pertinent negative findings

Absent	Finding	Pertinence
X	Digits: polydactyly	
X	Visual impairment despite lens correction	
X	Encephalocele or cerebral meningocele	
X	Regression	

high→

Family history

Family history based on known clinical findings

0 of 2 sisters affected
1 of 1 brothers affected
Mother not affected
Father not affected
Consanguinity: 1st cousin

Pertinent gene findings from the variant table

Severity scores shown on left, with maximum of 5, and potential compound heterozygotes marked with "C"

Severity	Finding	Pertinence
3	ARMC9 gene variants (biallelic)	
4	SLC5A2 gene variant (monoallelic)	
4C	DCHS1 gene variants (biallelic)	
5	ATP6V0A2 gene variant (monoallelic)	
5	AGXT gene variant (monoallelic)	

high→

Differential diagnosis

Disease | **Probability**

- JBTS30: Joubert syndrome, ARMC9-related
- JBTS1: Joubert syndrome, INPP5E-related
- JBTS26: Joubert syndrome, KIAA0556-related
- JBTS33: Joubert syndrome, PIBF1-related
- JBTS29: Joubert syndrome, TMEM107-related
- JBTS28: Joubert syndrome, MKS1-related
- JBTS27: Joubert syndrome, B9D1-related
- JBTS25: Joubert syndrome, CEP104-related
- JBTS9: Joubert syndrome, CC2D2A-related
- JBTS13: Joubert syndrome, TCTN1-related

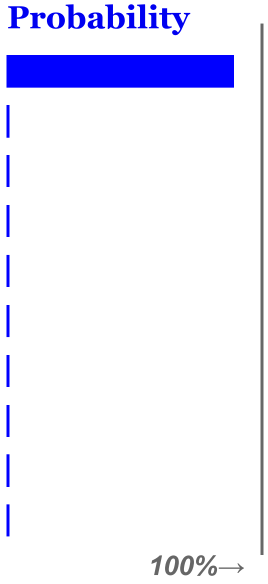

Most useful tests for this patient

Top tests ranked by usefulness in narrowing the differential, taking into account cost and treatability

Order	Test
☐	ERG (electroretinogram) abnormal
☐	Retinal dystrophy or atrophy
☐	CT or MRI: brainstem atrophy or hypoplasia
☐	ACTH low in serum
☐	Follicle-stimulating hormone (FSH) low in serum

Generated by SimulConsult® on 1 May 2018 15:36 using software of 20 March 2018 12:14 and database of 24 April 2018 7:07.
Disease incidence was used. Onset was ignored. Genome used.

SimulConsult

Family 2013

Summary for a 8 year old boy with:

Pertinent positive findings

* = required to be in diseases considered; onsets can be at an age, by an age, or unknown

Req'd	Onset	Finding	Pertinence
	≤1y	Eye movement deficit, horizontal	
	≤3y	Dystonia	
	≤3y	Regression	
*	≤1y	Seizures with abnormal movements	
	≤1y	Hypotonia	
*	≤1y	Motor developmental delay	
*	≤3y	Intellectual disability	

high→

Family history

Family history based on known clinical findings

2 of 3 sisters affected
Mother not affected
Father not affected
Consanguinity: 1st cousin

Pertinent gene findings from the variant table

Severity scores shown on left, with maximum of 5, and potential compound heterozygotes marked with "C"

Severity	Finding	Pertinence
5	HACE1 gene variants (biallelic)	
2	ATP7A gene variant (X-linked)	
2	SCN2A gene variant (monoallelic)	
2	TRIO gene variant (monoallelic)	
3	MSH3 gene variant (monoallelic)	

high→

Differential diagnosis

Disease	Probability
Spastic paraplegia and psychomotor retardation with or without seizures	
Aicardi-Goutières syndrome, AR	
Creatine deficiency: GAMT creatine synthesis	
PCH1B: pontocerebellar hypoplasia, EXOSC3-related	
Peroxisomal acyl-CoA oxidase deficiency	
NBIA2B: neuroaxonal dystrophy, atypical	
Folate deficiency, cerebral	
Canavan disease, neonatal / infantile	
GM1 gangliosidosis, type II (late infantile / juvenile)	
NBIA2A: INAD: infantile neuroaxonal dystrophy, classic	

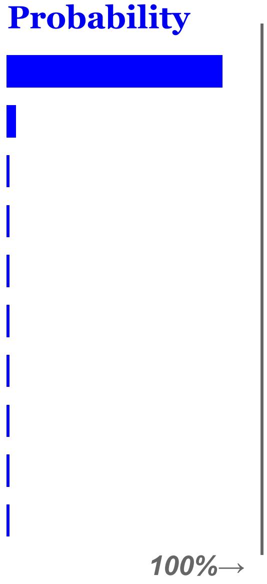

Most useful tests for this patient

Top tests ranked by usefulness in narrowing the differential, taking into account cost and treatability

Order	Test
☐	Bundle: CT scan of the brain
☐	Bundle: MRI scan of the brain
☐	WBC high in CSF
☐	X-ray or CT: brain calcifications
☐	Interferon-α in the CSF elevated

Generated by SimulConsult® on 1 May 2018 15:55 using software of 20 March 2018 12:14 and database of 24 April 2018 7:07.
Disease incidence was used. Onset was ignored. Genome used.

SimulConsult

Family 2020

Summary for a 6 year old boy with:

Pertinent positive findings

* = required to be in diseases considered; onsets can be at an age, by an age, or unknown

Req'd	Onset	Finding	Pertinence
*	≤1y	CT or MRI: lissencephaly	
*	≤3y	Intellectual disability	
	≤1y	CT or MRI: anencephaly or hydranencephaly	
*	≤Bir	Microcephaly	

high→

Pertinent negative findings

Absent	Finding	Pertinence
X	Seizures with abnormal movements	

high→

Family history

Family history based on known clinical findings

1 of 1 sisters affected
Mother not affected
Father not affected
Consanguinity: 1st cousin

Pertinent gene findings from the variant table

Severity scores shown on left, with maximum of 5, and potential compound heterozygotes marked with "C"

Severity	Finding	Pertinence
5	CENPJ gene variants (biallelic)	
3	KMT2D gene variant (monoallelic)	
2	GNAO1 gene variant (monoallelic)	
2	KCNT1 gene variant (monoallelic)	
2	GMNN gene variant (monoallelic)	

high→

Differential diagnosis

Disease	Probability
Seckel syndrome	
Microhydranencephaly	
CMV, symptomatic congenital infection	
MCPH5: microcephaly, primary AR, ASPM-related	
MCPH1: microcephaly, primary AR, MCPH1-related	
LIS6: KATNB1-related lissencephaly, AR	
MCPH17: microcephaly, primary AR, CIT-related	
HPE1: holoprosencephaly 1	
Microcephaly, short stature, and limb abnormalities	
MCPH6: microcephaly, primary AR, CENPJ-related	

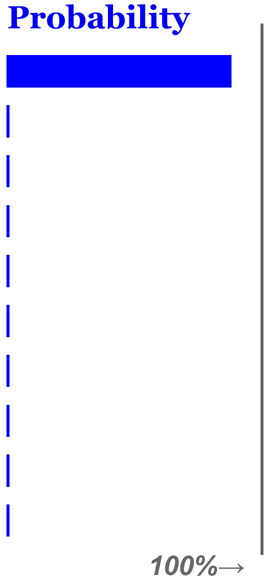

Most useful tests for this patient

Top tests ranked by usefulness in narrowing the differential, taking into account cost and treatability

Order	Test
☐	X-ray: bone age delayed
☐	X-ray: phalangeal bones deformed or hypoplastic
☐	X-ray: rib abnormality
☐	Bundle: CBC: complete blood count
☐	ATR gene variants (biallelic)

Generated by SimulConsult® on 1 May 2018 16:06 using software of 20 March 2018 12:14 and database of 24 April 2018 7:07.
Disease incidence was used. Onset was ignored. Genome used.

SimulConsult

Family 2022

Summary for a 9 year old girl with:

Pertinent positive findings

* = required to be in diseases considered; onsets can be at an age, by an age, or unknown

Req'd	Onset	Finding	Pertinence
	≤3y	Mouth: tongue rhythmic movements, abnormal	
*	≤3y	Motor developmental delay	
*	≤3y	Oculomotor apraxia	
	≤1y	Hypotonia	
*	≤3y	Intellectual disability	
*	≤3y	CT or MRI: molar tooth sign on brain imaging	
	≤1y	Hyperreflexia	
			high→

Family history

Family history based on known clinical findings

Mother not affected
Father not affected
Consanguinity: 1st cousin

Pertinent gene findings from the variant table

Severity scores shown on left, with maximum of 5, and potential compound heterozygotes marked with "C"

Severity	Finding	Pertinence
5	AHI1 gene variants (biallelic)	
5	SETD2 gene variant (monoallelic)	
2	ARID2 gene variant (monoallelic)	
5	SCNN1A gene variant (monoallelic)	
5	KRT1 gene variant (monoallelic)	
		high→

Differential diagnosis

Disease	Probability
JBTS3: Joubert syndrome, AHI1-related	
JBTS30: Joubert syndrome, ARMC9-related	
JBTS1: Joubert syndrome, INPP5E-related	
JBTS26: Joubert syndrome, KIAA0556-related	
JBTS32: Joubert syndrome, SUFU-related	
JBTS33: Joubert syndrome, PIBF1-related	
JBTS25: Joubert syndrome, CEP104-related	
JBTS28: Joubert syndrome, MKS1-related	
JBTS27: Joubert syndrome, B9D1-related	
JBTS29: Joubert syndrome, TMEM107-related	
	100%→

Most useful tests for this patient

Top tests ranked by usefulness in narrowing the differential, taking into account cost and treatability

Order	Test
☐	Blood urea nitrogen (BUN) high
☐	ERG (electroretinogram) abnormal
☐	Renal structural abnormalities
☐	Pigmentary retinopathy
☐	CT or MRI: brainstem atrophy or hypoplasia

Generated by SimulConsult® on 1 May 2018 16:16 using software of 20 March 2018 12:14 and database of 24 April 2018 7:07.
Disease incidence was used. Onset was ignored. Genome used.

SimulConsult

Family 2027

Summary for a 8 year old girl with:

Pertinent positive findings

* = required to be in diseases considered; onsets can be at an age, by an age, or unknown

Req'd	Onset	Finding	Pertinence
*	≤1y	Ataxia	
*	≤1y	Microcephaly	
*	≤1y	CT or MRI: pan-cerebellar atrophy or hypoplasia	
*	≤1y	Motor developmental delay	
*	≤3y	Intellectual disability	
			high→

Pertinent negative findings

Absent	Finding	Pertinence
X	Creatine kinase high	
		high→

Family history

Family history based on known clinical findings

1 of 1 brothers affected
Mother not affected
Father not affected
Consanguinity: 1st cousin

Pertinent gene findings from the variant table

Severity scores shown on left, with maximum of 5, and potential compound heterozygotes marked with "C"

Severity	Finding	Pertinence
5	WDR73 gene variants (biallelic)	
4	HPS3 gene variant (monoallelic)	
2	NSD1 gene variant (monoallelic)	
2C	FANCA gene variants (biallelic)	
5	PDE11A gene variant (monoallelic)	
		high→

Differential diagnosis

Disease

- Galloway-Mowat syndrome 1, WDR73-related
- CLN10: congenital neuronal ceroid lipofuscinosis
- PCH11: pontocerebellar hypoplasia, TBC1D23-related
- SCAR2: Spinocerebellar ataxia, AR, congenital nonprogressive
- CDG1A: PMM2-related
- Pitt-Hopkins syndrome
- CDG1C: ALG6-related
- PCH8: pontocerebellar hypoplasia, CHMP1A-related
- Corpus callosum agenesis, facial anomalies, cerebellar ataxia
- CCFDN

Probability

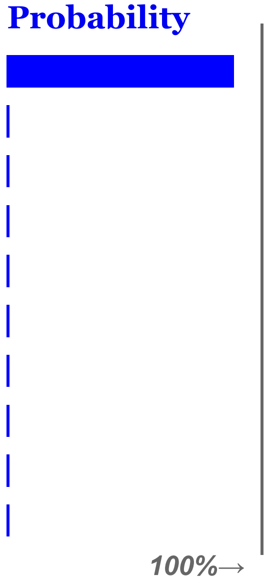

Most useful tests for this patient

Top tests ranked by usefulness in narrowing the differential, taking into account cost and treatability

Order	Test
☐	Nephrotic degree to proteinuria
☐	Bundle: UA (urinalysis)
☐	Albumin low in serum
☐	Triglycerides in serum high or false positive
☐	Bundle: Renal biopsy

Generated by SimulConsult® on 1 May 2018 16:26 using software of 20 March 2018 12:14 and database of 24 April 2018 7:07.

Disease incidence was used. Onset was ignored. Genome used.

SimulConsult

Family 2028

Summary for a 10 year old girl with:

Pertinent positive findings

* = required to be in diseases considered; onsets can be at an age, by an age, or unknown

Req'd	Onset	Finding	Pertinence
*	≤1y	CT or MRI: cerebral cortex atrophy or hypoplasia	
*	≤Bir	Microcephaly	
*	≤3y	Motor developmental delay	
*	≤3y	Intellectual disability	

high→

Pertinent negative findings

Absent	Finding	Pertinence
X	Regression	

high→

Family history

Family history based on known clinical findings

1 of 2 sisters affected
0 of 2 brothers affected
Mother not affected
Father not affected
Consanguinity: 1st cousin

Pertinent gene findings from the variant table

Severity scores shown on left, with maximum of 5, and potential compound heterozygotes marked with "C"

Severity	Finding	Pertinence
5	ASPM gene variants (biallelic)	
4	SMC3 gene variant (monoallelic)	
4	FGFR3 gene variant (monoallelic)	
2	ATP7A gene variant (X-linked)	
2	OFD1 gene variant (X-linked)	

high→

Differential diagnosis

Disease	Probability
MCPH5: microcephaly, primary AR, ASPM-related	
CMV, symptomatic congenital infection	
CLN10: congenital neuronal ceroid lipofuscinosis	
CCFDN	
Neurodevelopmental disorder with microcephaly, hypotonia, & brain anomalies	
Spastic tetraplegia, thin corpus callosum, and progressive microcephaly	
MCPH2: microcephaly, primary AR, WDR62-related	
3-phosphoglycerate dehydrogenase def., infantile	
PEHO syndrome	
Aicardi-Goutières syndrome, AR	

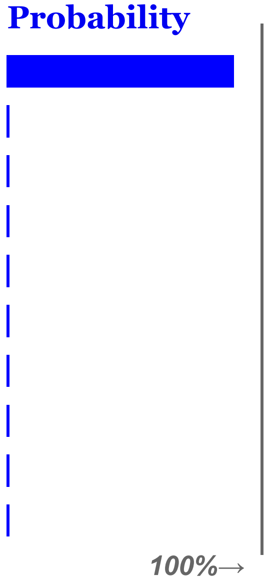

Most useful tests for this patient

Top tests ranked by usefulness in narrowing the differential, taking into account cost and treatability

Order	Test
☐	CT or MRI: lissencephaly
☐	MRI: white matter abnormality
☐	CT or MRI: pan-cerebellar atrophy or hypoplasia
☐	MRI: hypomyelination type of white matter abnormality
☐	CT or MRI: corpus callosum hypogenesis

Generated by SimulConsult® on 1 May 2018 16:33 using software of 20 March 2018 12:14 and database of 24 April 2018 7:07.
Disease incidence was used. Onset was ignored. Genome used.

SimulConsult

Family 2056

Summary for a 6 year old boy with:

Pertinent positive findings

* = required to be in diseases considered; onsets can be at an age, by an age, or unknown

Req'd	Onset	Finding	Pertinence
*	≤3y	Skin: keratotic, scaly or ichthyotic	
*	≤3y	Nystagmus, non-rotary	
*	≤1y	Motor developmental delay	
	≤1y	CT or MRI: vermal cerebellar atrophy or hypoplasia	
	≤1y	Hypotonia	
	≤3y	Intellectual disability	

high→

Family history

Family history based on known clinical findings

0 of 1 sisters affected
1 of 1 brothers affected
Mother not affected
Father not affected
Consanguinity: 3rd cousin

Pertinent gene findings from the variant table

Severity scores shown on left, with maximum of 5, and potential compound heterozygotes marked with "C"

Severity	Finding	Pertinence
5	SRD5A3 gene variants (biallelic)	
5	COL2A1 gene variant (monoallelic)	
2	LIPA gene variant (monoallelic)	
2	SLC26A4 gene variant (monoallelic)	
2	PKHD1 gene variant (monoallelic)	

high→

Differential diagnosis

Disease	Probability
CDG1Q: SRD5A3-related	
Kaufman oculocerebrofacial syndrome	
CFC1: cardiofaciocutaneous syndrome, BRAF-related	
Refsum disease, infantile	
CFC3: cardiofaciocutaneous syndrome, MAP2K1-related	
CFC4: cardiofaciocutaneous syndrome, MAP2K2-related	
Trichothiodystrophy, nonphotosensitive, AR	
Wolf-Hirschhorn syndrome	
Ichthyosis follicularis, alopecia, and photophobia	
Chanarin-Dorfman neutral lipid storage disease	

100%→

Most useful tests for this patient

Top tests ranked by usefulness in narrowing the differential, taking into account cost and treatability

Order	Test
☐	Bundle: Isoelectric focusing for transferrin glycosylation
☐	Transferrin hypoglycosylated; type 1 pattern
☐	Bundle: CBC: complete blood count
☐	Antithrombin III activity low
☐	Clotting factor deficiencies, multiple

Generated by SimulConsult® on 1 May 2018 16:50 using software of 20 March 2018 12:14 and database of 24 April 2018 7:07.
Disease incidence was used. Onset was ignored. Genome used.

SimulConsult

Family 2131

Summary for a 11 year old boy with:

Pertinent positive findings

* = required to be in diseases considered; onsets can be at an age, by an age, or unknown

Req'd	Onset	Finding	Pertinence
*	≤1y	MRI: polymicrogyria	
	≤1y	Forehead, sloping	
*	≤Bir	Microcephaly	
*	≤1y	Hyperreflexia	
*	≤1y	Hypertonia / stiffness	
*	≤1y	Motor developmental delay	
*	≤1y	Intellectual disability	
			high→

Family history

Family history based on known clinical findings

0 of 2 brothers affected
Mother not affected
Father not affected
Consanguinity: 1st cousin

Pertinent gene findings from the variant table

Severity scores shown on left, with maximum of 5, and potential compound heterozygotes marked with "C"

Severity	Finding	Pertinence
4	WDR62 gene variants (biallelic)	
5	ATP7A gene variant (X-linked)	
2	KIF21A gene variant (monoallelic)	
2	DIAPH1 gene variant (monoallelic)	
2	SCNN1B gene variant (monoallelic)	
		high→

Differential diagnosis

Disease

MCPH2: microcephaly, primary AR, WDR62-related

CMV, symptomatic congenital infection

LIS6: KATNB1-related lissencephaly, AR

LIS8: TMTC3-related lissencephaly, AR

Microcephaly, short stature, and polymicrogyria with seizures

PEHO-like syndrome, CCDC88A-related

Warburg micro syndrome

Toxoplasmosis, symptomatic congenital infection

PEHO syndrome

Menkes disease, classic

Probability

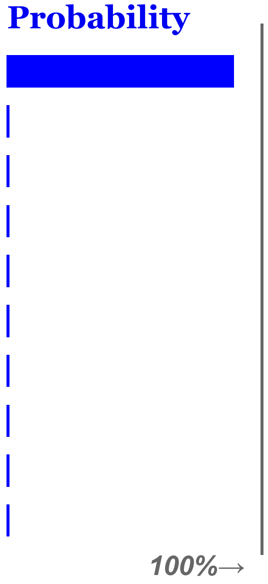

Most useful tests for this patient

Top tests ranked by usefulness in narrowing the differential, taking into account cost and treatability

Order	Test
☐	Bundle: CT scan of the brain
☐	CT or MRI: cerebral cortex atrophy or hypoplasia
☐	CT or MRI: pan-cerebellar atrophy or hypoplasia
☐	Bundle: CBC: complete blood count
☐	CT or MRI: lissencephaly

Generated by SimulConsult® on 1 May 2018 16:59 using software of 20 March 2018 12:14 and database of 24 April 2018 7:07.

Disease incidence was used. Onset was ignored. Genome used.

SimulConsult

Family 2132

Summary for a 14 year old girl with:

Pertinent positive findings

* = required to be in diseases considered; onsets can be at an age, by an age, or unknown

Req'd	Onset	Finding	Pertinence
*	≤3y	Myoclonus	
	≤3y	Weakness, significant	
*	≤3y	Gait disturbance	
	≤3y	Spasticity character to hypertonia	
*	≤6y	Regression	
*	≤3y	Hypertonia / stiffness	
	≤3y	Motor developmental delay	
			high→

Pertinent negative findings

Absent	Finding	Pertinence
X	Microcephaly	
X	Dysarthria or abnormal sound character	
X	Creatine kinase high	
		high→

Family history

Family history based on known clinical findings

1 of 1 sisters affected
Mother not affected
Father not affected
Consanguinity: From same ethnic group

Pertinent gene findings from the variant table

Severity scores shown on left, with maximum of 5, and potential compound heterozygotes marked with "C"

Severity	Finding	Pertinence
5	RNASEH2B gene variants (biallelic)	
5	ATP7A gene variant (X-linked)	
2C	IFT74 gene variants (biallelic)	
2	COL11A2 gene variant (monoallelic)	
2	PDE3A gene variant (monoallelic)	
		high→

Differential diagnosis

Disease	Probability
Aicardi-Goutières syndrome, AR	
CLN7: neuronal ceroid lipofuscinosis, Gypsy variant	
CLN8: neuronal ceroid lipofuscinosis, Turkish variant	
NAGA deficiency I: Schindler disease type I	
CLN1 (INCL): infantile neuronal ceroid lipofuscinosis	
CLN5: neuronal ceroid lipofuscinosis, Finnish variant	
CLN6: neuronal ceroid lipofuscinosis, Indian variant	
NBIA2A: INAD: infantile neuroaxonal dystrophy, classic	
Hyperornithinemia-hyperammonemia-homocitrullinuria syndrome	
CLN3: juvenile neuronal ceroid lipofuscinosis	

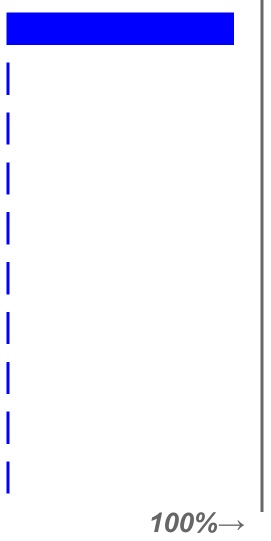

Most useful tests for this patient

Top tests ranked by usefulness in narrowing the differential, taking into account cost and treatability

Order	Test
☐	WBC high in CSF
☐	Interferon-α in the CSF elevated
☐	X-ray or CT: brain calcifications
☐	Bundle: CT scan of the brain
☐	Bundle: MR spectroscopy

Generated by SimulConsult® on 1 May 2018 17:07 using software of 20 March 2018 12:14 and database of 24 April 2018 7:07.
Disease incidence was used. Onset was ignored. Genome used.

SimulConsult

Family 2236

Summary for a 10 year old boy with:

Pertinent positive findings

* = required to be in diseases considered; onsets can be at an age, by an age, or unknown

Req'd	Onset	Finding	Pertinence
*	≤3m	Cataracts	
*	≤1y	Hypogonadism or cryptorchidism	
*	≤1y	CT or MRI: corpus callosum hypogenesis	
	≤Bir	Mouth: palate cleft	
*	≤1y	CT or MRI: cerebral cortex atrophy or hypoplasia	
*	≤3y	Intellectual disability	

high→

Family history

Family history based on known clinical findings

0 of 1 sisters affected
Mother not affected
Father not affected
Consanguinity: 1st cousin

Pertinent gene findings from the variant table

Severity scores shown on left, with maximum of 5, and potential compound heterozygotes marked with "C"

Severity	Finding	Pertinence
5	RAB3GAP1 gene variants (biallelic)	
2C	APOB gene variants (biallelic)	
5	ZNF423 gene variant (monoallelic)	
2	FUS gene variant (monoallelic)	
3	HMCN1 gene variant (monoallelic)	

high→

Differential diagnosis

Disease	Probability
Warburg micro syndrome	
Chromosome 1p36 deletion syndrome	
Zellweger syndrome	
Muscular dystrophy-dystroglycanopathy A4	
Chromosome 22q11.2 deletion syndrome	
Jacobsen syndrome	
Chromosome 2q31.1 deletion syndrome	
Pallister-Killian syndrome	
Yunis-Varon syndrome	
Seckel syndrome	
	100%→

Most useful tests for this patient

Top tests ranked by usefulness in narrowing the differential, taking into account cost and treatability

Order	Test
☐	Microcornea: flat or protruding
☐	Retinal dystrophy or atrophy
☐	CT or MRI: pan-cerebellar atrophy or hypoplasia
☐	Bundle: Echocardiogram
☐	Imaging: ventricular septal defect

Generated by SimulConsult® on 1 May 2018 17:20 using software of 20 March 2018 12:14 and database of 24 April 2018 7:07.

Disease incidence was used. Onset was ignored. Genome used.

SimulConsult

Family 2245

Summary for a 7 year old boy with:

Pertinent positive findings

* = required to be in diseases considered; onsets can be at an age, by an age, or unknown

Req'd	Onset	Finding	Pertinence
*	≤3y	Motor developmental delay	
*	≤3y	MRI: polymicrogyria	
*	≤3y	Intellectual disability	

high→

Family history

Family history based on known clinical findings

0 of 2 sisters affected
Mother not affected
Father not affected
Consanguinity: 1st cousin

Pertinent gene findings from the variant table

Severity scores shown on left, with maximum of 5, and potential compound heterozygotes marked with "C"

Severity	Finding	Pertinence
5	ADGRG1 gene variants (biallelic)	
5C	PLCB1 gene variants (biallelic)	
3C	COL18A1 gene variants (biallelic)	
4	MSTO1 gene variant (monoallelic)	
3	TSC2 gene variant (monoallelic)	

high→

Differential diagnosis

Disease	Probability
Polymicrogyria, bilateral frontoparietal	
CMV, symptomatic congenital infection	
Muscular dystrophy-dystroglycanopathy A4	
Toxoplasmosis, symptomatic congenital infection	
Chromosome 22q11.2 deletion syndrome	
Hypomelanosis of Ito	
EIEE12: epileptic encephalopathy, early infantile, PLCB1-related	
Complex cortical dysplasia with other brain malformations 8	
MCPH2: microcephaly, primary AR, WDR62-related	
Zellweger syndrome	

100%→

Most useful tests for this patient

Top tests ranked by usefulness in narrowing the differential, taking into account cost and treatability

Order	Test
☐	Bundle: CT scan of the brain
☐	CT or MRI: pan-cerebellar atrophy or hypoplasia
☐	CT, MRI or head USG: hydrocephalus, not ex-vacuo
☐	Bundle: MRI of the spine
☐	MRI: white matter abnormality

Generated by SimulConsult® on 1 May 2018 17:26 using software of 20 March 2018 12:14 and database of 24 April 2018 7:07.

Disease incidence was used. Onset was ignored. Genome used.

SimulConsult

Family 2247

Summary for a 8 year old girl with:

Pertinent positive findings

* = required to be in diseases considered; onsets can be at an age, by an age, or unknown

Req'd	Onset	Finding	Pertinence
	≤3y	Muscular atrophy or hypoplasia	
*	≤3y	Basal ganglia nature to brain calcifications	
*	≤3y	Motor developmental delay	
*	≤3y	Intellectual disability	
*	≤3y	Microcephaly	
	≤3y	Stature short	
			high→

Family history

Family history based on known clinical findings

1 of 1 sisters affected
Mother not affected
Father not affected
Consanguinity: 1st cousin

Pertinent gene findings from the variant table

Severity scores shown on left, with maximum of 5, and potential compound heterozygotes marked with "C"

Severity	Finding	Pertinence
5	ERCC8 gene variants (biallelic)	
5	ERCC8 gene variant (monoallelic)	
3	DYNC1H1 gene variant (monoallelic)	
5	PDCD10 gene variant (monoallelic)	
5C	ABCG5 gene variants (biallelic)	
		high→

Differential diagnosis

Disease	Probability
Cockayne syndrome I, moderate or classic	
Cockayne syndrome II, severe or early-onset	
CMV, symptomatic congenital infection	
Phenylketonuria	
Varicella, symptomatic congenital infection	
Toxoplasmosis, symptomatic congenital infection	
Aicardi-Goutières syndrome, AR	
Hyperornithinemia-hyperammonemia-homocitrullinuria syndrome	
MTDPS4A: POLG-related mtDNA depletion	
Trisomy 21 (Down syndrome)	

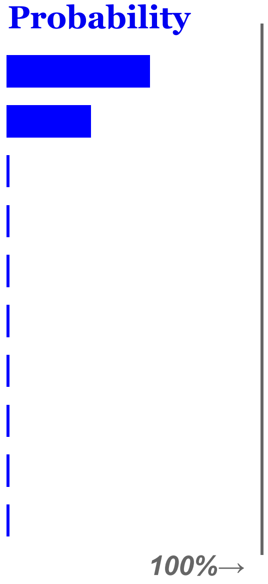

Most useful tests for this patient

Top tests ranked by usefulness in narrowing the differential, taking into account cost and treatability

Order	Test
☐	Bundle: MRI scan of the brain
☐	Chest X-ray or CT: infiltrate or consolidation
☐	ERG (electroretinogram) abnormal
☐	CT, MRI or head USG: hydrocephalus, not ex-vacuo
☐	MRI: white matter abnormality

Generated by SimulConsult® on 1 May 2018 21:49 using software of 20 March 2018 12:14 and database of 24 April 2018 7:07.
Disease incidence was used. Onset was ignored. Genome used.

SimulConsult

Family 2248

Summary for a 12 year old boy with:

Pertinent positive findings

* = required to be in diseases considered; onsets can be at an age, by an age, or unknown

Req'd	Onset	Finding	Pertinence
	≤1y	CT or MRI: basal ganglia abnormalities	
*	≤1y	Microcephaly	
*	≤3y	CT or MRI: corpus callosum hypogenesis	
	≤6y	Intellectual disability	
*	≤3y	MRI: white matter abnormality	
	≤3y	Spasticity character to hypertonia	
*	≤1y	Hyperreflexia	
*	≤6y	Motor developmental delay	
	≤6y	Teeth: macrodontia	
			high→

Family history

Family history based on known clinical findings

1 of 2 sisters affected
Mother not affected
Father not affected
Consanguinity: 1st cousin

Pertinent gene findings from the variant table

Severity scores shown on left, with maximum of 5, and potential compound heterozygotes marked with "C"

Severity	Finding	Pertinence
5	MCOLN1 gene variants (biallelic)	
4	RYR2 gene variant (monoallelic)	
4	MYH3 gene variant (monoallelic)	
5	VANGL1 gene variant (monoallelic)	
5	DSP gene variant (monoallelic)	
		high→

Differential diagnosis

Disease	Probability
Mucolipidosis IV, typical form	
COXPD1: combined OXPHOS deficiency, GFM1-related	
LISX2: ARX-related lissencephaly, X-linked	
Spastic tetraplegia, thin corpus callosum, and progressive microcephaly	
HLD12: leukodystrophy, hypomyelinating, VPS11-related	
PCH9: pontocerebellar hypoplasia, AMPD2	
Neurodevelopmental disorder with progressive microcephaly, spasticity, and brain anomalies	
PCH8: pontocerebellar hypoplasia, CHMP1A-related	
Pyruvate dehydrogenase E1α deficiency, late infantile	
Neurodevelopmental disorder with microcephaly, hypotonia, & brain anomalies	

100%→

Most useful tests for this patient

Top tests ranked by usefulness in narrowing the differential, taking into account cost and treatability

Order	Test
☐	Achlorhydria
☐	Pigmentary retinopathy
☐	Gastrin in serum elevated
☐	Bundle: Conjunctival biopsy
☐	Conjunctival biopsy: multilaminate bodies in epithelial and endothelial cells

Generated by SimulConsult® on 2 May 2018 17:18 using software of 20 March 2018 12:14 and database of 24 April 2018 7:07.

Disease incidence was used. Onset was ignored. Genome used.

SimulConsult

Family 2282

Summary for a 10 year old boy with:

Pertinent positive findings

* = required to be in diseases considered; onsets can be at an age, by an age, or unknown

Req'd	Onset	Finding	Pertinence
	≤3y	Forehead, sloping	
*	≤3y	CT or MRI: cerebral cortex atrophy or hypoplasia	
*	≤Bir	Microcephaly	
	≤Bir	Mouth: palate cleft	
	≤Bir	Lip: cleft	
*	≤3y	Motor developmental delay	
*	≤3y	Intellectual disability	
			high→

Family history

Family history based on known clinical findings

1 of 4 maternal uncles affected
1 of 2 sisters affected
Mother not affected
Father not affected
Consanguinity: 1st cousin

Pertinent gene findings from the variant table

Severity scores shown on left, with maximum of 5, and potential compound heterozygotes marked with "C"

Severity	Finding	Pertinence
5	ASPM gene variants (biallelic)	
2	TELO2 gene variants (biallelic)	
4	SERAC1 gene variants (biallelic)	
3	PUF60 gene variant (monoallelic)	
3	FOXH1 gene variant (monoallelic)	
		high→

Differential diagnosis

Disease	Probability
MCPH5: microcephaly, primary AR, ASPM-related	
Vici syndrome	
Nijmegen breakage syndrome	
Muscular dystrophy-dystroglycanopathy A4	
Yunis-Varon syndrome	
Opitz G/BBB syndrome, type I, X-linked	
Wolf-Hirschhorn syndrome	
Chromosome 22q11.2 deletion syndrome	
Chromosome 1p36 deletion syndrome	
Toriello-Carey syndrome	

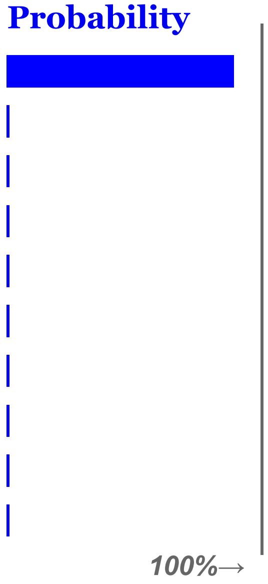

Most useful tests for this patient

Top tests ranked by usefulness in narrowing the differential, taking into account cost and treatability

Order	Test
☐	CT or MRI: lissencephaly
☐	Creatine kinase high
☐	Bundle: Echocardiogram
☐	Renal structural abnormalities
☐	Eye: anterior chamber abnormalities

Generated by SimulConsult® on 30 April 2018 10:23 using software of 20 March 2018 12:14 and database of 24 April 2018 7:07.
Disease incidence was used. Onset was ignored. Genome used.

SimulConsult

Family 2303

Summary for a 12 year old boy with:

Pertinent positive findings

* = required to be in diseases considered; onsets can be at an age, by an age, or unknown

Req'd	Onset	Finding	Pertinence
*	≤3y	Contractures or passive limited range of motion	
*	≤3y	Cataracts	
*	≤3y	Microcephaly	
	≤3y	CT or MRI: corpus callosum hypogenesis	
*	≤3y	Hypogonadism or cryptorchidism	
*	≤3y	Intellectual disability	
*	≤3y	Motor developmental delay	
			high→

Family history

Family history based on known clinical findings

0 of 2 sisters affected
1 of 2 brothers affected
Mother not affected
Father not affected

Pertinent gene findings from the variant table

Severity scores shown on left, with maximum of 5, and potential compound heterozygotes marked with "C"

Severity	Finding	Pertinence
5	RAB3GAP1 gene variants (biallelic)	
3	CHD7 gene variant (monoallelic)	
4	ARID1B gene variant (monoallelic)	
2	ACTB gene variant (monoallelic)	
2	ATRX gene variant (X-linked)	
		high→

Differential diagnosis

Disease	Probability
Warburg micro syndrome	
CHARGE syndrome	
Zellweger syndrome	
Muscular dystrophy-dystroglycanopathy A4	
Smith-Lemli-Opitz syndrome	
Rubinstein-Taybi syndrome	
Peters plus syndrome	
Chromosome 22q11.2 deletion syndrome	
Muscular dystrophy-dystroglycanopathy A1	
Chromosome 1p36 deletion syndrome	

100%→

Most useful tests for this patient

Top tests ranked by usefulness in narrowing the differential, taking into account cost and treatability

Order	Test
☐	Microcornea: flat or protruding
☐	Retinal dystrophy or atrophy
☐	Eye: anterior chamber abnormalities
☐	CT or MRI: pan-cerebellar atrophy or hypoplasia
☐	Bundle: Echocardiogram

Generated by SimulConsult® on 2 May 2018 10:39 using software of 20 March 2018 12:14 and database of 24 April 2018 7:07.

Disease incidence was used. Onset was ignored. Genome used.

SimulConsult

Family 2421

Summary for a 13 year old girl with:

Pertinent positive findings

* = required to be in diseases considered; onsets can be at an age, by an age, or unknown

Req'd	Onset	Finding	Pertinence
*	≤3y	Cataracts	
*	≤3y	CT or MRI: corpus callosum hypogenesis	
*	≤3y	CT or MRI: pan-cerebellar atrophy or hypoplasia	
*	≤3y	Microcephaly	
*	≤3y	Motor developmental delay	
*	≤3y	Intellectual disability	
			high→

Family history

Family history based on known clinical findings

1 of 1 sisters affected
Mother not affected
Father not affected
Consanguinity: 2nd cousin

Pertinent gene findings from the variant table

Severity scores shown on left, with maximum of 5, and potential compound heterozygotes marked with "C"

Severity	Finding	Pertinence
5	TBC1D20 gene variants (biallelic)	
4	SMARCA4 gene variant (monoallelic)	
2	CHD7 gene variant (monoallelic)	
3	KANSL1 gene variant (monoallelic)	
2	RNF113A gene variant (X-linked)	
		high→

Differential diagnosis

Disease	Probability
Warburg micro syndrome	
Coffin-Siris syndrome	
Neurodevelopmental disorder with microcephaly, hypotonia, & brain anomalies	
MCPH10: microcephaly, primary AR, ZNF335-related	
Mosaic variegated aneuploidy syndrome 1	
Smith-Lemli-Opitz syndrome	
Muscular dystrophy-dystroglycanopathy A4	
Peters plus syndrome	
MTDPS13: FBXL4 encephalomyopathic mtDNA depletion syndrome	
CHARGE syndrome	

100%→

Most useful tests for this patient

Top tests ranked by usefulness in narrowing the differential, taking into account cost and treatability

Order	Test
☐	Microcornea: flat or protruding
☐	RAB3GAP1 gene variants (biallelic)
☐	Retinal dystrophy or atrophy
☐	Creatine kinase high
☐	Eye: anterior chamber abnormalities

Generated by SimulConsult® on 2 May 2018 11:17 using software of 20 March 2018 12:14 and database of 24 April 2018 7:07.

Disease incidence was used. Onset was ignored. Genome used.

SimulConsult

Family 2424

Summary for a 7 year old boy with:

Pertinent positive findings

* = required to be in diseases considered; onsets can be at an age, by an age, or unknown

Req'd	Onset	Finding	Pertinence
*	≤3y	EEG: spikes, focal	
*	≤3y	Optic atrophy or hypoplasia	
*	≤3y	Seizures with abnormal movements	
	≤1y	ERG (electroretinogram) abnormal	
*	≤Bir	Microcephaly	
	≤3y	Visual impairment despite lens correction	
	≤1y	Hypotonia	
*	≤3y	Intellectual disability	
*	≤1y	Motor developmental delay	

high→

Family history

Family history based on known clinical findings

1 of 1 brothers affected
Mother not affected
Father not affected
Consanguinity: 1st cousin

Pertinent gene findings from the variant table

Severity scores shown on left, with maximum of 5, and potential compound heterozygotes marked with "C"

Severity	Finding	Pertinence
5	ST3GAL5 gene variants (biallelic)	
5	HESX1 gene variant (monoallelic)	
2	MAP2K1 gene variant (monoallelic)	
2	PCDH12 gene variants (biallelic)	
2C	HERC2 gene variants (biallelic)	

high→

Differential diagnosis

Disease

Salt and pepper developmental regression syndrome

Cohen syndrome

Zellweger syndrome

Angelman syndrome

CLN1 (INCL): infantile neuronal ceroid lipofuscinosis

Microcephaly-capillary malformation syndrome

Muscular dystrophy-dystroglycanopathy A1

EIEE58: early infantile epileptic encephalopathy, NTRK2-related

Muscular dystrophy-dystroglycanopathy A3

CLN2: late-infantile neuronal ceroid lipofuscinosis

Probability

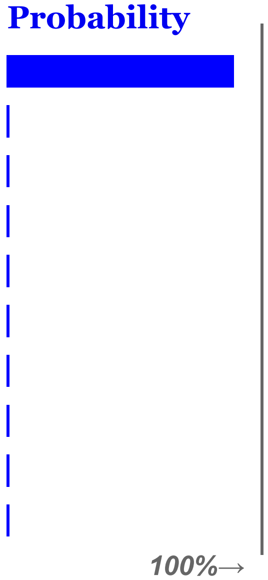

100%→

Most useful tests for this patient

Top tests ranked by usefulness in narrowing the differential, taking into account cost and treatability

Order	Test
☐	Bundle: MRI scan of the brain
☐	Bundle: CT scan of the brain
☐	Pigmentary retinopathy
☐	MRI: white matter abnormality
☐	EEG: slowing, generalized

Generated by SimulConsult® on 2 May 2018 11:21 using software of 20 March 2018 12:14 and database of 24 April 2018 7:07.

Disease incidence was used. Onset was ignored. Genome used.

Family 2450

Summary for a 12 year old girl with:

Pertinent positive findings

* = required to be in diseases considered; onsets can be at an age, by an age, or unknown

Req'd	Onset	Finding	Pertinence
*	≤3y	EMG: myopathic changes	
	≤6y	Scoliosis with or without kyphosis	
*	≤3y	Microcephaly	
*	≤1y	Cataracts	
*	≤3y	Hypotonia	
*	≤3y	Intellectual disability	
*	≤3y	Motor developmental delay	
	≤6y	Nystagmus, rotary	
			high→

Family history

Family history based on known clinical findings

1 of 1 brothers affected
Mother not affected
Father not affected
Consanguinity: 1st cousin

Pertinent gene findings from the variant table

Severity scores shown on left, with maximum of 5, and potential compound heterozygotes marked with "C"

Severity	Finding	Pertinence
4	GMPPB gene variants (biallelic)	
4	TSHZ1 gene variant (monoallelic)	
4C	FAT4 gene variants (biallelic)	
4	TTN gene variant (monoallelic)	
5C	NPHP3 gene variants (biallelic)	
		high→

Differential diagnosis

Disease	Probability
Muscular dystrophy-dystroglycanopathy A14	
Muscular dystrophy-dystroglycanopathy A4	
Muscular dystrophy-dystroglycanopathy A2	
Muscular dystrophy-dystroglycanopathy A1	
Muscular dystrophy-dystroglycanopathy A7	
Muscular dystrophy-dystroglycanopathy A10	
Muscular dystrophy-dystroglycanopathy A3	
Muscular dystrophy-dystroglycanopathy A12	
Muscular dystrophy-dystroglycanopathy A11	
Muscular dystrophy-dystroglycanopathy A6	

100%→

Most useful tests for this patient

Top tests ranked by usefulness in narrowing the differential, taking into account cost and treatability

Order	Test
☐	Bundle: MRI scan of the brain
☐	Bundle: CT scan of the brain
☐	CT or MRI: pontine atrophy or hypoplasia
☐	FKTN gene variants (biallelic)
☐	CT or MRI: cerebral cortex atrophy or hypoplasia

Generated by SimulConsult® on 2 May 2018 11:31 using software of 20 March 2018 12:14 and database of 24 April 2018 7:07.

Disease incidence was used. Onset was ignored. Genome used.

SimulConsult

Family 2566

Summary for a 6 year old boy with:

Pertinent positive findings

* = required to be in diseases considered; onsets can be at an age, by an age, or unknown

Req'd	Onset	Finding	Pertinence
	≤1y	Face, triangular	
	≤1y	Ears large	
*	≤1y	Hyperreflexia	
	≤1y	Philtrum long	
*	≤3y	Microcephaly	
	≤1y	Weight low or weight loss	
*	≤3y	Motor developmental delay	
*	≤3y	Intellectual disability	
	≤1y	Ears low-set	
*	≤1y	Hypotonia	

high→

Family history

Family history based on known clinical findings

0 of 1 brothers affected
Mother not affected
Father not affected
Consanguinity: 2nd cousin

Pertinent gene findings from the variant table

Severity scores shown on left, with maximum of 5, and potential compound heterozygotes marked with "C"

Severity	Finding	Pertinence
5	PYCR2 gene variants (biallelic)	
4	ELN gene variant (monoallelic)	
4	RAI1 gene variant (monoallelic)	
2	UBE3A gene variant (monoallelic)	
2	KMT2D gene variant (monoallelic)	

high→

Differential diagnosis

Disease	Probability
HLD10: leukodystrophy, hypomyelinating, PYCR2-related	
Williams syndrome	
Chromosome 3pter-p25 deletion syndrome	
IHPRF1: infantile hypotonia, psychomotor retardation, characteristic facies	
Chromosome 14q11-q22 deletion syndrome	
PCH3: pontocerebellar hypoplasia 3	
IHPRF2: infantile hypotonia, psychomotor retardation, characteristic facies	
ADCL3: cutis laxa, AD, ALDH18A1-related	
Intellectual developmental disorder, dysmorphic facies, seizures, distal limb anomalies	
MEHMO syndrome	

100%→

Most useful tests for this patient

Top tests ranked by usefulness in narrowing the differential, taking into account cost and treatability

Order	Test
☐	Bundle: MRI scan of the brain
☐	Bundle: CT scan of the brain
☐	MRI: white matter abnormality
☐	MRI: hypomyelination type of white matter abnormality
☐	CT or MRI: pan-cerebellar atrophy or hypoplasia

Generated by SimulConsult® on 2 May 2018 12:40 using software of 20 March 2018 12:14 and database of 24 April 2018 7:07.
Disease incidence was used. Onset was ignored. Genome used.

SimulConsult

Family 2641

Summary for a 6 year old girl with:

Pertinent positive findings

* = required to be in diseases considered; onsets can be at an age, by an age, or unknown

Req'd	Onset	Finding	Pertinence
	≤3y	Mouth: tongue rhythmic movements, abnormal	
*	≤3y	Oculomotor apraxia	
*	≤3y	Intellectual disability	
*	≤3y	Motor developmental delay	
	≤3y	Hypotonia	
	≤3y	CT or MRI: vermal cerebellar atrophy or hypoplasia	
*	≤3y	CT or MRI: molar tooth sign on brain imaging	
	≤3y	Hyporeflexia	

high→

Family history

Family history based on known clinical findings

0 of 1 sisters affected
0 of 1 brothers affected
Mother not affected
Father not affected
Consanguinity: 1st cousin

Pertinent gene findings from the variant table

Severity scores shown on left, with maximum of 5, and potential compound heterozygotes marked with "C"

Severity	Finding	Pertinence
4	TMEM138 gene variants (biallelic)	
4	SCN8A gene variant (monoallelic)	
3	KAT6B gene variant (monoallelic)	
2	BRWD3 gene variant (X-linked)	
2	CRYGB gene variant (monoallelic)	

high→

Differential diagnosis

Disease — **Probability**

- JBTS16: Joubert syndrome, TMEM138-related
- JBTS30: Joubert syndrome, ARMC9-related
- JBTS1: Joubert syndrome, INPP5E-related
- JBTS26: Joubert syndrome, KIAA0556-related
- JBTS32: Joubert syndrome, SUFU-related
- JBTS25: Joubert syndrome, CEP104-related
- JBTS29: Joubert syndrome, TMEM107-related
- JBTS27: Joubert syndrome, B9D1-related
- JBTS28: Joubert syndrome, MKS1-related
- JBTS34: Joubert syndrome, B9D2-related
- JBTS31: Joubert syndrome, CEP120-related
- JBTS33: Joubert syndrome, PIBF1-related

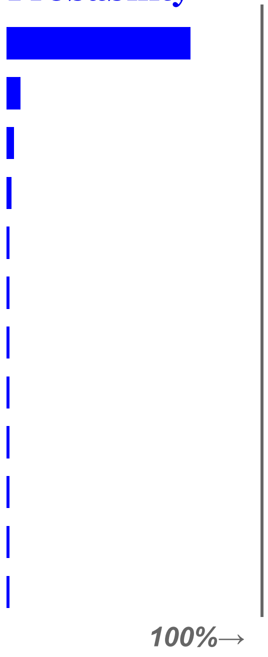

Most useful tests for this patient

Top tests ranked by usefulness in narrowing the differential, taking into account cost and treatability

Order	Test
☐	ERG (electroretinogram) abnormal
☐	Blood urea nitrogen (BUN) high
☐	CT or MRI: brainstem atrophy or hypoplasia
☐	Renal structural abnormalities
☐	ARMC9 gene variants (biallelic)

Generated by SimulConsult® on 2 May 2018 12:51 using software of 20 March 2018 12:14 and database of 24 April 2018 7:07.
Disease incidence was used. Onset was ignored. Genome used.

SimulConsult

Family 2643

Summary for a 8 year old boy with:

Pertinent positive findings

* = required to be in diseases considered; onsets can be at an age, by an age, or unknown

Req'd	Onset	Finding	Pertinence
	≤3y	Contractures or passive limited range of motion	
*	≤3y	MRI: white matter abnormality	
*	≤3y	CT or MRI: cerebral cortex atrophy or hypoplasia	
*	≤3y	CT or MRI: corpus callosum hypogenesis	
*	≤3y	Microcephaly	
	≤3y	Hypertonia / stiffness	
*	≤3y	Spasticity character to hypertonia	
	≤3y	Hyperreflexia	
*	≤3y	Intellectual disability	
*	≤3y	Motor developmental delay	
			high→

Family history

Family history based on known clinical findings

1 of 1 brothers affected
Mother not affected
Father not affected
Consanguinity: 1st cousin

Pertinent gene findings from the variant table

Severity scores shown on left, with maximum of 5, and potential compound heterozygotes marked with "C"

Severity	Finding	Pertinence
5	SLC1A4 gene variants (biallelic)	
2	GALT gene variant (monoallelic)	
2	CCDC50 gene variant (monoallelic)	
2	HBG2 gene variant (monoallelic)	
2	ADCY5 gene variant (monoallelic)	
		high→

Differential diagnosis

Disease	Probability
Spastic tetraplegia, thin corpus callosum, and progressive microcephaly	
Neurodevelopmental disorder with progressive microcephaly, spasticity, and brain anomalies	
Neurodevelopmental disorder with microcephaly, hypotonia, & brain anomalies	
Encephalopathy, progressive, early-onset with brain atrophy and thin corpus callosum	
PCH9: pontocerebellar hypoplasia, AMPD2	
Isolated sulfite oxidase deficiency, classic early-onset	
MCPH10: microcephaly, primary AR, ZNF335-related	
Webb-Dattani syndrome	
Galloway-Mowat syndrome 3, OSGEP-related	
PCH10: pontocerebellar hypoplasia, CLP1-related	

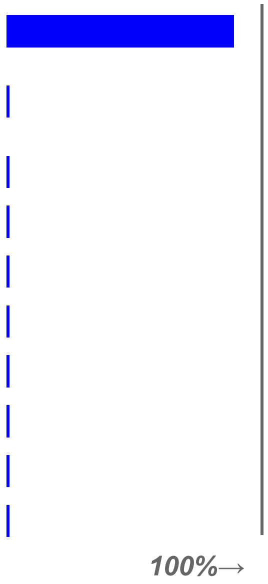

Most useful tests for this patient

Top tests ranked by usefulness in narrowing the differential, taking into account cost and treatability

Order	Test
☐	X-ray, CT or MRI: joint damage or dysplasia
☐	Bundle: Joint imaging
☐	CT or MRI: pan-cerebellar atrophy or hypoplasia
☐	MRI: hypomyelination type of white matter abnormality
☐	Bundle: EEG (electroencephalogram)

Generated by SimulConsult® on 18 May 2018 15:40 using software of 15 May 2018 09:43 and database of 15 May 2018 7:25.

Disease incidence was used. Onset was ignored. Genome used.

SimulConsult

Family 2664

Summary for a 7 year old girl with:

Pertinent positive findings

* = required to be in diseases considered; onsets can be at an age, by an age, or unknown

Req'd	Onset	Finding	Pertinence
	≤3y	Muscular atrophy or hypoplasia	
	≤1y	Face, triangular	
*	≤1y	CT or MRI: corpus callosum hypogenesis	
*	≤Bir	Microcephaly	
	≤1y	Hypotonia	
*	≤3y	Intellectual disability	
*	≤3y	Motor developmental delay	
	≤3y	Teeth: macrodontia	
			high→

Family history

Family history based on known clinical findings

Mother not affected
Father not affected
Consanguinity: 1st cousin

Pertinent gene findings from the variant table

Severity scores shown on left, with maximum of 5, and potential compound heterozygotes marked with "C"

Severity	Finding	Pertinence
5	PYCR2 gene variants (biallelic)	
3	TAF1 gene variant (X-linked)	
4	NOTCH1 gene variant (monoallelic)	
4	FBLN1 gene variant (monoallelic)	
4	MSH6 gene variant (monoallelic)	
		high→

Differential diagnosis

Disease | **Probability**

- HLD10: leukodystrophy, hypomyelinating, PYCR2-related
- IHPRF2: infantile hypotonia, psychomotor retardation, characteristic facies
- Chromosome 1p36 deletion syndrome
- Wolf-Hirschhorn syndrome
- Encephalopathy, progressive, early-onset with brain atrophy and thin corpus callosum
- Neurodevelopmental disorder with microcephaly, hypotonia, & brain anomalies
- Muscular dystrophy-dystroglycanopathy B1
- Chromosome 22q11.2 deletion syndrome
- Muscular dystrophy-dystroglycanopathy A4
- Vici syndrome

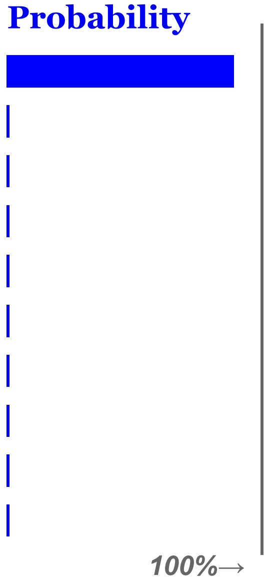

Most useful tests for this patient

Top tests ranked by usefulness in narrowing the differential, taking into account cost and treatability

Order	Test
☐	MRI: white matter abnormality
☐	MRI: hypomyelination type of white matter abnormality
☐	CT or MRI: pan-cerebellar atrophy or hypoplasia
☐	CT or MRI: cerebral cortex atrophy or hypoplasia
☐	CT or MRI: brainstem atrophy or hypoplasia

Generated by SimulConsult® on 2 May 2018 13:15 using software of 20 March 2018 12:14 and database of 24 April 2018 7:07.
Disease incidence was used. Onset was ignored. Genome used.

SimulConsult

Family 2715

Summary for a 10 year old boy with:

Pertinent positive findings

* = required to be in diseases considered; onsets can be at an age, by an age, or unknown

Req'd	Onset	Finding	Pertinence
*	≤3y	CT or MRI: lissencephaly	
*	≤3y	MRI: polymicrogyria	
*	≤3y	Microcephaly	
*	≤3y	Seizures with abnormal movements	
*	≤3y	Motor developmental delay	
*	≤3y	Intellectual disability	
			high→

Family history

Family history based on known clinical findings

0 of 1 brothers affected
Mother not affected
Father not affected
Consanguinity: 1st cousin

Pertinent gene findings from the variant table

Severity scores shown on left, with maximum of 5, and potential compound heterozygotes marked with "C"

Severity	Finding	Pertinence
5	POMGNT1 gene variants (biallelic)	
4	KCNT1 gene variant (monoallelic)	
4	MBD5 gene variant (monoallelic)	
2	ERCC6 gene variant (monoallelic)	
2	EFEMP1 gene variant (monoallelic)	
		high→

Differential diagnosis

Disease	Probability
Muscular dystrophy-dystroglycanopathy A3	
Muscular dystrophy-dystroglycanopathy A4	
MCPH2: microcephaly, primary AR, WDR62-related	
PEHO-like syndrome, CCDC88A-related	
LIS8: TMTC3-related lissencephaly, AR	
PEHO syndrome	
LIS6: KATNB1-related lissencephaly, AR	
CMV, symptomatic congenital infection	
Zellweger syndrome	
Microcephaly, short stature, and polymicrogyria with seizures	
	100%→

Most useful tests for this patient

Top tests ranked by usefulness in narrowing the differential, taking into account cost and treatability

Order	Test
☐	Creatine kinase high
☐	Eye: anterior chamber abnormalities
☐	EMG: myopathic changes
☐	Bundle: EMG (electromyogram)
☐	CT, MRI or head USG: hydrocephalus, not ex-vacuo

Generated by SimulConsult® on 2 May 2018 13:45 using software of 20 March 2018 12:14 and database of 24 April 2018 7:07.
Disease incidence was used. Onset was ignored. Genome used.

SimulConsult

Family 2730

Summary for a 9 year old girl with:

Pertinent positive findings

* = required to be in diseases considered; onsets can be at an age, by an age, or unknown

Req'd	Onset	Finding	Pertinence
*	≤6y	CT or MRI: pan-cerebellar atrophy or hypoplasia	
*	≤6y	Regression	
	≤6y	Ataxia	
*	≤6y	CT or MRI: cerebral cortex atrophy or hypoplasia	
*	≤6y	Motor developmental delay	
*	≤6y	Intellectual disability	
	≤6y	Hypotonia	
			high→

Family history

Family history based on known clinical findings

1 of 1 brothers affected
Mother not affected
Father not affected
Consanguinity: From same village

Pertinent gene findings from the variant table

Severity scores shown on left, with maximum of 5, and potential compound heterozygotes marked with "C"

Severity	Finding	Pertinence
5C	TPP1 gene variants (biallelic)	
5	KIF5C gene variant (monoallelic)	
3	CASK gene variant (X-linked)	
3	SPECC1L gene variant (monoallelic)	
3	ADAR gene variant (monoallelic)	
		high→

Differential diagnosis

Disease	Probability
CLN2: late-infantile neuronal ceroid lipofuscinosis	
CLN3: juvenile neuronal ceroid lipofuscinosis	
Angelman syndrome	
CLN1 (INCL): infantile neuronal ceroid lipofuscinosis	
Infantile cerebellar-retinal degeneration	
PCH1B: pontocerebellar hypoplasia, EXOSC3-related	
CLN7: neuronal ceroid lipofuscinosis, Gypsy variant	
Aicardi-Goutières syndrome, AR	
CLN8: neuronal ceroid lipofuscinosis, Turkish variant	
NBIA2A: INAD: infantile neuroaxonal dystrophy, classic	

100%→

Most useful tests for this patient

Top tests ranked by usefulness in narrowing the differential, taking into account cost and treatability

Order	Test
☐	Bundle: Skin biopsy
☐	Tripeptidyl peptidase enzyme activity low in leukocytes and fibroblasts
☐	Macular degeneration or atrophy
☐	Lymphocytes with curvilinear profiles inclusions in EM
☐	ERG (electroretinogram) abnormal

Generated by SimulConsult® on 2 May 2018 13:49 using software of 20 March 2018 12:14 and database of 24 April 2018 7:07.
Disease incidence was used. Onset was ignored. Genome used.

SimulConsult

Family 2761

Summary for a 7 year old girl with:

Pertinent positive findings

* = required to be in diseases considered; onsets can be at an age, by an age, or unknown

Req'd	Onset	Finding	Pertinence
*	≤3y	Seizures with abnormal movements	
	≤3y	Autistic behavior	
*	≤3y	Hypotonia	
*	≤3y	Motor developmental delay	
*	≤3y	Intellectual disability	
			high→

Pertinent negative findings

Absent	Finding	Pertinence
X	CT or MRI: basal ganglia abnormalities	
		high→

Family history

Family history based on known clinical findings

1 of 1 brothers affected
Mother not affected
Father not affected
Consanguinity: From same village

Pertinent gene findings from the variant table

Severity scores shown on left, with maximum of 5, and potential compound heterozygotes marked with "C"

Severity	Finding	Pertinence
5C	PIGT gene variants (biallelic)	
2	SMARCB1 gene variant (monoallelic)	
2	ABCC8 gene variant (monoallelic)	
5	NALCN gene variant (monoallelic)	
3	CTCF gene variant (monoallelic)	
		high→

Differential diagnosis

Disease	Probability
Multiple congenital anomalies-hypotonia-seizures 3	
Creatine deficiency: GAMT creatine synthesis	
CMV, symptomatic congenital infection	
Chromosome 17q12 recurrent duplication syndrome	
Cohen syndrome	
Folate deficiency, cerebral	
Adenosuccinase lyase deficiency	
Angelman syndrome	
Dihydropyrimidine dehydrogenase deficiency	
EIEE9: epileptic encephalopathy, early infantile 9, PCDH19-associated	

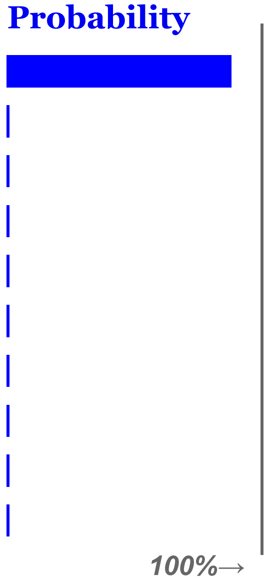

Most useful tests for this patient

Top tests ranked by usefulness in narrowing the differential, taking into account cost and treatability

Order	Test
☐	Calcium high in urine
☐	Alkaline phosphatase low in serum
☐	Glycosylphosphatidylinositol-linked proteins low on hematopoietic cells
☐	Bundle: UA (urinalysis)
☐	X-ray: bone age delayed

Generated by SimulConsult® on 2 May 2018 13:57 using software of 20 March 2018 12:14 and database of 24 April 2018 7:07.

Disease incidence was used. Onset was ignored. Genome used.

SimulConsult

Family 2887
Summary for a 11 year old boy with:

Pertinent positive findings

* = required to be in diseases considered; onsets can be at an age, by an age, or unknown

Req'd	Onset	Finding	Pertinence
*	≤3y	Gait disturbance	
*	≤3y	Hyperreflexia	
	≤3y	Spasticity character to hypertonia	
	≤3y	Hypertonia / stiffness	
	≤3y	Myoclonus	
	≤3y	Toe walking	
			high→

Pertinent negative findings

Absent	Finding	Pertinence
X	Seizures with abnormal movements	
X	CT or MRI: cerebral cortex atrophy or hypoplasia	
X	CT or MRI: corpus callosum hypogenesis	
X	Protein high in CSF	
X	CT or MRI: corpus callosum enlarged	
		high→

Family history

Family history based on known clinical findings

2 of 2 brothers affected
Mother not affected
Father not affected
Consanguinity: 2nd cousin

Pertinent gene findings from the variant table

Severity scores shown on left, with maximum of 5, and potential compound heterozygotes marked with "C"

Severity	Finding	Pertinence
5	ALS2 gene variants (biallelic)	
2	L1CAM gene variant (X-linked)	
2	CDKL5 gene variant (X-linked)	
4	TGIF1 gene variant (monoallelic)	
2C	WDPCP gene variants (biallelic)	
		high→

Differential diagnosis

Disease

- ALS2: ALS2-related amyotrophic lateral sclerosis, AR
- Juvenile primary lateral sclerosis
- Behr syndrome
- Gaucher disease type 3, subacute juvenile neuronopathic
- Dystonia, childhood-onset with optic atrophy and basal ganglia abnormalities
- Saposin C deficiency
- NBIA1: PKAN, classic
- PARK9: Kufor-Rakeb syndrome
- Spastic paraplegia and psychomotor retardation with or without seizures
- NBIA2A: INAD: infantile neuroaxonal dystrophy, classic

Probability

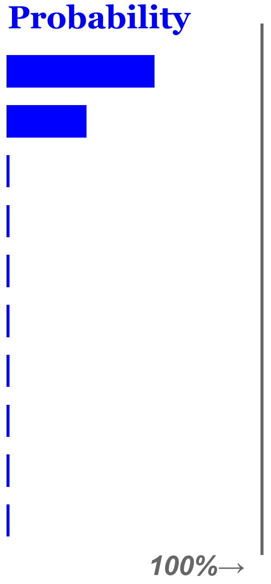

Most useful tests for this patient

Top tests ranked by usefulness in narrowing the differential, taking into account cost and treatability

Order	Test
☐	Bundle: EMG (electromyogram)
☐	EMG: neurogenic (denervation) changes
☐	Bundle: Muscle biopsy
☐	Muscle biopsy: neuropathic or denervation changes
☐	Bundle: MRI scan of the brain

Generated by SimulConsult® on 18 May 2018 15:53 using software of 15 May 2018 09:43 and database of 15 May 2018 7:25.
Disease incidence was ignored. Onset was used. Genome used.

SimulConsult

Family 2889

Summary for a 6 year old girl with:

Pertinent positive findings

* = required to be in diseases considered; onsets can be at an age, by an age, or unknown

Req'd	Onset	Finding	Pertinence
*	≤3y	Proteinuria	
	≤3y	CT or MRI: pan-cerebellar atrophy or hypoplasia	
*	≤3y	Ataxia	
*	≤3y	Intellectual disability	
*	≤3y	Motor developmental delay	
	≤3y	Rigidity character to hypertonia	
	≤Bir	Face, coarse	

high→

Pertinent negative findings

Absent	Finding	Pertinence
X	Seizures with abnormal movements	
X	ERG (electroretinogram) abnormal	

high→

Family history

Family history based on known clinical findings

1 of 2 sisters affected
0 of 2 brothers affected
Mother not affected
Father not affected
Consanguinity: 1st cousin

Pertinent gene findings from the variant table

Severity scores shown on left, with maximum of 5, and potential compound heterozygotes marked with "C"

Severity	Finding	Pertinence
5	WDR73 gene variants (biallelic)	
2	ITPR1 gene variant (monoallelic)	
2	RAI1 gene variant (monoallelic)	
2	FN1 gene variant (monoallelic)	
2C	SLC34A1 gene variants (biallelic)	

high→

Differential diagnosis

Disease	Probability
Galloway-Mowat syndrome 1, WDR73-related	
CDG1A: PMM2-related	
Galactosemia, classic	
Coenzyme Q10 deficiency, primary	
Wilson disease	
SCAR20: Spinocerebellar ataxia, AR, SNX14-related	
Mannosidosis, α, type 3: severe	
Pitt-Hopkins syndrome	
Salla disease	
MRXS35: mental retardation, X-linked syndromic, RPL10-related	

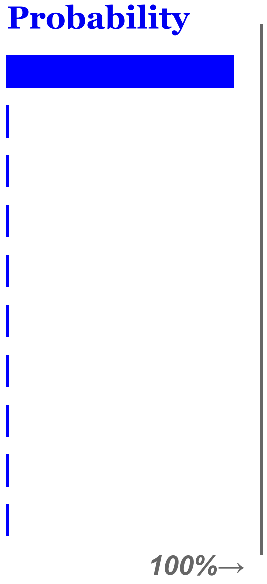

Most useful tests for this patient

Top tests ranked by usefulness in narrowing the differential, taking into account cost and treatability

Order	Test
☐	Nephrotic degree to proteinuria
☐	Albumin low in serum
☐	Triglycerides in serum high or false positive
☐	Bundle: Renal biopsy
☐	Renal structural abnormalities

Generated by SimulConsult® on 26 April 2018 16:05 using software of 20 March 2018 12:14 and database of 24 April 2018 7:07.

Disease incidence was used. Onset was ignored. Genome used.

SimulConsult

Family 2904

Summary for a 7 year old girl with:

Pertinent positive findings

* = required to be in diseases considered; onsets can be at an age, by an age, or unknown

Req'd	Onset	Finding	Pertinence
*	≤3y	Regression	
*	≤3y	Myoclonus	
	≤3y	Hypotonia	
	≤3y	Tremor of limbs, trunk or head	
	≤3y	Hyperreflexia	
	≤3y	Intellectual disability	
*	≤3y	Motor developmental delay	
	≤3y	Seizures with abnormal movements	
			high→

Family history

Family history based on known clinical findings

Mother not affected
Father not affected
Consanguinity: 1st cousin

Pertinent gene findings from the variant table

Severity scores shown on left, with maximum of 5, and potential compound heterozygotes marked with "C"

Severity	Finding	Pertinence
5	KCTD7 gene variants (biallelic)	
5	CASK gene variant (X-linked)	
3	DNM1L gene variant (monoallelic)	
3	KMT2B gene variant (monoallelic)	
2	RPS6KA3 gene variant (X-linked)	
		high→

Differential diagnosis

Disease

- EPM3: progressive myoclonic epilepsy 3
- EIEE6: Dravet syndrome (SCN1A-related)
- Angelman syndrome
- MTDPS4A: POLG-related mtDNA depletion
- CLN1 (INCL): infantile neuronal ceroid lipofuscinosis
- PARS2-related mtDNA depletion
- L2HGA: L-2-hydroxyglutaric aciduria
- EIEE37: early infantile epileptic encephalopathy, FRRS1L-related
- EIEE11: epileptic encephalopathy, early infantile, SCN2A-related
- Aicardi-Goutières syndrome, AR

Probability

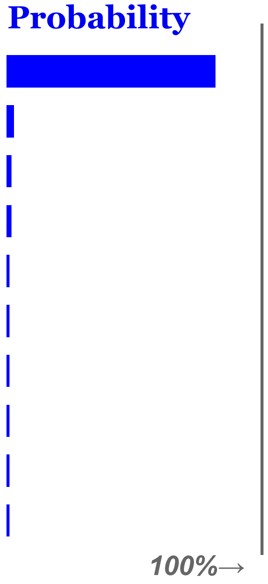

Most useful tests for this patient

Top tests ranked by usefulness in narrowing the differential, taking into account cost and treatability

Order	Test
☐	Bundle: EEG (electroencephalogram)
☐	Bundle: MRI scan of the brain
☐	Bundle: CT scan of the brain
☐	CT or MRI: cerebral cortex atrophy or hypoplasia
☐	Bundle: Nerve conduction studies

Generated by SimulConsult® on 2 May 2018 14:18 using software of 20 March 2018 12:14 and database of 24 April 2018 7:07.
Disease incidence was used. Onset was ignored. Genome used.

SimulConsult®

Family 3064

Summary for a 12 year old girl with:

Pertinent positive findings

* = required to be in diseases considered; onsets can be at an age, by an age, or unknown

Req'd	Onset	Finding	Pertinence
*	≤3y	Intellectual disability	
	≤3y	Myopia, severe	
	≤3y	Clitoris, large	
*	≤3y	Motor developmental delay	
*	≤3y	Hypotonia	
			high→

Family history

Family history based on known clinical findings

1 of 1 brothers affected
Mother not affected
Father not affected
Consanguinity: 1st cousin

Pertinent gene findings from the variant table

Severity scores shown on left, with maximum of 5, and potential compound heterozygotes marked with "C"

Severity	Finding	Pertinence
4	LINS1 gene variants (biallelic)	
5	NOTCH3 gene variant (monoallelic)	
2	GRIN1 gene variant (monoallelic)	
2	DNM1 gene variant (monoallelic)	
2	KCNB1 gene variant (monoallelic)	
		high→

Differential diagnosis

Disease

- MRT27: mental retardation, AR, LINS1-related
- Lehman lateral meningocele syndrome
- Kaufman oculocerebrofacial syndrome
- Cohen syndrome
- CDG1A: PMM2-related
- Pitt-Hopkins syndrome
- Muscular dystrophy-dystroglycanopathy A4
- Zellweger syndrome
- NS1: Noonan syndrome, PTPN11-related
- Spastic paraplegia and psychomotor retardation with or without seizures

Probability

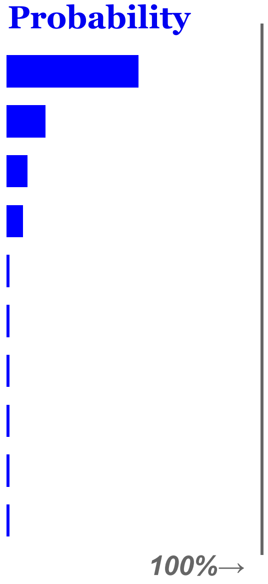

Most useful tests for this patient

Top tests ranked by usefulness in narrowing the differential, taking into account cost and treatability

Order	Test
☐	Bundle: Bone imaging
☐	X-ray: abnormal bone thickness or shape
☐	Bundle: Arthritis
☐	Bundle: MRI scan of the brain
☐	UBE3B gene variants (biallelic)

Generated by SimulConsult® on 2 May 2018 14:32 using software of 20 March 2018 12:14 and database of 24 April 2018 7:07.

Disease incidence was used. Onset was ignored. Genome used.

SimulConsult

Family 3127

Summary for a 8 year old girl with:

Pertinent positive findings

* = required to be in diseases considered; onsets can be at an age, by an age, or unknown

Req'd	Onset	Finding	Pertinence
*	≤3y	Gait disturbance	
*	≤3y	MRI: white matter abnormality	
	≤3y	Microcephaly	
	≤3y	Seizures with abnormal movements	
*	≤3y	Spasticity character to hypertonia	
*	≤3y	Hypertonia / stiffness	
*	≤3y	Motor developmental delay	
*	≤3y	Intellectual disability	

high→

Family history

Family history based on known clinical findings

1 of 1 brothers affected
Mother not affected
Father not affected
Consanguinity: 1st cousin

Pertinent gene findings from the variant table

Severity scores shown on left, with maximum of 5, and potential compound heterozygotes marked with "C"

Severity	Finding	Pertinence
4	AP4M1 gene variants (biallelic)	
3C	MECP2 gene variant (X-linked)	
2C	EIF2B5 gene variants (biallelic)	
3	CPS1 gene variants (biallelic)	
5	ABCA5 gene variants (biallelic)	

high→

Differential diagnosis

Disease	Probability
SPG50: spastic paraplegia 50	
Aicardi-Goutières syndrome, AR	
SPG51: spastic paraplegia 51	
GLUT1 deficiency syndrome 1, AR	
Folate deficiency, cerebral	
CLN1 (INCL): infantile neuronal ceroid lipofuscinosis	
PCH4: pontocerebellar hypoplasia, TSEN54-related	
GLUT1 deficiency syndrome 1, AD	
Polymicrogyria, bilateral frontoparietal	
CLN8: neuronal ceroid lipofuscinosis, Turkish variant	

100%→

Most useful tests for this patient

Top tests ranked by usefulness in narrowing the differential, taking into account cost and treatability

Order	Test
☐	Bundle: CT scan of the brain
☐	CT or MRI: pan-cerebellar atrophy or hypoplasia
☐	CT or MRI: cerebral cortex atrophy or hypoplasia
☐	X-ray or CT: brain calcifications
☐	WBC high in CSF

Generated by SimulConsult® on 27 April 2018 10:54 using software of 20 March 2018 12:14 and database of 24 April 2018 7:07.
Disease incidence was used. Onset was ignored. Genome used.

SimulConsult

Family 3130

Summary for a 11 year old girl with:

Pertinent positive findings

* = required to be in diseases considered; onsets can be at an age, by an age, or unknown

Req'd	Onset	Finding	Pertinence
*	≤1y	Motor developmental delay	
	≤Bir	Eyebrows highly arched	
	≤Bir	Eyes: blepharophimosis	
*	≤Bir	Mouth: palate cleft	
	≤Bir	Hypertelorism	
*	≤Bir	Microcephaly	
	≤Bir	Ears large	
*	≤3y	Intellectual disability	
	≤Bir	Ears low-set	
	≤3y	Attention deficit	

high→

Family history

Family history based on known clinical findings

1 of 2 sisters affected
1 of 2 brothers affected
Mother not affected
Father not affected
Consanguinity: 1st cousin

Pertinent gene findings from the variant table

Severity scores shown on left, with maximum of 5, and potential compound heterozygotes marked with "C"

Severity	Finding	Pertinence
5	UBE3B gene variants (biallelic)	
4	NOTCH1 gene variant (monoallelic)	
2	BCL11A gene variant (monoallelic)	
2	PAX3 gene variant (monoallelic)	
4C	TTN gene variants (biallelic)	

high→

Differential diagnosis

Disease	Probability
Kaufman oculocerebrofacial syndrome	
Marden-Walker syndrome	
Toriello-Carey syndrome	
Chromosome 1q43-q44 deletion syndrome	
CDLS1: Cornelia de Lange syndrome, NIPBL-related	
Chromosome 16p11.2 deletion syndrome, 593-kb	
Cerebrooculofacioskeletal syndrome	
Chromosome 3pter-p25 deletion syndrome	
CDLS4: Cornelia de Lange syndrome, RAD21-related	
Koolen-De Vries syndrome	
	100%→

Most useful tests for this patient

Top tests ranked by usefulness in narrowing the differential, taking into account cost and treatability

Order	Test
☐	Thyroid stimulating hormone (TSH) high in serum
☐	Cholesterol low in serum
☐	ACTH low in serum
☐	Bundle: Echocardiogram
☐	Growth hormone low in serum on stimulation test

Generated by SimulConsult® on 2 May 2018 14:39 using software of 20 March 2018 12:14 and database of 24 April 2018 7:07.
Disease incidence was used. Onset was ignored. Genome used.

Family 3158

Summary for a 4 year old girl with:

Pertinent positive findings

* = required to be in diseases considered; onsets can be at an age, by an age, or unknown

Req'd	Onset	Finding	Pertinence
*	≤1y	Hypotonia	
*	≤6m	Seizures with abnormal movements	
	✓	Intellectual disability	
	≤3y	Rigidity character to hypertonia	
			high→

Family history

Family history based on known clinical findings

0 of 1 sisters affected
2 of 2 brothers affected
Mother not affected
Father not affected
Consanguinity: 1st cousin

Pertinent gene findings from the variant table

Severity scores shown on left, with maximum of 5, and potential compound heterozygotes marked with "C"

Severity	Finding	Pertinence
5	MOCS2 gene variants (biallelic)	
5	CLCN6 gene variants (biallelic)	
4C	BCOR gene variant (X-linked)	
2	CASK gene variant (X-linked)	
2	FAM111A gene variant (monoallelic)	
		high→

Differential diagnosis

Disease | **Probability**

- Molybdenum cofactor deficiency, classical
- CLN1 (INCL): infantile neuronal ceroid lipofuscinosis
- EIEE37: early infantile epileptic encephalopathy, FRRS1L-related
- EIEE28: early infantile epileptic encephalopathy, WWOX-related
- MTDPS4A: POLG-related mtDNA depletion
- Neurodevelopmental disorder with progressive microcephaly, spasticity, and brain anomalies
- Creatine deficiency: GAMT creatine synthesis
- Cohen syndrome
- Chromosome 17q12 recurrent duplication syndrome
- PARS2-related mtDNA depletion

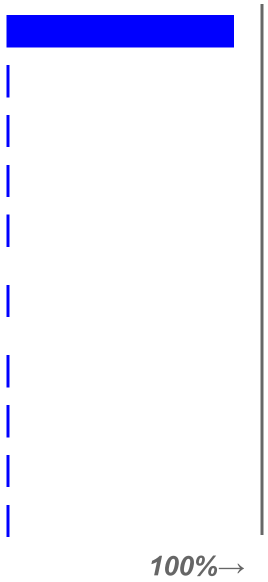

Most useful tests for this patient

Top tests ranked by usefulness in narrowing the differential, taking into account cost and treatability

Order	Test
☐	Sulfate in urine low
☐	Sulfite in fresh urine high
☐	Thiosulfate high in urine
☐	Total homocysteine low in plasma
☐	Xanthine oxidase low

Generated by SimulConsult® on 2 May 2018 14:52 using software of 20 March 2018 12:14 and database of 24 April 2018 7:07.

Disease incidence was used. Onset was ignored. Genome used.

SimulConsult

Family 3163

Summary for a 10 year old boy with:

Pertinent positive findings

* = required to be in diseases considered; onsets can be at an age, by an age, or unknown

Req'd	Onset	Finding	Pertinence
*	≤3y	Ataxia	
	≤3y	EMG: neurogenic (denervation) changes	
*	≤1y	Motor developmental delay	
*	≤Bir	Microcephaly	
	≤1y	Hypotonia	
*	≤Bir	CT or MRI: pan-cerebellar atrophy or hypoplasia	
*	≤3y	Intellectual disability	
			high→

Pertinent negative findings

Absent	Finding	Pertinence
X	Seizures with abnormal movements	
		high→

Family history

Family history based on known clinical findings

1 of 1 brothers affected
Mother not affected
Father not affected
Consanguinity: 1st cousin

Pertinent gene findings from the variant table

Severity scores shown on left, with maximum of 5, and potential compound heterozygotes marked with "C"

Severity	Finding	Pertinence
5	SIL1 gene variants (biallelic)	
3	SCN1A gene variant (monoallelic)	
3	NRXN1 gene variant (monoallelic)	
3	EPOR gene variant (monoallelic)	
3	PABPN1 gene >10 trinucleotide repeats (biallelic)	
		high→

Differential diagnosis

Disease | **Probability**

- Marinesco-Sjögren syndrome
- PCH1B: pontocerebellar hypoplasia, EXOSC3-related
- NBIA2A: INAD: infantile neuroaxonal dystrophy, classic
- PCH1A: pontocerebellar hypoplasia, VRK1-related
- SCAR2: Spinocerebellar ataxia, AR, congenital nonprogressive
- CDG1A: PMM2-related
- PCH11: pontocerebellar hypoplasia, TBC1D23-related
- PCH8: pontocerebellar hypoplasia, CHMP1A-related
- Pitt-Hopkins syndrome
- Williams syndrome

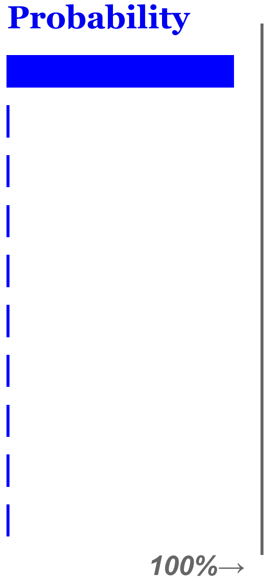

Most useful tests for this patient

Top tests ranked by usefulness in narrowing the differential, taking into account cost and treatability

Order	Test
☐	EMG: myopathic changes
☐	CT or MRI: vermal cerebellar atrophy or hypoplasia
☐	Bundle: Nerve conduction studies
☐	Creatine kinase high
☐	Bundle: Muscle biopsy

Generated by SimulConsult® on 2 May 2018 14:59 using software of 20 March 2018 12:14 and database of 24 April 2018 7:07.

Disease incidence was used. Onset was ignored. Genome used.

SimulConsult

Family 3172

Summary for a 4 year old boy with:

Pertinent positive findings

* = required to be in diseases considered; onsets can be at an age, by an age, or unknown

Req'd	Onset	Finding	Pertinence
	≤1y	Spasticity character to hypertonia	
	≤1y	CT or MRI: cerebral cortex atrophy or hypoplasia	
*	≤1y	Microcephaly	
*	≤1y	Seizures with abnormal movements	
*	≤1y	Intellectual disability	
*	≤1y	Motor developmental delay	
			high→

Family history

Family history based on known clinical findings

0 of 1 sisters affected
2 of 2 brothers affected
Mother not affected
Father not affected
Consanguinity: 2nd cousin

Pertinent gene findings from the variant table

Severity scores shown on left, with maximum of 5, and potential compound heterozygotes marked with "C"

Severity	Finding	Pertinence
5	PARS2 gene variants (biallelic)	
4	SMC3 gene variant (monoallelic)	
3	HDAC4 gene variant (monoallelic)	
4	TRIP13 gene variants (biallelic)	
4	JAK2 gene variant (monoallelic)	
		high→

Differential diagnosis

Disease	Probability
PARS2-related mtDNA depletion	
CLN1 (INCL): infantile neuronal ceroid lipofuscinosis	
MTDPS4A: POLG-related mtDNA depletion	
Menkes disease, classic	
CLN2: late-infantile neuronal ceroid lipofuscinosis	
Krabbe globoid cell leukodystrophy, infantile	
PCH2: pontocerebellar hypoplasia 2	
Galloway-Mowat syndrome 3, OSGEP-related	
Rhizomelic chondrodysplasia punctata type 1	
PCH4: pontocerebellar hypoplasia, TSEN54-related	

100%→

Most useful tests for this patient

Top tests ranked by usefulness in narrowing the differential, taking into account cost and treatability

Order	Test
☐	Bundle: Nerve conduction studies
☐	Transaminases (LFTs) high
☐	CT or MRI: cerebral gliosis
☐	Nerve conduction: NCV slow, sensory
☐	Nerve conduction: NCV slow, motor

Generated by SimulConsult® on 2 May 2018 15:06 using software of 20 March 2018 12:14 and database of 24 April 2018 7:07.
Disease incidence was used. Onset was ignored. Genome used.

SimulConsult

Family 3191

Summary for a 11 year old boy with:

Pertinent positive findings

* = required to be in diseases considered; onsets can be at an age, by an age, or unknown

Req'd	Onset	Finding	Pertinence
*	≤3y	Hyperactivity	
*	≤3y	CT or MRI: lissencephaly	
*	≤Bir	Microcephaly	
	≤3y	Attention deficit	
	≤3y	CT, MRI or head USG: hydrocephalus, not ex-vacuo	
*	≤3y	Motor developmental delay	
	≤3y	Hyperreflexia	
	≤3y	Hypertonia / stiffness	
	≤3y	Ataxia	
*	≤3y	CT or MRI: corpus callosum hypogenesis	
*	≤3y	Intellectual disability	

high→

Pertinent negative findings

Absent	Finding	Pertinence
X	Seizures with abnormal movements	

high→

Family history

Family history based on known clinical findings

0 of 1 sisters affected
0 of 1 brothers affected
Mother not affected
Father not affected
Consanguinity: 2nd cousin

Pertinent gene findings from the variant table

Severity scores shown on left, with maximum of 5, and potential compound heterozygotes marked with "C"

Severity	Finding	Pertinence
4	ASPM gene variants (biallelic)	
2C	RAB3GAP2 gene variants (biallelic)	
2	EP300 gene variant (monoallelic)	
2	TRIO gene variant (monoallelic)	
3	AHDC1 gene variant (monoallelic)	

high→

Differential diagnosis

Disease	Probability
MCPH5: microcephaly, primary AR, ASPM-related	
EIEE10: epileptic encephalopathy, early infantile, PNKP-related	
MCPH2: microcephaly, primary AR, WDR62-related	
Nijmegen breakage syndrome	
LIS3: TUBA1A-related lissencephaly, AD	
Fetal alcohol syndrome	
PCH11: pontocerebellar hypoplasia, TBC1D23-related	
MCPH19: microcephaly, primary, COPB2-related	
Galloway-Mowat syndrome 1, WDR73-related	
CHARGE syndrome	

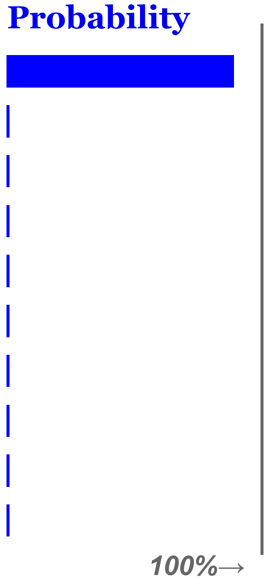

Most useful tests for this patient

Top tests ranked by usefulness in narrowing the differential, taking into account cost and treatability

Order	Test
☐	CT or MRI: cerebral cortex atrophy or hypoplasia
☐	CT or MRI: vermal cerebellar atrophy or hypoplasia
☐	Renal structural abnormalities
☐	MRI: white matter abnormality
☐	Bundle: Echocardiogram

Generated by SimulConsult® on 27 April 2018 11:17 using software of 20 March 2018 12:14 and database of 24 April 2018 7:07.
Disease incidence was used. Onset was ignored. Genome used.

SimulConsult

Family 3200

Summary for a 13 year old girl with:

Pertinent positive findings

* = required to be in diseases considered; onsets can be at an age, by an age, or unknown

Req'd	Onset	Finding	Pertinence
*	≤3y	Gait disturbance	
*	≤1y	Microcephaly	
	≤3y	Weakness, significant	
*	≤3y	MRI: white matter abnormality	
	≤3y	CT, MRI or head USG: hydrocephalus, not ex-vacuo	
	≤1y	Hyperreflexia	
*	≤3y	Spasticity character to hypertonia	
*	≤3y	Hypertonia / stiffness	
*	≤3y	Intellectual disability	
*	≤3y	Motor developmental delay	
	≤3y	EEG: periodic sharp wave complexes	

high→

Family history

Family history based on known clinical findings

0 of 1 sisters affected
1 of 3 brothers affected
Mother not affected
Father not affected
Consanguinity: 1st cousin

Pertinent gene findings from the variant table

Severity scores shown on left, with maximum of 5, and potential compound heterozygotes marked with "C"

Severity	Finding	Pertinence
4	AP4M1 gene variants (biallelic)	
2C	EIF2B5 gene variants (biallelic)	
5	AP4E1 gene variant (monoallelic)	
5	SAMD9 gene variants (biallelic)	
4	IGSF1 gene variant (X-linked)	

high→

Differential diagnosis

Disease	Probability
SPG50: spastic paraplegia 50	
Cockayne syndrome I, moderate or classic	
SPG51: spastic paraplegia 51	
Aicardi-Goutières syndrome, AR	
Folate deficiency, cerebral	
CLN1 (INCL): infantile neuronal ceroid lipofuscinosis	
SPG9B: spastic paraplegia, AR, ALDH18A1-related	
Krabbe globoid cell leukodystrophy, juvenile	
NBIA2A: INAD: infantile neuroaxonal dystrophy, classic	
Spastic paraplegia and psychomotor retardation with or without seizures	
	100%→

Most useful tests for this patient

Top tests ranked by usefulness in narrowing the differential, taking into account cost and treatability

Order	Test
☐	CT or MRI: pan-cerebellar atrophy or hypoplasia
☐	ERG (electroretinogram) abnormal
☐	Pigmentary retinopathy
☐	Bundle: Nerve conduction studies
☐	CT or MRI: cerebral cortex atrophy or hypoplasia

Generated by SimulConsult® on 2 May 2018 17:33 using software of 20 March 2018 12:14 and database of 24 April 2018 7:07.
Disease incidence was used. Onset was ignored. Genome used.

SimulConsult

Family 3208

Summary for a 7 year old girl with:

Pertinent positive findings

* = required to be in diseases considered; onsets can be at an age, by an age, or unknown

Req'd	Onset	Finding	Pertinence
*	≤1y	Cataracts	
*	≤1y	CT or MRI: cerebral cortex atrophy or hypoplasia	
	≤1y	Motor developmental delay	
	≤1y	Intellectual disability	
*	≤1y	Microcephaly	
	≤3y	Eye movement deficit, horizontal	
			high→

Family history

Family history based on known clinical findings

Mother not affected
Father not affected
Consanguinity: 1st cousin

Pertinent gene findings from the variant table

Severity scores shown on left, with maximum of 5, and potential compound heterozygotes marked with "C"

Severity	Finding	Pertinence
5	PHGDH gene variants (biallelic)	
2C	PEX26 gene variants (biallelic)	
5	IFIH1 gene variant (monoallelic)	
3	PHF6 gene variant (X-linked)	
2	CNKSR2 gene variant (X-linked)	
		high→

Differential diagnosis

Disease | **Probability**

- 3-phosphoglycerate dehydrogenase def., infantile
- CCFDN
- Chromosome 1p36 deletion syndrome
- Muscular dystrophy-dystroglycanopathy A4
- Neu-Laxova syndrome 1, PHGDH-related
- Chromosome 22q11.2 deletion syndrome
- Williams syndrome
- Neurodevelopmental disorder with microcephaly, hypotonia, & brain anomalies
- CMV, symptomatic congenital infection
- Hypomelanosis of Ito

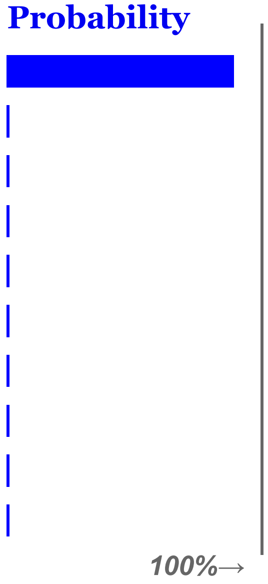

Most useful tests for this patient

Top tests ranked by usefulness in narrowing the differential, taking into account cost and treatability

Order	Test
☐	Bundle: Amino acids in CSF
☐	Bundle: Amino acids in plasma
☐	L-serine low in CSF
☐	D-serine low in CSF
☐	5-methyltetrahydrofolate low in CSF

Generated by SimulConsult® on 2 May 2018 15:51 using software of 20 March 2018 12:14 and database of 24 April 2018 7:07.

Disease incidence was used. Onset was ignored. Genome used.

SimulConsult

Family 3226

Summary for a 7 year old boy with:

Pertinent positive findings

* = required to be in diseases considered; onsets can be at an age, by an age, or unknown

Req'd	Onset	Finding	Pertinence
*	≤3y	Ataxia	
*	≤3y	CT or MRI: pan-cerebellar atrophy or hypoplasia	
*	≤3y	Regression	
*	≤3y	Motor developmental delay	
*	≤3y	Intellectual disability	
	≤3y	Hearing impairment	
	≤3y	CT or MRI: vermal cerebellar atrophy or hypoplasia	

high→

Pertinent negative findings

Absent	Finding	Pertinence
X	Seizures with abnormal movements	

high→

Family history

Family history based on known clinical findings

0 of 1 brothers affected
Mother not affected
Father not affected
Consanguinity: 1st cousin

Pertinent gene findings from the variant table

Severity scores shown on left, with maximum of 5, and potential compound heterozygotes marked with "C"

Severity	Finding	Pertinence
5	PLA2G6 gene variants (biallelic)	
2	ACO2 gene variants (biallelic)	
3	NPC1 gene variants (biallelic)	
3	EZH2 gene variant (monoallelic)	
5	TUBB3 gene variant (monoallelic)	

high→

Differential diagnosis

Disease	Probability
NBIA2B: neuroaxonal dystrophy, atypical	
NBIA2A: INAD: infantile neuroaxonal dystrophy, classic	
HLD6: hypomyelinating leukodystrophy, TUBB4A -related	
Marinesco-Sjögren syndrome	
Mannosidosis, α, type 2: moderate infantile	
Infantile cerebellar-retinal degeneration	
Angelman syndrome	
MECP2 duplication syndrome, classical	
Salla disease	
CMT6B: Charcot-Marie-Tooth, SLC25A46-related	

100%→

Most useful tests for this patient

Top tests ranked by usefulness in narrowing the differential, taking into account cost and treatability

Order	Test
☐	Bundle: Nerve conduction studies
☐	Nerve conduction: low amplitude, motor action potentials
☐	Nerve conduction: low amplitude sensory action potentials
☐	SER abnormal
☐	Bundle: EMG (electromyogram)

Generated by SimulConsult® on 2 May 2018 16:13 using software of 20 March 2018 12:14 and database of 24 April 2018 7:07.
Disease incidence was used. Onset was ignored. Genome used.

SimulConsult

Family 3310

Summary for a 4 year old boy with:

Pertinent positive findings

* = required to be in diseases considered; onsets can be at an age, by an age, or unknown

Req'd	Onset	Finding	Pertinence
	≤3y	Dystonia	
*	≤3y	MRI: periventricular predominance to brain imaging abnormality	
*	≤3y	CT or MRI: cerebral cortex atrophy or hypoplasia	
	≤3y	Irritability or agitation, pronounced	
*	≤3y	Spasticity character to hypertonia	
*	≤3y	Regression	
*	≤3y	Intellectual disability	
	≤3y	Periventricular or subependymal nature to intracerebral calcifications	

high→

Family history

Family history based on known clinical findings

1 of 1 brothers affected
Mother not affected
Father not affected
Consanguinity: 2nd cousin

Pertinent gene findings from the variant table

Severity scores shown on left, with maximum of 5, and potential compound heterozygotes marked with "C"

Severity	Finding	Pertinence
5	GALC gene variants (biallelic)	
2C	CNTNAP2 gene variants (biallelic)	
2	NYX gene variant (X-linked)	
2	SLC16A12 gene variant (monoallelic)	
2	RIT1 gene variant (monoallelic)	

high→

Differential diagnosis

Disease	Probability
Krabbe globoid cell leukodystrophy, infantile	
CLN1 (INCL): infantile neuronal ceroid lipofuscinosis	
Aicardi-Goutières syndrome, AR	
CLN2: late-infantile neuronal ceroid lipofuscinosis	
Niemann-Pick disease type C	
CLN3: juvenile neuronal ceroid lipofuscinosis	
Menkes disease, classic	
MLD, late infantile: metachromatic leukodystrophy	
D-glyceric aciduria	
PARS2-related mtDNA depletion	
	100%→

Most useful tests for this patient

Top tests ranked by usefulness in narrowing the differential, taking into account cost and treatability

Order	Test
☐	Galactocerebrosidase low in leukocytes or fibroblasts
☐	Bundle: Nerve conduction studies
☐	Protein high in CSF
☐	ABR abnormal
☐	Nerve conduction: NCV slow, sensory

Generated by SimulConsult® on 2 May 2018 16:20 using software of 20 March 2018 12:14 and database of 24 April 2018 7:07.
Disease incidence was used. Onset was ignored. Genome used.

SimulConsult

Family 3356

Summary for a 6 year old boy with:

Pertinent positive findings

* = required to be in diseases considered; onsets can be at an age, by an age, or unknown

Req'd	Onset	Finding	Pertinence
	≤3y	Myoclonus	
*	≤3y	ERG (electroretinogram) abnormal	
*	≤3y	CT or MRI: cerebral cortex atrophy or hypoplasia	
*	≤3y	Motor developmental delay	
*	≤3y	Regression	
*	≤3y	Intellectual disability	
			high→

Pertinent negative findings

Absent	Finding	Pertinence
X	CT or MRI: pan-cerebellar atrophy or hypoplasia	
		high→

Family history

Family history based on known clinical findings

1 of 1 brothers affected
Mother not affected
Father not affected
Consanguinity: 3rd cousin

Pertinent gene findings from the variant table

Severity scores shown on left, with maximum of 5, and potential compound heterozygotes marked with "C"

Severity	Finding	Pertinence
5	PPT1 gene variants (biallelic)	
2	TRAPPC6B gene variants (biallelic)	
5	KATNB1 gene variants (biallelic)	
5C	WDR81 gene variants (biallelic)	
5	ZNF469 gene variant (monoallelic)	
		high→

Differential diagnosis

Disease	Probability
CLN1 (INCL): infantile neuronal ceroid lipofuscinosis	
CLN3: juvenile neuronal ceroid lipofuscinosis	
D-bifunctional peroxisomal enzyme deficiency	
Hyperornithinemia-hyperammonemia-homocitrullinuria syndrome	
CLN5: neuronal ceroid lipofuscinosis, Finnish variant	
CLN2: late-infantile neuronal ceroid lipofuscinosis	
Muscular dystrophy-dystroglycanopathy B6	
NARP syndrome	
Cockayne syndrome I, moderate or classic	
EIEE6: Dravet syndrome (SCN1A-related)	
	100%→

Most useful tests for this patient

Top tests ranked by usefulness in narrowing the differential, taking into account cost and treatability

Order	Test
☐	Bundle: Skin biopsy
☐	Palmitoyl-protein thioesterase activity low in leukocytes or fibroblasts
☐	EM: lymphocytes with granular osmophilic deposits inclusions on electron microscopy
☐	Skin biopsy: granular osmophilic deposits inclusions in EM
☐	Skin biopsy: abnormal morphology

Generated by SimulConsult® on 2 May 2018 16:24 using software of 20 March 2018 12:14 and database of 24 April 2018 7:07.
Disease incidence was used. Onset was ignored. Genome used.

SimulConsult

Family 3818

Summary for a 6 year old boy with:

Pertinent positive findings

* = required to be in diseases considered; onsets can be at an age, by an age, or unknown

Req'd	Onset	Finding	Pertinence
*	≤3y	Microcephaly	
*	≤3y	Spasticity character to hypertonia	
	≤3y	Motor developmental delay	
	≤3y	X-ray or CT: brain calcifications	
*	≤3y	Hypertonia / stiffness	
	≤3y	Hyperreflexia	
	≤3y	Intellectual disability	

high→

Family history

Family history based on known clinical findings

1 of 4 nearby contacts affected
Mother not affected
Father not affected
Consanguinity: 1st cousin

Pertinent gene findings from the variant table

Severity scores shown on left, with maximum of 5, and potential compound heterozygotes marked with "C"

Severity	Finding	Pertinence
4	PCDH12 gene variants (biallelic)	
5	FLNA gene variant (X-linked)	
5	LYRM7 gene variants (biallelic)	
2	STN1 gene variants (biallelic)	
2C	AARS2 gene variants (biallelic)	

high→

Differential diagnosis

Disease	Probability
Microcephaly, seizures, spasticity, and brain calcification	
Toxoplasmosis, symptomatic congenital infection	
Phenylketonuria	
Aicardi-Goutières syndrome, AR	
MTDPS4A: POLG-related mtDNA depletion	
EIEE49: early infantile epileptic encephalopathy, DENND5A-related	
Hemorrhagic destruction of the brain, subependymal calcification, and cataracts	
PARS2-related mtDNA depletion	
Hyperornithinemia-hyperammonemia-homocitrullinuria syndrome	
Leukoencephalopathy, cystic, without megalencephaly	

100%→

Most useful tests for this patient

Top tests ranked by usefulness in narrowing the differential, taking into account cost and treatability

Order	Test
☐	Bundle: MRI scan of the brain
☐	Periventricular or subependymal nature to intracerebral calcifications
☐	MRI: white matter abnormality
☐	Protein high in CSF
☐	Bundle: EEG (electroencephalogram)

Generated by SimulConsult® on 2 May 2018 17:02 using software of 20 March 2018 12:14 and database of 24 April 2018 7:07.
Disease incidence was used. Onset was ignored. Genome used.

SimulConsult

Family 4101

Summary for a 12 year old girl with:

Pertinent positive findings

* = required to be in diseases considered; onsets can be at an age, by an age, or unknown

Req'd	Onset	Finding	Pertinence
	≤1y	Weakness, significant	
*	≤1y	CT or MRI: cerebral cortex atrophy or hypoplasia	
*	≤1y	CT or MRI: basal ganglia abnormalities	
	≤1y	Stature short	
	≤1y	MRI: increased signal in basal ganglia	
	≤6m	Weight low or weight loss	
*	≤1y	MRI: white matter abnormality	
	≤1y	Hyperreflexia	
*	≤3y	Motor developmental delay	
*	≤3y	Intellectual disability	

high→

Pertinent negative findings

Absent	Finding	Pertinence
X	Seizures with abnormal movements	

high→

Family history

Family history based on known clinical findings

1 of 1 brothers affected
Mother not affected
Father not affected
Consanguinity: 1st cousin

Pertinent gene findings from the variant table

Severity scores shown on left, with maximum of 5, and potential compound heterozygotes marked with "C"

Severity	Finding	Pertinence
5	SUCLA2 gene variants (biallelic)	
4	ASNS gene variants (biallelic)	
2	FBN1 gene variant (monoallelic)	
2	FOXP1 gene variant (monoallelic)	
2C	OTOG gene variants (biallelic)	

high→

Differential diagnosis

Disease	Probability
MTDPS5: SUCLA2-related mtDNA depletion syndrome	
HLD14: leukodystrophy, hypomyelinating, UFM1-related	
Arginase deficiency	
2,4-dienoyl-CoA reductase deficiency	
Methemoglobinemia type II	
Glutaric aciduria I, classical	
CLN1 (INCL): infantile neuronal ceroid lipofuscinosis	
Cerebrotendinous xanthomatosis	
Leigh-like syndrome	
Asparagine synthetase deficiency	

100%→

Most useful tests for this patient

Top tests ranked by usefulness in narrowing the differential, taking into account cost and treatability

Order	Test
☐	Bundle: Organic acids in urine
☐	Methylmalonic acid high in urine
☐	Methylmalonylcarnitine high in urine
☐	Methylmalonic acid high in serum
☐	Bundle: Nerve conduction studies

Generated by SimulConsult® on 2 May 2018 17:12 using software of 20 March 2018 12:14 and database of 24 April 2018 7:07.

Disease incidence was used. Onset was ignored. Genome used.
